# Unraveling the Contributions to Spin–Lattice Relaxation in Kramers Single-Molecule Magnets

**DOI:** 10.1021/jacs.2c08876

**Published:** 2022-12-09

**Authors:** Sourav Mondal, Alessandro Lunghi

**Affiliations:** School of Physics, AMBER and CRANN Institute, Trinity College, Dublin2, Ireland

## Abstract

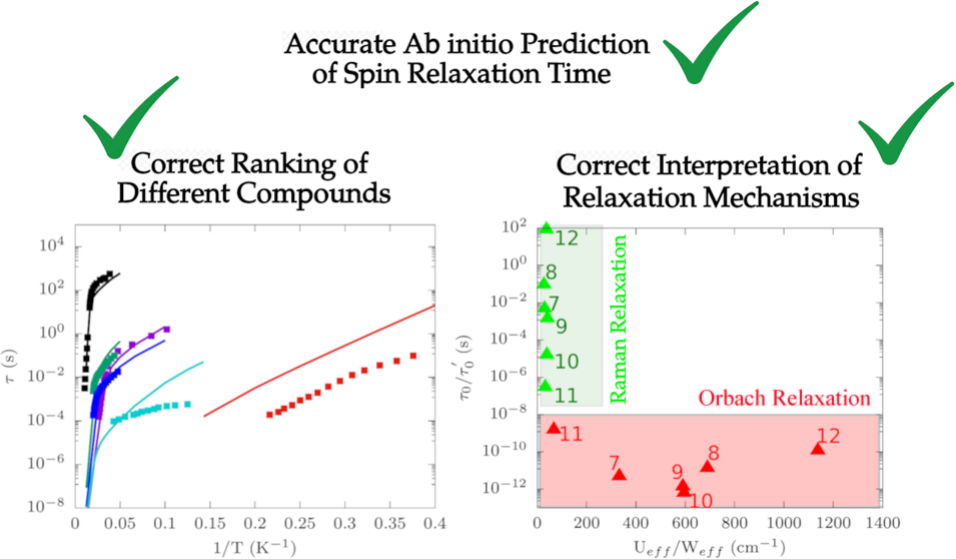

The study of how spin interacts with lattice vibrations and relaxes to equilibrium provides unique insights into its chemical environment and the relation between electronic structure and molecular composition. Despite its importance for several disciplines, ranging from magnetic resonance to quantum technologies, a convincing interpretation of spin dynamics in crystals of magnetic molecules is still lacking due to the challenging experimental determination of the correct spin relaxation mechanism. We apply *ab initio* spin dynamics to a series of 12 coordination complexes of Co^2+^ and Dy^3+^ ions selected among ∼240 compounds that largely cover the literature on single-molecule magnets and well represent different regimes of spin relaxation. Simulations reveal that the Orbach spin relaxation rate of known compounds mostly depends on the ions’ zero-field splitting and little on the details of molecular vibrations. Raman relaxation is instead found to be also significantly affected by the features of low-energy phonons. These results provide a complete understanding of the factors limiting spin lifetime in single-molecule magnets and revisit years of experimental investigations by making it possible to transparently distinguish Orbach and Raman relaxation mechanisms.

## Introduction

Coordination compounds of first-row transition metals and lanthanide ions offer a vast playground for the exploration of electronic and magnetic properties for applications ranging from catalysis^1^ and sensors^2^ to luminescence.^3,4^ In particular, molecules showing magnetic properties due to the presence of unpaired d/f electrons are under intense scrutiny for applications in the areas of information storage,^5^ spintronics,^6^ and quantum science.^7−9^ However, the delivery of molecule-based technologies strongly relies on the possibility to overcome their short spin lifetime. Similarly to hard ferromagnets,^10^ coordination compounds possessing easy-axis magnetic anisotropy are known to exhibit long spin relaxation time, hence their name single-molecule magnets (SMMs).^11^ Unless cryogenic temperatures are achieved, the interaction between spin and phonons, namely, the spin-phonon coupling, is mainly responsible for magnetic moment relaxation.^12^ Early studies^5,13^ have shown that spin relaxation time, τ, of SMMs follows an Arrhenius-like law
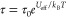
1where for a given temperature, *T*, the preexponential factor τ_0_ set the relaxation time scale and the *U*_eff_ represents an effective magnetic moment reversal barrier due to the presence of magnetic anisotropy. *U*_eff_ is intimately connected to the electronic structure of magnetic ions, and it coincides with the energy of the electronic excited state promoting relaxation through the absorption and emission of a series of phonons,^12^*i.e.*, the Orbach relaxation mechanism. Increasing *U*_eff_ has been the main strategy to improve τ and many efforts have been devoted to engineering coordination compounds with large zero-field splittings.^13,14^ The most successful strategy employs the use of Co^2+^ or Dy^3+^ ions with a strong and axial crystal field as building blocks for SMMs.^15^ Record values of 450 and 1541 cm^–1^ have been reached for single-ion complexes of Co^2+^ and Dy^3+^, respectively.^16,17^

Now that the limit in crystal field axiality has been virtually reached,^17^ new strategies toward high-temperature SMMs are required. Coupling multiple ions stands as one possible route, and important milestones in this direction have been achieved.^18−20^ Another strategy instead requires us to look at the entire spin-phonon relaxation process to determine other physical quantities that influence it. Here, we pursue the latter approach.

**Figure 1:**
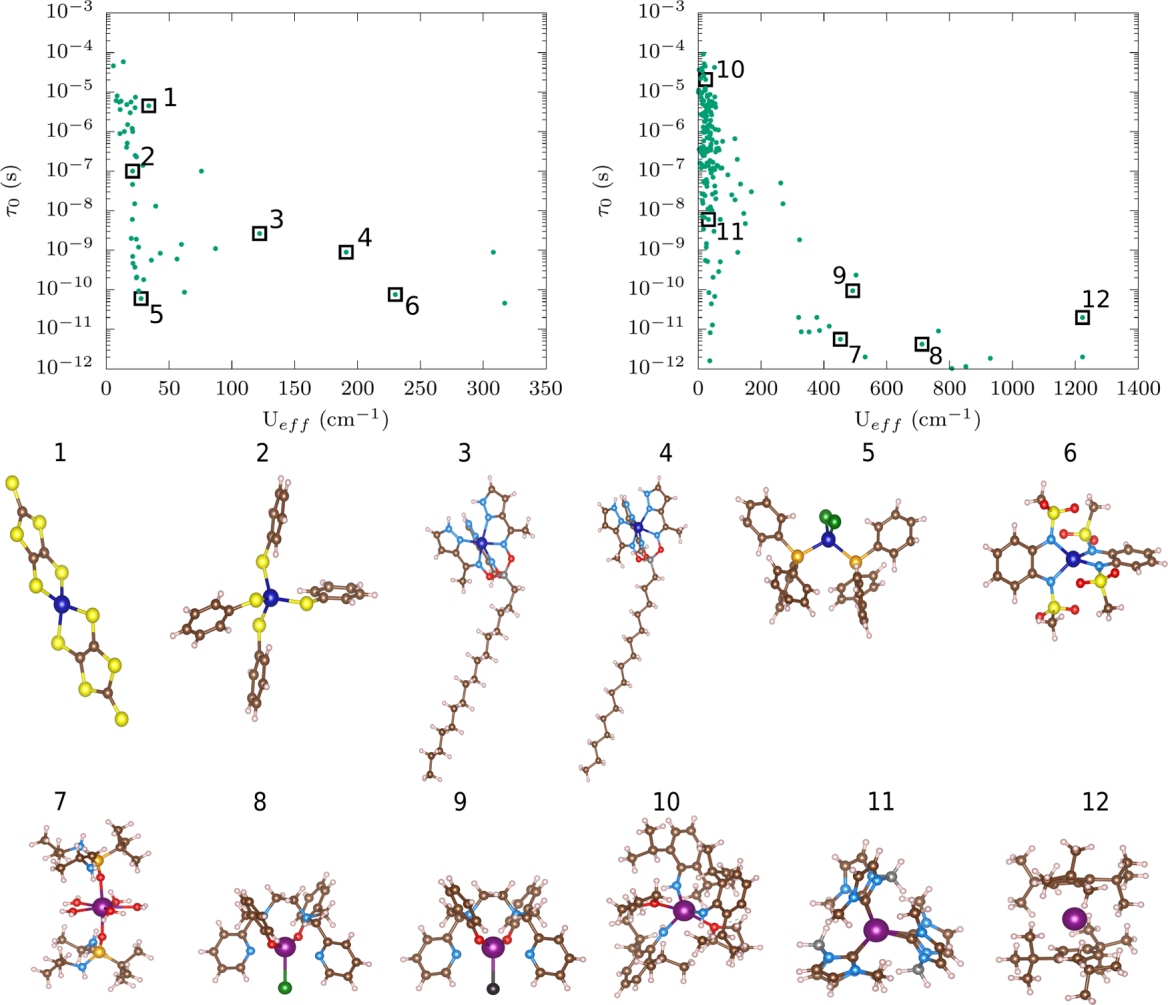
Experimental correlations and molecular structures. The top left and top right panels report the experimental τ_0_*vsU*_eff_ for a set of ∼240 single-ion Co^2+^ and Dy^3+^ complexes, respectively, individuated by scraping the literature on single-molecule magnets or from the SIMDAVIS database.^21^ Black squares are used to identify the 12 molecules selected for the study. The middle panel reports the molecular geometries of selected six Co^2+^ complexes **1**–**6**. The bottom panel reports the molecular geometries of selected six Dy^3+^ complexes **7**–**12**. Color codes for atoms: Dy in purple, Co in indigo, N in light blue, O in red, B in gray, Br in dark green, C in dark brown, S in yellow, P in gold, Cl in black, H in pale pink.

A striking example of how our limited knowledge of spin-phonon relaxation has impacted the field of SMMs comes from the visualization of the correlation between τ_0_ and *U*_eff_. Figure 1 (top) reports these two quantities for ∼240 single-ion Co^2+^ and Dy^3+^ SMMs that largely cover the relevant literature until early 2019 and highlights how τ_0_ spans 8 orders of magnitude and strongly correlates with *U*_eff_, undercutting the large values achieved for the latter. This is a result of the fact that despite the many efforts to control *U*_eff_, no clear insight into how to chemically control the preexponential factor τ_0_ is yet available and no attempts in optimizing it have ever been made. Moreover, as values of *U*_eff_ above 30 K had been reported, strong deviations from eq 1 have been observed and attributed to Raman relaxation,^22,23^*i.e.*, a process involving spin transitions due to the simultaneous absorption and emission of two phonons. Only recently theoretical models have been able to shine some light on the nature of this mechanism, showing that Raman mechanisms can also present an Arrhenius-like behavior under certain conditions.^24−26^ This situation poses an important challenge. Although experimental and computational strategies have been designed to disentangle Orbach and Raman relaxation,^27,28^ discerning the two mechanisms is far from trivial and misinterpretation of the fitted parameters in eq 1 has been suggested.^25,27−31^ Understanding the nature of these contributions to spin relaxation and their dependency on chemical structure is a fundamental step toward controlling spin-phonon coupling and delivering improved SMMs.

Here, we aim at providing a deeper understanding of the contributions to spin-phonon relaxation in single-molecule magnets with the goal of removing ambiguities in the interpretation of experiments and conclusively establishing what determines the rate of relaxation time. To achieve this, we exploit our recently developed *ab initio* spin dynamics approach, where the time evolution of the molecular magnetic moment under the influence of phonons is predicted from first principles and without the need of any information from the experiments except for the crystal structure.^12,24,26,32,33^ It has been demonstrated that this method can quantitatively describe both one- and two-phonon processes, responsible for Orbach and Raman relaxation mechanisms, across the entire relevant temperature range.^12^ Here we apply this strategy to a total of 12 Co^2+^ and Dy^3+^ SMMs. These compounds are selected from all of the ∼240 single-ion SMMs identified in the literature^21^ to sample different regimes of τ_0_*vsU*_eff_ and provide an unprecedented benchmark for both simulations and experiments.

We show that the correlation between τ_0_ and *U*_eff_ arising from the literature is only virtual and due to years of misinterpretation of Raman relaxation mechanism as Orbach. Thanks to the access to all of the details of spin relaxation, we demonstrate that the variance in the Orbach relaxation time among different SMMs is largely determined by the static crystal field splitting, while Raman relaxation time is also dependent on the details of molecular vibrations and spin-phonon coupling, providing a revised road map for the design of improved SMMs.

## Computational Methods

### Molecules Selection

The SIMDAVIS database has been used to gather a set of 183 Dy^3+^ single-ion SMMs and their respective relaxation data.^21^ A manual search for the corresponding Co^2+^ single-ion SMMs published until early 2019 has instead been carried out and resulted in 56 compounds. Table S1 reports all of the references, *U*_eff_ and τ_0_ for the Cobalt SMMs. Although our analysis does not account for the literature in its entirety, the selected compounds represent the entire range of relaxation regimes. Twelve molecules were selected from this data set following these criteria as closely as possible: (i) reported relaxation data span the entire range of τ_0_*vsU*_eff_, (ii) molecules are chemically and structurally diverse. The six Co^2+^ and six Dy^3+^ molecules chosen for this study are [Co(C_3_S_5_)_2_](Ph_4_P)_2_^34^ (**1**), [Co(SPh)_4_](Ph_4_P)^23^ (**2**), β-Co^35^ (**3**), α-Co^35^ (**4**), [Co(PPh_3_)_2_Br_2_]^36^ (**5**), [CoL_2_][(HNEt_3_)_2_],^37^ where H_2_L = 1,2-bis(methane-sulfonamido)benzene (**6**), [L_2_Dy(H_2_O)_5_][I]_3_·L_2_·H_2_O^38^ (**7**) where L= ^*t*^BuPO(NH^*i*^Pr)_2_, [Dy(bbpen)Br]^39^ (**8**) where H_2_bbpen = *N*,*N*′-bis(2-hydroxybenzyl)-*N*,*N*′-bis(2-methylpyridyl)ethylenediamine, [Dy(bbpen)Cl]^39^ (**9**), Dy[NHPh^*i*^Pr_2_]_3_(THF)_2_^40^ (**10**), Dy(Bc^Me^)_3_^41^ (**11**) where [Bc^Me^]^−^ = dihydrobis(methylimidazolyl)borate, and Dy[Cp_2_^ttt^][B(C_6_F_5_)_4_]^42^ (**12**), where (Cp^ttt^ = C_5_H_5_Bu_3_-1,2,4). The chemical structure of **1**–**12** is reported in Figure 1(bottom). Compounds **1**–**12** all respect the criteria (i) and (ii) except for pairs **3**–**4** and **8**–**9**, which have instead been chosen to challenge *ab initio* spin dynamics over minimal structural variations.

### Electronic Structure Simulations

Cell and geometry optimization and simulations of Γ-point phonons have been performed with periodic density functional theory (pDFT) using the software CP2K.^43^ Cell optimization was performed employing a very tight force convergence criteria of 10^–7^ au and SCF convergence criteria of 10^–10^ au for the energy. A plane wave cutoff of 1000 Ry, DZVP-MOLOPT Gaussian basis sets, and Goedecker–Teter–Hutter pseudopotentials^44^ were employed for all atoms. The Perdew–Burke–Ernzerhof (PBE) functional and DFT-D3 dispersion corrections were used.^45,46^

ORCA^47^ had been used to compute the magnetic properties. The magnetic properties of Dy^3+^ ions were computed from the CASSCF calculations employing active space of 7 4f orbitals with 9 electrons (9,7) and using all of the solutions with multiplicity 6, 224 solutions with multiplicity 4, and 490 solutions with multiplicity 2. Similarly, for Co^2+^ ions, magnetic properties were computed from the CASSCF calculations employing active space of 5 3d orbitals with 7 electrons (7,5) and using all of the solutions with multiplicity 4, and 40 solutions with multiplicity 2. The RIJCOSX approximation for coulomb and exchange integrals with integration grid GridX6 was used for both ions. The basis sets DKH-def2-QZVPP for Co atoms, DKH-def2-SVP for H, and SARC2-DKH-QZVP for Dy atoms were used. DKH-def2-TZVPP basis set has been used for the rest of the atoms present in the systems.

### Spin-Phonon Coupling and Relaxation Simulations

First-order spin-phonon coupling coefficients (∂*Ĥ*_s_/∂*Q*_α_) are computed as
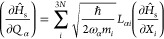
2where *Q*_α_ is the displacement vector associated with the α-phonon and *N* is the number of atoms in the unit cell, and *L*_α*i*_ and ω_α_ are the Hessian matrix eigenvectors and the phonon’s angular frequency, respectively. Only Γ-point phonons are used. The first-order derivatives of the spin Hamiltonian with respect to the Cartesian degree of freedom *X*_*i*_, (∂*Ĥ*_s_/∂*X*_*i*_), are computed by numerical differentiation.^32^ Each molecular degree of freedom is sampled 8 times between ±0.08 Å. Spin-phonon coupling coefficients are used to calculate the spin-phonon relaxation time on the basis of Redfield equations.^12,24,33^ Second- and fourth-order density matrix time-dependent perturbation theory have been used to simulate both one- and two-phonon processes. The software MolForge is used for these simulations and it is freely available at github.com/LunghiGroup/MolForge.^12^ As discussed elsewhere, the simulation of Kramers systems in zero external field requires the use of the nondiagonal secular approximation, where population and coherence terms of the density matrix are not independent of one another. This is achieved by simulating the dynamics of the entire density matrix for one-phonon processes.^12,33^ An equation that accounts for the dynamics of the entire density matrix under the effect of two-phonon processes resulting from fourth-order time-dependent perturbation theory is not yet available. However, it is possible to remove the coupling between population and coherence terms by orienting the molecular easy axis along the quantization *z*-axis and by applying a small magnetic field to break Kramers degeneracy.^12^ Here, we employ the latter strategy to simulate Raman relaxation.

## Results

### *Ab Initio* Spin Dynamics

Figure 1 (bottom) shows the molecular structure of the six Co^2+^ and six Dy^3+^ molecules chosen for this study. The corresponding values of τ and *U*_eff_ are highlighted in Figure 1 (top), showing the large span of values of these quantities. Compounds **6** and **12** are among the SMMs with the highest values of *U*_eff_, while **1** and **10** are reported to have a very large τ_0_ and a small *U*_eff_. These compounds also show a varied coordination chemistry. For instance, **1**, **2**, **5**, and **6** possess a tetrahedrally coordinated Co^2+^ ion, while **3** and **4** show octahedral coordination. Similarly, molecules **10** and **11** among the Dy^3+^ complexes show a coordination number of 6 and molecule **7** shows a coordination number of seven. On the other hand, compounds **8**, **9**, and **12** show the coordination number of three and bis-η^5^ (through the cyclopentadienyl group), respectively. The chosen compounds also show a varied set of ligands and charge states. The Co^2+^ complexes present a metal ion coordinated by N or S donor atoms in most cases, except in **5** where Co is bonded by Br and P donor atoms. Dy^3+^ also presents a varied coordination, including oxygen, nitrogen, halide ions, and metal–organic bonds. In most cases, the molecular unit is charged, except for **5** which is neutral. For instance, **1**, **2**, and **6** are dianionic, and **3** and **4** are mono-positive cations. On the contrary, most of the Dy complexes are neutral, except **7** and **12**, which are both cations.

With exception of **6** and **12** that were studied in a previous work,^12,24^ all of the unit cells of these compounds were optimized with pDFT, as described in the Computational Methods section. Electronic structure simulations at the level of CASSCF are then carried out on all isolated molecular structures with the coordinates fixed to the pDFT optimized value. The magnetic properties of all of the compounds are then computed by mapping electronic structure results onto effective Hamiltonians. The effective spin Hamiltonian
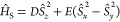
3is used to describe the ground state of all Co^2+^ compounds, while an effective crystal field Hamiltonian is used for Dy^3+^ compounds
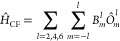
4where the operators *Ô*_*m*_^*l*^ are a tesseral function of the total angular momentum operators, **J⃗**. We will refer to any of the two operators with *Ĥ*_0_ in the following. All of the studied Co^2+^ compounds are found to exhibit an *S* = 3/2 ground state with uniaxial anisotropy (*D* < 0), while the Dy^3+^ compounds show an *M*_*j*_ = ±15/2 ground-state Kramers doublet (KD) well separated in energy from the excited KDs; see Table 1 and Table S4 in the Supporting Information (SI).

**Table 1:** Simulated Energy Data for Kramer’s Doublets^a^

system	Δ_01_ (cm^–1^)	Δ_07_ (cm^–1^)
**1**	273.51	
**2**	93.44	
**3**	199.56	
**4**	224.78	
**5**	20.06	
**6**	198.08	
**7**	293.84	706.85
**8**	394.41	864.21
**9**	370.51	810.70
**10**	240.94	968.38
**11**	10.55	520.95
**12**	451.00	1473.01

a Δ_01_ represents the difference of energy between the first excited state and the ground-state Kramers doublets, and Δ_07_ represents the difference of energy between the last and the ground-state Kramers doublets in Dy^3+^ compounds.

Once the eigenstates, |*a*⟩, and eigenvalues, *E*_*a*_, of these operators have been obtained, spin dynamics can be simulated by computing the transition rate among different spin states, *W*_*ab*_. Spin relaxation in molecular Kramers systems with large magnetic anisotropy takes contributions from one- and two-phonon processes. Considering one-phonon processes, the transition rate, *Ŵ*_*ba*_^1-ph^, among spin states reads
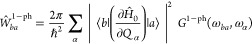
5where ℏω_*ba*_ = *E*_b_ – *E*_a_ and the term (∂*Ĥ*_0_/∂*Q*_α_) provides the intensity of the coupling between spin and the α-phonon *Q*_α_. The function *G*^1-ph^ reads
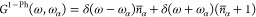
6where *n̅*_α_ = (exp(ℏω_α_/*k*_B_*T*) – 1)^−1^ is the Bose–Einstein distribution accounting for the phonons’ thermal population, *k*_B_ is the Boltzmann constant, and the Dirac δ functions enforce energy conservation during the absorption and emission of phonon by the spin system, respectively. Equation 5 accounts for the Orbach relaxation mechanism, where a series of phonon absorption processes leads the spin from the fully polarized state *M*_s_ = *S* to an excited state with an intermediate value of *M*_s_ before the spin can emit phonons back to *M*_s_ = −*S*, and similarly for states characterized by the total angular momentum, *J*.

Two-phonon processes provide an alternative pathway of relaxation to equilibrium, namely, the Raman mechanism. We model two-phonon spin-phonon transitions, *W*_*ba*_^2-ph^, as
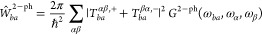
7where the terms
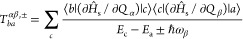
8involve the contribution of all of the spin states |*c*⟩ at the same time, often referred to as a virtual state. The function *G*^2-ph^ fulfills a similar role to *G*^1-ph^ for one-phonon processes and includes contributions from the Bose–Einstein distribution and imposes energy conservation. *G*^2-ph^ accounts for all two-phonon processes, *i.e.*, absorption of two phonons, emission of two phonons, or absorption of one phonon and emission of a second one. The latter process is the one that determines Raman relaxation rate, and in this case, *G*^2-ph^ reads
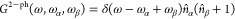
9

All of the parameters appearing in eqs 5 and 7 are computed from first principles (see the Computational Methodssection). In a nutshell, lattice harmonic frequencies, ω_α_/2π, and normal modes, *Q*_α_, are computed by finite differentiation after geometry optimization with pDFT. All of the parameters appearing in eqs 3 and 4, *i.e.*, *D*, *E*, and *B*_*m*_^*l*^, are numerically differentiated with respect to the atomic displacements defined by *Q*_α_ to obtain the spin-phonon coupling coefficients (∂*Ĥ*_s_/∂*Q*_α_). Once all of the matrix elements *W*_*ba*_^*n*-ph^ have been computed, τ^–1^ can be predicted by simply diagonalizing *W*_*ba*_^*n*-ph^ and taking the smallest nonzero eigenvalue. The study of *W*^1-ph^ provides the Orbach contribution to the relaxation rate, τ_Orbach_^–1^, while *W*^2-ph^ provides the Raman contribution, τ_Raman_^–1^. The total relaxation time is thus computed as τ^–1^ = τ_Orbach_^–1^ + τ_Raman_^–1^.

**Figure 2:**
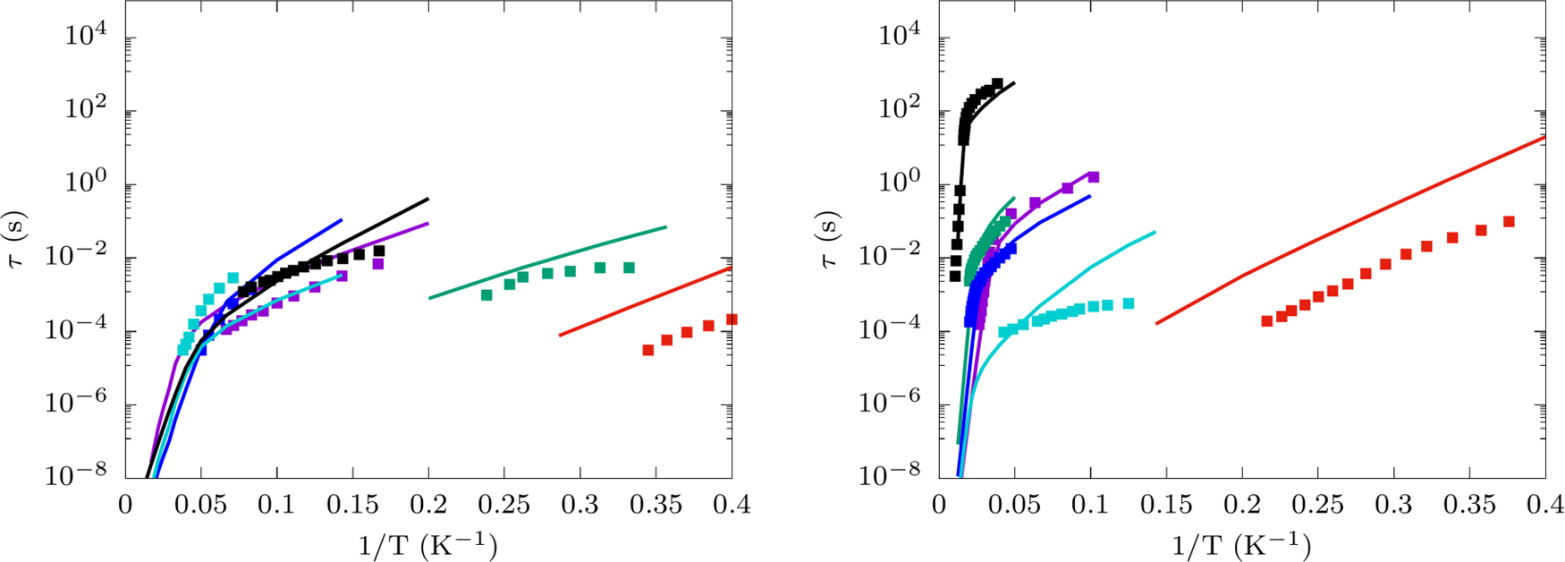
Spin-phonon relaxation times. Simulated values of τ are reported with continuous lines, while experimental values are reported with solid square symbols. Color code for left panel: 1 (violet), 2 (green), 3 (blue), 4 (turquoise), 5 (red), and 6 (black). Color code for right panel: 7 (violet), 8 (green), 9 (blue), 10 (turquoise), 11 (red), and 12 (black).

Figure 2 reports the experimental values and the simulations results for τ as a function of temperature for both Co^2+^ and Dy^3+^ complexes. Overall simulations reproduce experimental results very well and they prove capable of reproducing trends in relaxation rate for different molecules without any input from experiments nor adjustable parameters in their equations. The best results are obtained for **3**, **6**, **7**, **8**, **9**, and **12** where the deviation between experiments and simulations is vanishingly small. In particular, the comparison between **8** and **9** is illustrative of the power of *ab initio* simulations, which are shown to be able to predict differences in relaxation times coming from substituting a Cl^–^ with a Br^–^ as ligands in the first coordination sphere. Interestingly, in the case of Co^2+^ compounds with large *U*_eff_, such as for **6**, **4**, and **3**, we predict values of τ that fall within the same order of magnitude. Although simulations are not able to perfectly distinguish different Co^2+^ molecules to this degree of accuracy, the relaxation time is in good agreement with experimental observations. It is important to note that the contribution to relaxation coming from dipolar-mediated cross-relaxation, not included in simulations, is an important factor that potentially contributes to the residual deviations between simulations and experiments. Experimental results for diluted compounds or in external field are often not available and indeed all compounds, with exception of **5** and **11**, were measured in the absence of an external field (see Table S6 for more details on experimental conditions). In such cases, the effect of dipolar relaxation is particularly visible in **2**, where experimental relaxation times start flattening out at low temperatures. The importance of accounting for dipolar cross-relaxation in the comparison between relaxation data and simulations has been recognized for *S* = 1/2 systems^12^ and supports the hypothesis that the residual errors for Co^2+^ SMMs are due to this effect. This argument is also in agreement with the higher accuracy obtained in high-*U*_eff_ Dy SMMs, which are naturally screened from dipolar relaxation. Indeed, in the presence of a large anisotropy with virtually no rhombic terms, the system’s spin states are almost pure *Ĵ*_*z*_ eigenfunctions and a dipolar perturbation cannot induce a transition among *M*_*J*_ = ±15/2 Kramers states. The largest deviations are observed for **5** and **11**. These compounds have the smallest zero-field splittings and we attribute these somewhat larger errors to the absence of acoustic and border-zone phonons in our simulations. The latter are not accounted for by simulating the sole unit-cell phonons and their absence mostly affects the low-energy vibrational density of states in resonance with the spin transitions of **5** and **11**. Finally, we note that the simulation of relaxation times in Dy SMMs is particularly sensitive to the accuracy of the crystal field parameters appearing in *Ĥ*_CF_ and thus to the quality of the modelization of the magnetic ion’s coordination sphere. Geometries optimized with pDFT and the use of the first coordination sphere for the simulation of *Ĥ*_CF_ were found to be accurate in all cases except for **7**, where the Dy–H_2_O distances are not well reproduced by DFT and the inclusion of the second coordination sphere is necessary. Accurate results were obtained by simulating *Ĥ*_CF_ for a model including first and second coordination spheres with experimental X-ray distances (see Figure S6).

### Analysis of Spin Relaxation

**Figure 3:**
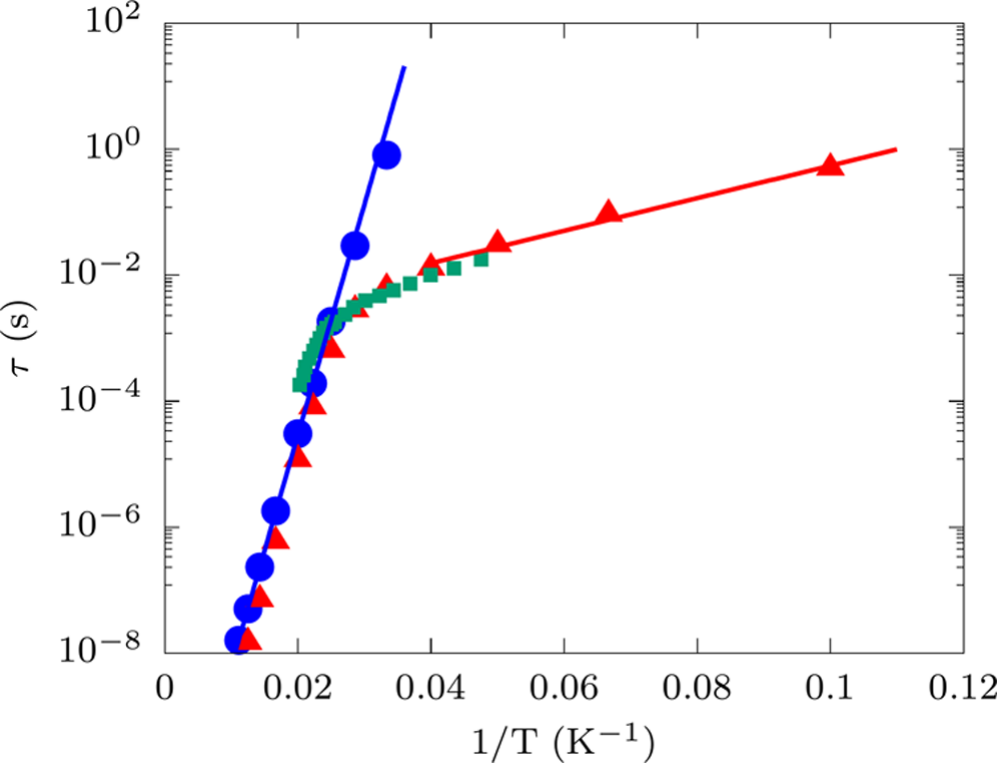
Orbach and Raman contributions to relaxation time. Comparison of experiment (green square) with the simulated Orbach (blue circle) and Raman (red triangle up) relaxation for **9**. The blue solid line represents the fitting of Orbach simulation data with the equation: τ_Orbach_ = τ_0_ exp(*U*_eff_/*k*_B_*T*). Similarly, the solid red line represents the fitting of Raman simulation data with the equation: τ_Raman_ = τ_0_^′^ exp(*W*_eff_/*k*_B_*T*).

Now that we have validated our simulations against experimental results, we are in the position to exploit the full power of *ab initio* spin dynamics to disentangle all of the contributions to relaxation time. Figure 3 shows the decomposition of τ in terms of Orbach and Raman contributions for compound **9**, as an illustrative example. The results for all of the 12 molecules are reported in Figure S1. In agreement with previous simulations,^12,24,26^ Orbach relaxation is found to dominate at high temperatures, while the Raman mechanism only becomes relevant at low temperatures, where phonons in resonance with high-energy spin transitions become too unpopulated. Most importantly, simulations reveal that experimental results for all of the molecules were obtained in a regime strongly influenced by Raman relaxation. The only exceptions are represented by **8** and **9**, where both magnetization decay experiments and AC magnetometry were employed to sample both the particularly long Orbach relaxation times and the Raman relaxation ones. Surprisingly, even in the case of **11**, Raman relaxation is found to be the dominant relaxation mechanism although the first excited KD is low in energy and amenable to promoting Orbach relaxation at low temperatures. **5** is the only system where the Orbach relaxation mechanism is found to be dominating in the experimentally accessible temperature range. In most cases, experimental relaxation times measured with AC magnetometry fall at the transition stage between Orbach and Raman-lead regimes, calling for a reinterpretation of the values of the extracted *U*_eff_ and τ_0_, and for new strategies for determining them in experiments. *Ab initio* simulations offer such an opportunity.

The effective reversal barrier and the preexponential factor, *U*_eff_ and τ_0_, have been extracted from the fitting of the simulated Orbach data with the Arrhenius expression of eq 1, as depicted in Figure 3. The results obtained for Raman relaxation follow a more complex mathematical law. Recent literature^24−26^ has shown that Raman relaxation is supposed to follow the temperature law
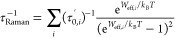
10where *W*_eff,*i*_ corresponds to the energy of the *i*th pair of degenerate phonons absorbed and emitted. If only a single pair of phonons contributes to relaxation, the Raman relaxation time also exhibits an Arrhenius behavior at low temperatures (*W*_eff,*i*_ ≫ *k*_B_*T*)
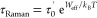
11This behavior is observed in simulations to a good degree, and we therefore attempt to fit the values of τ_0_^′^ and *W*_eff_ from low-*T* simulated data, as depicted in Figure 3.

Comparing eqs 1 and 11 with the expression of 1-phonon and 2-phonon relaxation rate, namely, eqs 5 and 7, it becomes clear that τ_0_ and τ_0_^′^ correspond to the inverse of the terms multiplying the functions *G*^1/2-ph^. The latter functions instead introduce the exponential *T*-behavior. The preexponential factors are therefore a measure of the coupling strength between the spin and the phonons responsible for relaxation. Using a simple probabilistic interpretation of this process, the *T* dependence of τ is a measure of the number of phonons available, while the inverse of the preexponential factors represents the efficiency of each spin-phonon interaction in triggering a transition.

**Figure 4:**
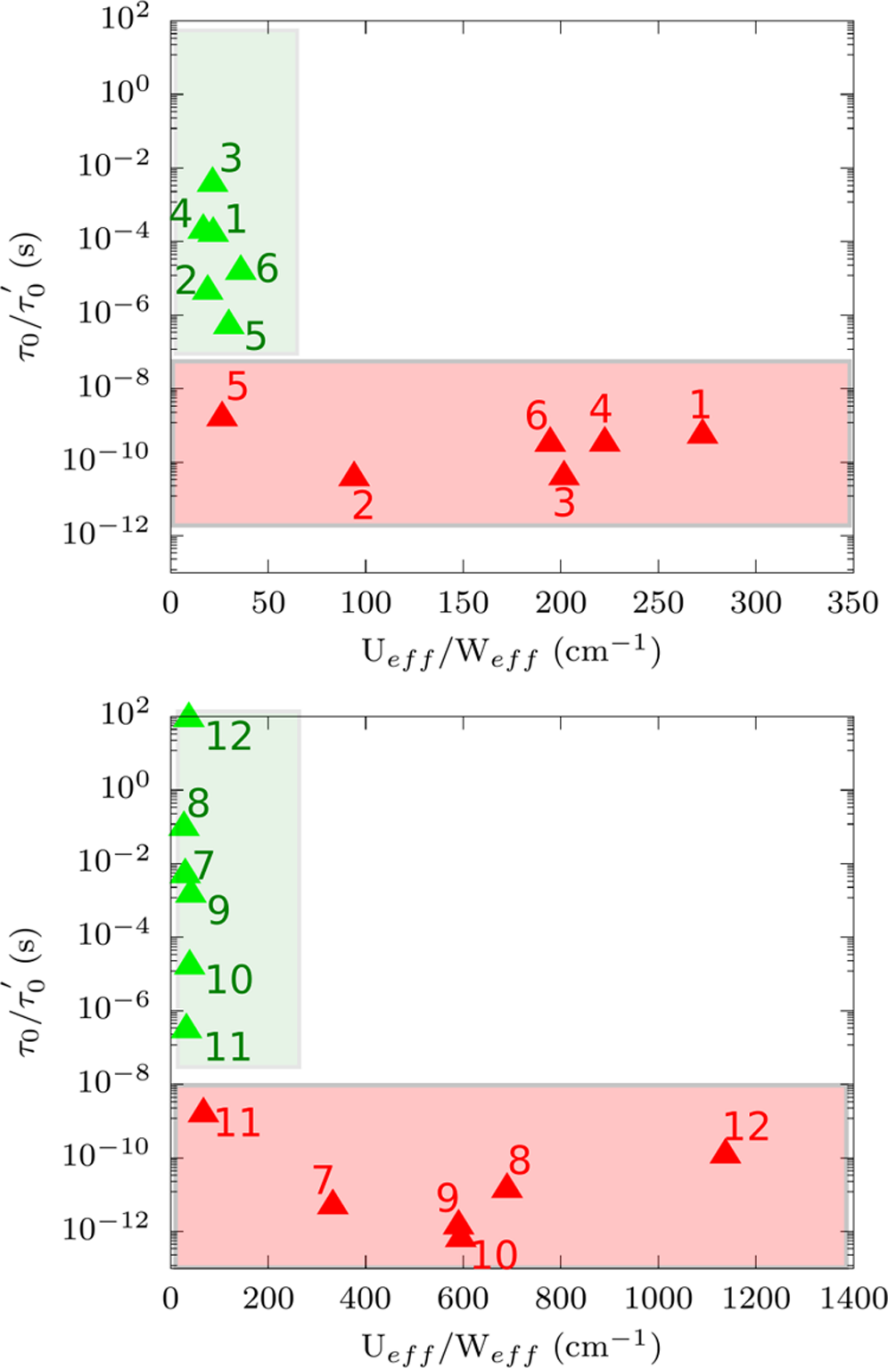
Interpretation of spin relaxation with Arrhenius laws. *W*_eff_ as a function of τ_0_^′^ and *U*_eff_ as a function of τ_0_ for complexes **1**–**6** (top) and for complexes **7**–**12** (bottom). Green and red triangles represent the simulated values for Orbach and Raman values, respectively. The selected compounds **1**–**12** are labeled with the corresponding index number.

Figure 4 reports the values of τ_0_, τ_0_^′^, *U*_eff_, and *W*_eff_, for all 12 compounds. The *U*_eff_ for the Co^2+^ systems (**1**–**6**) match the energy of the excited KD with *M*_s_ = ±1/2. This is in agreement with the fact that the latter is the only available excited KD able to mediate Orbach relaxation. The same is not true for Dy^3+^ complexes, where more than one excited KD is available. Orbach relaxation for the molecules **7**, **8**, **9**, and **11** is found to be mediated by transitions between ground state (*M*_*j*_ = ±15/2) and the second excited KD, while for **10** and **12**, Orbach transitions occur through the third and fifth excited KD, respectively. Importantly, the values of τ_0_ are found to lie in the range 10^–8^–10^–12^ s, a much smaller time window with respect to what is extracted from the literature (see Figure 1). Raman relaxation provides an opposite picture. *W*_eff_ is found to span a quite narrow range of small values in the order of tens of cm^–1^. On the other hand, the values of τ_0_^′^ span a range of *ca.* 5–6 and 10 orders of magnitude for Co^2+^ and Dy^3+^, respectively. The comparison between Figures 1 and 4 makes it clear that the experimental values of *U*_eff_ and τ_0_ extracted from the literature do not describe the sole Orbach relaxation mechanism but are deeply affected by Raman relaxation. This is particularly important for molecules with a reported small value of *U*_eff_.

The values of *U*_eff_ compare nicely with the energy of the excited KDs and present no mystery, but more insights on the other quantities are necessary to understand what regulates them. Let us begin from τ_0_. According to eq 5, τ_0_^–1^ receives contributions from three factors: (i) the density of phonons in resonance with the relevant spin transition; (ii) their coupling with the magnetic moment, as measured by the derivatives of the effective Hamiltonian coefficients (see eqs 3 and 4); and (iii) the nature of the static effective Hamiltonian used to compute the matrix elements of the spin-phonon coupling operator. Contributions (i) and (ii) can be easily assessed by computing the spin-phonon coupling density,^32^*i.e.*, the average coupling of magnetic moment to phonons with a certain energy *ℏ*ω_α_. This quantity is defined in the SI and reported in Figures S4 and S5 for all of the compounds. Despite the presence of different features among the 12 compounds, the average values of spin-phonon coupling intensity are not dramatically different. This suggest that contribution (iii) is in fact largely responsible for the variance of τ_0_.

**Figure 5:**
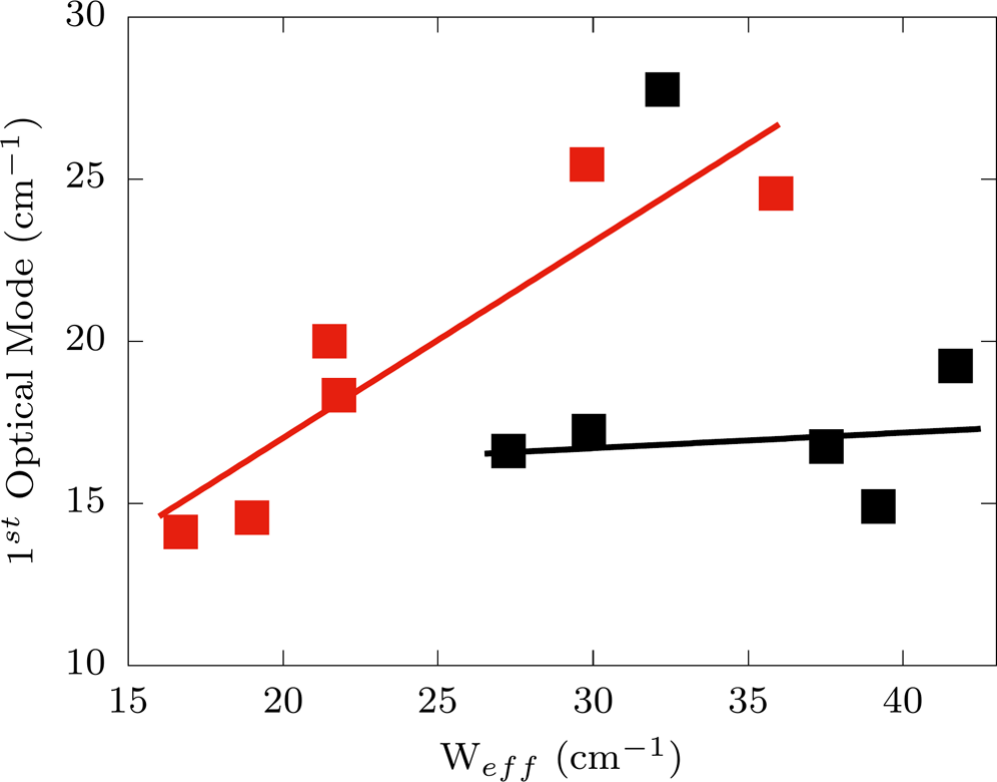
Correlation between phonon energies and Raman Arrhenius activation energy. *W*_eff_ is plotted as a function of frequency of the first optical mode at the Γ-point for complexes **1**–**6** (red squares) and **7**–**12** (black squares). The correlation coefficient for the two quantities is 0.91 and −0.14 for Co^2+^ and Dy^3+^, respectively.

Let us now turn to the analysis of Raman relaxation rates. According to recent literature, the value of *W*_eff_ should coincide with the lowest-energy phonons significantly coupled to the magnetic moment.^24−26^ In our approximation, this generally corresponds to some of the first available optical phonons. Figure 5 shows the correlation between the first mode at the Γ-point and the fitted values of *W*_eff_. In the case of Co^2+^, a good correspondence between the two quantities is found, validating previous results and further suggesting the importance of low-energy optical vibrations. Surprisingly, the same degree of correlation is not observed for Dy^3+^ compounds, where *W*_eff_ is found to span slightly larger values of energy than that of the first optical mode. Moreover, the values extracted for *W*_eff_ do not clearly correspond to peaks in the spin-phonon coupling density. For Dy^3+^ compounds, several excited KDs are at play, and it is hard to find a simple rationale to this behavior. We advance the hypothesis that the presence of very high-energy KDs involved in the virtual state of Dy^3+^ compounds promotes the effect of phonons with higher energy than the first ones available at the Γ-point. It is still important to remark that the values of *W*_eff_ are still in the order of tens of cm^–1^, a value commensurate with low-energy optical vibrations and much lower than the energy of excited KDs. Further analysis shows that none of the phonons with energy higher than *ca.* 100–150 cm^–1^ contribute to spin relaxation (see Figures S7). This is in agreement with previous observations that the main factor in the determination of most important phonons is the Bose–Einstein population.^24,26^ The latter decreases exponentially as the energy of vibrations increases, thus leaving the lowest-energy available modes to drive spin relaxation.

From a qualitative point of view, Raman relaxation rate depends on similar quantities to the Orbach one, and τ_0_^′^ is influenced by both the spin-phonon coupling density and the nature of static effective Hamiltonian. As discussed for Orbach relaxation, the differences in the former quantity across the 12 molecules are not dramatic and cannot account for the large span of values predicted for τ_0_^′^. The variance in values of τ_0_^′^ must therefore come (i) the nature of the eigenstates of the static effective Hamiltonian used to compute the matrix elements of the spin-phonon coupling operator and (ii) the energy of the excited KDs appearing at the denominator of eq 7. In both cases, these quantities are intimately linked to the nature of the static crystal field and the intensity of the zero-field splitting.

**Figure 6:**
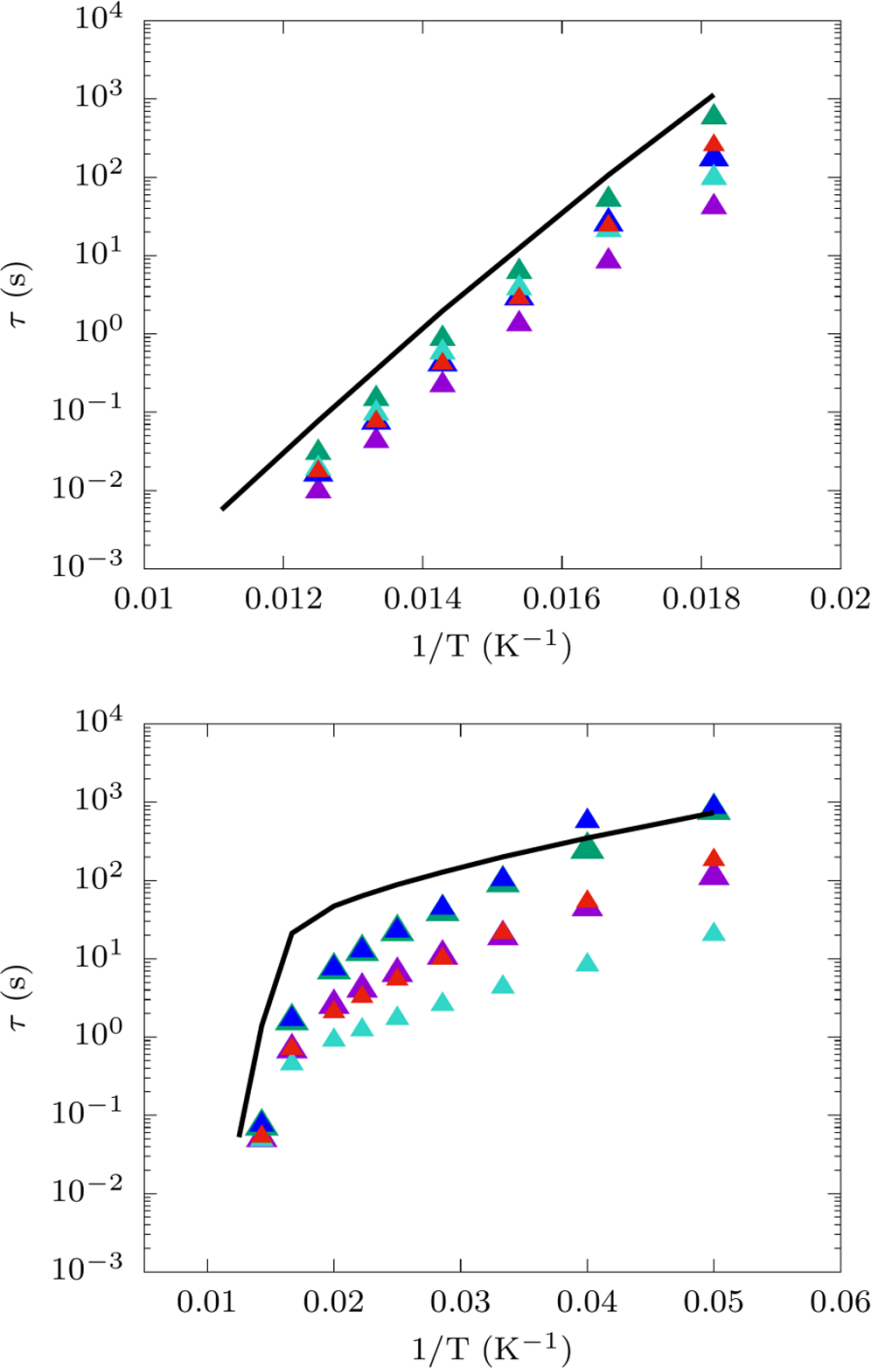
Orbach and Raman relaxation of compound **12** with artificial phonons and spin-phonon coupling coefficients. The black continuous line represents the original τ *vs* 1/*T* for **12**. Symbols correspond to the simulated values of τ obtained using the static effective Hamiltonian of **12** with phonons and spin-phonon coupling of other molecules: 7 (violet), 8 (green), 9 (blue), 10 (turquoise), 11 (red), and 12 (black).

According to this analysis, the static effective Hamiltonian stands out as the most important contribution to both τ_0_ and τ_0_^′^. To provide a conclusive proof of this claim, we perform a simulation of τ for Dy^3+^ compounds where the static effective crystal field of **12** is used together with the phonons and spin-phonon coupling of the other compounds. Results for Orbach and Raman relaxation in this artificial condition are reported in Figure 6 (top and bottom, respectively). Orbach rates are found to all fall within 1 order of magnitude, with the genuine value of **12** as the slowest one. In the case of Raman relaxation, a similar behavior is observed, except for a larger variance of relaxation times, which now span up to 2 orders of magnitude. The latter value should be compared with a total variation of ∼6 orders of magnitude in normal conditions (see, for instance, the values of τ at 20 K in Figure 2 (right)). We attribute this larger sensitivity to the details of spin-phonon coupling and the vibrational density of state in Raman relaxation to the fact that these quantities show a power law of four instead of two as in Orbach relaxation. Performing the same analysis for the Co^2+^ SMMs, we find the same qualitative behavior but a larger span of relaxation times, reaching almost 2 orders of magnitude for Orbach rates and 4 orders of magnitude for Raman relaxation (see Figure S3). This analysis conclusively demonstrates that the static effective Hamiltonian is the most important contribution to τ across all relaxation regimes. However, the details of spin-phonon coupling add on top of that to fine-tune the value of τ and in the case of Raman relaxation significantly contribute to determining the magnetic moment lifetime.

## Discussion and Conclusions

Molecular magnetic anisotropy has been identified as a key ingredient for slow spin relaxation since the very first observation of magnetic hysteresis in a mixed-valence Mn_12_ cluster, now 30 years ago.^5^ Decades of success stories of molecular magnetism have marked the synthesis of compounds with previously unimaginably large zero-field splitting values, reaching record values of above 2000 K.^15,17^ These results effectively translated into the possibility of stabilizing the molecular magnetic moment over times scales of hundreds of seconds at temperatures as high as 80 K.^17^ Despite the past success, the field of single-molecule magnets now finds itself at a critical stage. It has been argued that the strategies that have led to large *U*_eff_ in single-ion complexes cannot realistically lead to much further improvement and that new approaches to increasing τ must be found.^26,48^ We argue that further progress in the design of molecular compounds with long spin lifetime is to be found in a better understanding of the entire process of spin relaxation. In this study, we have highlighted how the unique focus on *U*_eff_ has led to overlooking many important aspects of spin relaxation. The analysis of the preexponential factor τ_0_ is a striking example of such a situation, where years of information available in the literature have remained unexplored.

To move out of this impasse, new tools are needed. In this work, we built on our recent contributions and have further shown how *ab initio* spin dynamics simulations provide an effective way to obtain unprecedented quantitative details on the spin relaxation process of single-molecules magnets. Here, we have fully disentangled the various contributions to both Orbach and Raman relaxation posing an end to years of debate on the subject. We have demonstrated that differently from the predictions of the canonical theory of spin-phonon relaxation^49^ and from what arise from the literature, there is no simple correlation between *U*_eff_ and τ_0_. Moreover, simulations made it possible to individuate realistic ranges for both these quantities and those relative to Raman relaxation, when interpreted as an Arrhenius process. We anticipate that this information will play a fundamental role in the interpretation of future experiments and will provide a guide to the assignment of Orbach and Raman relaxation mechanisms.

The unprecedented effort of studying 12 crystals of SMMs made it possible to take a first glimpse to the correlation between chemical structure and spin relaxation. Despite the large differences between the selected molecular compounds, we demonstrated that Orbach spin relaxation rate is mostly determined by the zero-field splitting. Now that spin relaxation can be correctly interpreted, it becomes clear that the natural variations of τ_0_ are too small to overcome the effect of *U*_eff_. The little dependence of the Orbach rate over the details of spin-phonon coupling suggests that little improvement can be achieved by serendipitously tuning vibrational modes. As highlighted in Figure S2, the vibrational density of states below *ca.* 1500–1700 cm^–1^ is densely populated and only a very delicate tailoring can bring phonons and spin transitions completely out of resonance.^50^ Similarly, the static zero-field splitting is found to also strongly affect Raman relaxation rate. However, in this case, the features of spin-phonon coupling and low-energy vibrations significantly contribute to modulating relaxation time and may offer a way forward to further improvement. These findings thus shift the attention to Raman relaxation as the most feasible improvement that can be obtained in single-ion SMMs, and point to the necessity of better understanding how the low-energy vibrational structure of coordination compounds can be chemically engineered.

We postpone a complete study of this aspect to a future work, but we here provide some qualitative insights based on the first few normal modes for each Dy compound (see the Supporting Information), which falls in the energy range of *W*_eff_ and are responsible for Raman relaxation. In Figure 6(bottom), compound **10** is observed to relax sensibly faster than the other Dy compounds when the same zero-field splitting of **12** is used for all of them. This is in agreement with the results of a qualitative inspection of low-energy vibrations, which suggests that nonchelating ligands in **10** lead to a flexible coordination shell able to effectively couple with spin. The result is corroborated by a higher value of the spin-phonon coupling distribution function, reported in Figure S5. The coordination shells of **8**, **9**, and **12** instead appear much more rigid, and their Raman relaxation is accordingly found to be the slower, once the effect of the static crystal field is taken into account (see Figure 6). The visualization of low-energy normal modes also suggests the important role played by organic ligands with high-mobility groups. For instance, *tert*-butyl groups are free to rotate and appear to couple with the rotation and translation of the molecular units inside the crystal to provide low-energy vibrations able to affect the ion’s coordination sphere. As a consequence of the large delocalization of vibrations over the entire unit cell, we advance the hypothesis that similar considerations might also apply to counterions. The same analysis is less obvious for Co compounds, where the amplitude of the first coordination shell vibrations appear visually comparable across the series. A full quantitative analysis of the vibrational motifs leading to strong coupling will necessarily require the use of analytical tools such as those previously applied to disentangle intramolecular and intermolecular modes.^32^ The present work only dealt with single-ion coordination compounds, and no application of *ab initio* spin dynamics to polynuclear single-molecule magnets has yet been presented. Nonetheless, most of the present results should also apply to this other class of compounds, suggesting that rigid ligand bridges among magnetic ions could further support the effect of large exchange coupling in protecting the spin from relaxation.

We envision that a tighter synergy between simulations and experimental techniques holds the key to significant advances in this direction. For instance, inelastic neutron scattering^51^ and terahertz spectroscopy^52−56^ have already been used to provide unique insights into the low-energy part of the vibrational spectrum. Similarly, far-infrared magneto spectroscopy can be used to provide insights into the spin-phonon coupling of vibrations in close resonance to the spin transitions responsible for Orbach relaxation.^28,57−59^ Once these techniques are combined with *ab initio* simulations, a clear picture of how vibrations couple with spin is possible.

It should also be reminded that although Orbach and Raman relaxation determine the temperature dependence of spin lifetime, the central figure of merit in the quest toward zero-field single-ion magnets is the presence of magnetic hysteresis.^49^ The latter is also strongly influenced by a third *T*-independent relaxation mechanism often labeled as quantum tunneling of the magnetization and connected to the magnetic dipolar noise felt by the magnetic molecule. A comprehensive design strategy should account for this mechanism, representing an interesting future direction for *ab initio* simulations.^60^

Last but not least, we would like to stress out that the present findings were made possible by studying a large number of complexes on the same footing. We envision that overcoming the traditional approach of studying a single molecule at the time or homologous small series of compounds can lead to a much better understanding of structure–property relations across the chemical space. Moreover, we have here shown that accurate predictions down to 1 order of magnitude of τ are now possible for a large breadth of chemical compositions and zero-field splittings. This level of accuracy is already enough for enabling a blind exploration of the chemical space in search of new compounds. We envision that the further combination of *ab initio* spin dynamics with machine learning^24,61−64^ and high-throughput strategies^21,65^ may lead to a significant reduction in its computational cost, potentially leading to a paradigm shift in the way we design molecular compounds.^66^

In conclusion, we have provided a full *ab initio* description of spin relaxation in 12 single-molecule magnets based on Co^2+^ and Dy^3+^ ions that represent the extent of the chemical space explored so far in this field. Our simulations made it possible to resolve a conflicting interpretation of experimental results in terms of Orbach and Raman mechanism and to rationalize all of the main contributions to relaxation. We found that zero-field splitting is the main figure of merit determining spin relaxation, but the efficiency of Raman relaxation is also significantly influenced by the details of the low-energy vibrational spectrum. We anticipate that these results will significantly inform future synthetic strategies.

## Supporting Information Available

List of experimental relaxation data and their reference; computed crystal field parameters and *g*-tensors; simulated relaxation curves; phonon density of states; relaxation rates with artificial phonons; spin-phonon coupling densities; convergence tests for **7**; convergences of relaxation rate with respect to phonons’ energy cutoff; and X-ray and optimized molecular geometries (PDF)
Coordinate displacements of low energy phonons and force constants files (ZIP)
