# Supplementary material for: Unraveling the Contributions to Spin–Lattice Relaxation in Kramers Single-Molecule Magnets
Source: J Am Chem Soc. 2022 Dec 9;144(50):22965–75. doi: 10.1021/jacs.2c08876 (PMC9782788; doi:10.1021/jacs.2c08876)
Supplement: Supplementary file 1 — ja2c08876_si_001.pdf [file ja2c08876_si_001.pdf]

Supporting Information- Unravelling the contributions to spin-lattice relaxation in Kramers single-molecule magnets

Sourav Mondal and Alessandro Lunghi *

School of Physics, AMBER and CRANN Institute, Trinity College, Dublin 2, Ireland

*lunghia@tcd.ie

Table 1: Table S1: **Co & Dy SMM compounds**. The table reports U_{eff} and τ for the 56 Cobalt SMMs and 183 Dysprosium individuated in literature.

U_{eff} (cm^{-1})	τ (s)	REF	U_{eff} (cm^{-1})	τ (s)	REF
56.300000	6.0000e-10	[1]	25.920000	9.4000e-11	[2]
20.700000	1.2000e-06	[1]	39.400000	1.3000e-08	[3]
62.300000	8.7000e-11	[1]	21.270000	4.6500e-10	[4]
11.100000	3.6000e-06	[5]	22.670000	1.5000e-08	[4]
16.700000	5.1000e-07	[5]	27.800000	5.9800e-11	[2]
23.100000	2.5000e-07	[6]	25.800000	1.2000e-09	[7]
24.100000	2.3000e-07	[6]	24.300000	2.1000e-10	[7]
2.800000	7.4000e-02	[8]	20.800000	6.0000e-09	[7]
11.800000	5.9000e-06	[8]	21.100000	7.0000e-10	[9]
2.100000	1.0000e-01	[8]	317.000000	4.6000e-11	[10]
23.000000	4.0000e-06	[11]	21.100000	1.0000e-06	[9]
8.700000	8.0000e-06	[11]	19.100000	3.0000e-06	[9]
16.200000	4.0000e-07	[12]	33.900000	4.5000e-06	[13]
24.000000	2.0000e-10	[14]	75.800000	1.0000e-07	[15]
24.000000	1.9000e-09	[16]	7.900000	6.1000e-06	[17]
22.900000	3.7000e-10	[18]	14.500000	1.0000e-06	[19]
10.900000	8.9000e-07	[20]	230.000000	7.6000e-11	[21]
59.900000	1.4000e-09	[22]	191.000000	8.8700e-10	[23]
17.000000	1.5000e-06	[24]	122.000000	2.6500e-09	[23]
308.000000	8.9000e-10	[10]	29.200000	1.4000e-07	[25]
413.000000	1.1000e-10	[10]	16.400000	4.8000e-06	[26]
43.000000	8.4000e-10	[27]	21.000000	4.6000e-08	[28]
29.800000	1.8000e-10	[29]	21.000000	1.0000e-07	[29]
13.600000	5.8000e-05	[30]	20.000000	2.0000e-09	[28]
5.700000	4.6000e-05	[30]	48.000000	1.4000e-15	[31]
23.300000	7.4000e-06	[26]	450.000000	1.7900e-09	[32]
19.700000	5.6000e-06	[26]	87.000000	1.1000e-09	[33]
10.400000	5.6000e-06	[34]	36.000000	5.6000e-10	[27]

U_{eff} (cm^{-1})	τ (s)	REF	U_{eff} (cm^{-1})	τ (s)	REF
52.125000	4.2100e-05	[35]	712.375000	4.2100e-12	[36]
26.187600	1.4700e-09	[37]	492.060000	9.4600e-11	[36]
10.508400	2.9100e-07	[38]	377.385000	2.0000e-11	[39]
23.630000	2.5100e-06	[40]	328.040000	8.7000e-12	[39]
34.750000	6.8000e-07	[40]	56.295000	2.0000e-08	[41]
33.916000	3.9900e-06	[42]	47.121000	1.1900e-07	[43]
40.240500	3.1000e-06	[42]	13.552500	7.0000e-09	[44]
23.630000	2.0700e-05	[45]	15.693100	2.4300e-07	[46]
32.824850	6.0000e-09	[47]	36.237300	1.5000e-06	[48]
5.907500	1.6000e-07	[49]	50.318000	3.0000e-09	[48]
37.808000	8.7000e-06	[50]	17.340250	5.5000e-08	[48]
20.433000	5.1000e-08	[51]	29.885000	5.2000e-10	[52]
94.520000	8.0000e-08	[53]	25.715000	1.2000e-09	[52]
134.830000	4.7000e-08	[53]	20.155000	6.8000e-09	[52]
46.565000	1.6800e-07	[54]	70.195000	6.0000e-09	[55]
23.977500	1.1000e-06	[56]	70.195000	6.0000e-09	[55]
16.541000	9.1400e-05	[57]	41.005000	9.0000e-08	[55]
22.726500	1.8200e-06	[57]	29.885000	1.3700e-06	[58]
28.495000	9.9000e-07	[59]	43.785000	1.0200e-08	[58]
27.105000	4.1400e-06	[60]	47.260000	2.0700e-10	[58]
44.271500	1.3200e-06	[60]	9.521500	1.2000e-07	[61]
126.073000	8.8000e-10	[62]	66.511500	3.4000e-07	[61]
14.101550	5.0000e-06	[63]	52.820000	4.2000e-08	[61]
47.260000	7.0000e-08	[64]	150.120000	4.7000e-09	[65]
11.467500	4.0000e-05	[66]	11.815000	1.4600e-07	[67]
22.379000	4.5000e-05	[66]	34.027200	3.7400e-07	[68]
35.167000	5.4000e-06	[66]	42.033600	1.7300e-07	[68]
18.973500	5.2000e-05	[66]	38.030400	1.8600e-07	[68]
46.565000	1.6800e-07	[69]	452.445000	5.6300e-12	[70]
39.406500	3.7000e-07	[71]	41.005000	4.8000e-07	[72]
19.946500	2.5000e-07	[71]	18.070000	2.6000e-06	[72]
19.321000	1.0000e-06	[71]	27.591500	3.9000e-06	[73]
33.985500	8.5200e-11	[74]	71.459900	1.1900e-07	[75]
22.935000	5.5000e-10	[76]	44.174200	4.4800e-06	[75]
30.441000	3.3000e-06	[77]	57.685000	5.3000e-07	[78]
9.799500	7.1000e-06	[77]	27.105000	3.0900e-07	[78]
25.367500	2.0000e-06	[77]	38.225000	8.2300e-12	[78]
17.013600	4.0000e-07	[79]	13.900000	1.8000e-08	[80]
54.905000	2.9000e-08	[81]	27.800000	4.1000e-08	[80]
21.058500	4.5000e-06	[82]	20.016000	1.1000e-05	[83]
37.113000	1.6000e-12	[84]	29.051000	7.5000e-06	[83]
27.661000	1.6000e-07	[84]	28.981500	4.7000e-06	[83]
44.368800	5.7280e-06	[85]	22.518000	1.1000e-05	[83]
16.958000	2.6300e-08	[86]	29.565300	1.1300e-06	[87]
52.764400	6.8000e-11	[88]	40.310000	3.4000e-07	[89]

U_{eff} (cm^{-1})	τ (s)	REF	U_{eff} (cm^{-1})	τ (s)	REF
59.075000	3.7000e-07	[89]	20.155000	1.8000e-06	[90]
0.278000	9.9000e-06	[89]	39.406500	4.2000e-07	[91]
64.635000	1.1000e-06	[92]	3.162250	3.5500e-07	[93]
16.541000	2.4700e-06	[94]	262.988000	5.0000e-08	[95]
353.060000	8.6000e-12	[96]	270.007500	1.5000e-08	[95]
15.713950	9.7700e-07	[97]	42.721650	2.6600e-06	[98]
12.510000	6.0000e-06	[99]	53.883350	1.1400e-06	[98]
12.510000	2.6700e-08	[100]	1.744450	1.1200e-05	[98]
3.197000	3.6600e-05	[101]	29.259500	8.2900e-06	[98]
30.858000	3.2000e-07	[101]	929.910000	1.8500e-12	[102]
45.175000	2.6000e-08	[103]	852.070000	1.1500e-12	[102]
38.989500	1.2500e-08	[104]	764.500000	9.0500e-12	[102]
1223.200000	1.9860e-11	[105]	386.420000	9.3300e-12	[106]
14.824350	3.1700e-07	[107]	41.978000	4.4000e-11	[108]
39.128500	1.1000e-07	[109]	28.147500	5.2000e-08	[110]
15.985000	1.3000e-06	[111]	38.920000	9.3000e-07	[112]
55.530500	4.1300e-06	[113]	144.928350	8.5000e-09	[114]
77.701000	5.6500e-07	[113]	169.267250	3.0100e-08	[114]
116.899000	6.5900e-07	[113]	15.915500	2.9000e-06	[115]
16.888500	3.4000e-09	[116]	106.960500	2.5000e-08	[115]
17.653000	5.8000e-07	[117]	23.630000	3.6000e-07	[118]
319.700000	2.0000e-11	[119]	13.205000	3.8000e-07	[118]
417.000000	1.2000e-11	[119]	6.644200	9.5400e-06	[120]
322.480000	1.8300e-09	[121]	52.797760	7.5500e-06	[122]
22.865500	1.2400e-05	[123]	53.515000	5.0700e-06	[124]
45.175000	1.2900e-11	[125]	43.090000	1.0000e-07	[126]
26.966000	2.5000e-06	[127]	1223.200000	2.0000e-12	[128]
64.496000	3.7900e-07	[129]	22.240000	1.3000e-07	[130]
62.550000	1.3600e-07	[129]	26.201500	6.1000e-08	[130]
53.167500	1.1000e-07	[131]	32.602450	1.3700e-07	[132]
38.225000	8.3000e-07	[131]	124.405000	1.9900e-07	[133]
28.356000	7.0000e-07	[131]	44.480000	6.2000e-07	[133]
18.070000	2.6000e-05	[134]	25.020000	4.4000e-06	[135]
13.205000	3.4000e-05	[134]	9.313000	3.9000e-07	[3]
14.796550	1.5400e-08	[136]	31.066500	1.1000e-08	[137]
117.455000	1.8800e-08	[136]	65.121500	2.8800e-10	[138]
39.823500	1.9200e-06	[139]	34.750000	6.8000e-07	[140]
55.669500	3.0500e-07	[139]	17.375000	6.0000e-06	[141]
59.978500	1.4000e-07	[142]	11.954000	9.5000e-06	[143]
75.755000	1.2000e-07	[142]	21.718750	2.3900e-08	[144]
18.556500	9.5000e-07	[145]	26.743600	1.1200e-06	[146]
35.584000	5.8900e-06	[147]	9.007200	2.7000e-06	[148]
23.630000	3.2000e-06	[149]	11.022700	9.2600e-06	[150]
502.485000	2.3600e-10	[151]	7.645000	2.2000e-05	[152]
807.590000	1.0200e-12	[151]	16.012800	4.2000e-07	[94]
0.278000	9.9000e-06	[153]	70.195000	5.1000e-10	[154]
1223.200000	1.9860e-11	[105]			

TABLE S2: **Crystal Field Parameters** Crystal field parameter with rank of l = 2, 4, and 6 are reported for **7**, **8**, and **9**.

l	m	**7**	**8**	**9**
2	-2	0.0116382160	-0.0826793069	0.0549505597
2	-1	3.4257015712	-0.1527753972	-0.0838634963
2	0	-5.8586792439	-8.7796677967	-7.6110790511
2	1	-0.0099168347	6.1522171892	5.8584518358
2	2	0.9673412592	-0.5972375274	0.5796986733
4	-4	0.0027628929	-0.0006242182	-0.0000287162
4	-3	-0.0106387059	-0.0015804297	0.0000880570
4	-2	0.0048945377	0.0013029424	0.0009347407
4	-1	0.1072086362	0.0001074779	-0.0003398877
4	0	-0.1846407837	-0.0445629700	-0.0376264303
4	1	0.0001298547	0.1522592206	0.1440703047
4	2	-0.0011175341	-0.0810106000	-0.0856459975
4	3	-0.0062517828	-0.0047288351	-0.0102090366
4	4	0.0089904206	-0.0006351176	-0.0101781822
6	-6	-0.0004116294	0.0000051326	-0.0000052892
6	-5	-0.0001129912	0.0000270237	0.0000054361
6	-4	0.0003933766	0.0000084263	-0.0000062021
6	-3	0.0001579314	0.0000078332	0.0000089929
6	-2	0.0000512990	-0.0000113149	0.0000015686
6	-1	-0.0019701136	-0.0000029264	-0.0000011981
6	0	0.0011167029	-0.0007246044	-0.0007860787
6	1	-0.0000500032	-0.0004747567	-0.0003637745
6	2	-0.0005854917	0.0000051248	-0.0002102721
6	3	0.0001137287	0.0003188668	0.0004103597
6	4	-0.0006425285	0.0005579449	0.0005183958
6	5	-0.0000810403	0.0006447734	0.0007425981
6	6	0.0005401116	0.0010857455	0.0010163157

TABLE S3: **Crystal Field Parameters** Crystal field parameter with rank of l = 2, 4, and 6 are reported for **10**, **11**, and **12**.

l	m	**10**	**11**	**12**
2	-2	-2.3065713232	0.3577254846	11.0687564278
2	-1	-5.3072539097	0.2173623886	0.4363723381
2	0	-1.9292231809	-7.8353612716	11.1144759237
2	1	-9.8318549040	0.1053194000	-0.8325478201
2	2	-0.7329379913	-0.1675615285	-13.5092124727
4	-4	0.0628592935	0.0013903411	0.0369873249
4	-3	-0.0764898133	0.0071693547	0.0005814264
4	-2	-0.0063222450	0.0010805203	0.0006340423
4	-1	0.0452744858	-0.0001020090	-0.0001981386
4	0	-0.0323086070	0.1078582823	-0.0156835041
4	1	-0.0756484466	-0.0002993690	0.0017361378
4	2	-0.0004469365	-0.0017197848	0.0101241854
4	3	0.0442063120	0.0006781750	-0.0010780319
4	4	0.0624170051	0.0008755701	-0.0033934035
6	-6	-0.0000185657	0.0009055887	0.0017818310
6	-5	0.0004616557	0.0000327369	0.0000588971
6	-4	0.0009230402	-0.0000029653	0.0001654676
6	-3	-0.0000185859	0.0001999129	-0.0000096911
6	-2	0.0002903747	0.0000419533	0.0001474241
6	-1	0.0000174419	0.0000304756	0.0000178138
6	0	-0.0007014251	-0.0002546643	0.0000674272
6	1	0.0000595712	0.0000235277	-0.0000208904
6	2	-0.0004374130	0.0000336993	0.0001148088
6	3	-0.0003279038	0.0002739388	-0.0000414967
6	4	0.0003567190	-0.0000416915	-0.0000176056
6	5	0.0005184329	-0.0000170437	0.0000504922
6	6	-0.0008630159	-0.0008856957	0.0003649024

TABLE S4: **Dy SMMs Kramer's Doublets Energies.** Computed energy in cm^{-1} of the KDs for the complexes 7–12

	KD1	KD2	KD3	KD4	KD5	KD6	KD7	KD8
**7**	0.00	293.84	437.94	498.52	523.02	582.70	638.90	706.85
**8**	0.00	394.41	636.50	741.33	768.63	783.44	812.12	864.21
**9**	0.00	370.51	573.19	640.32	702.97	718.00	728.96	810.70
**10**	0.00	240.94	452.52	573.31	620.83	704.76	761.92	968.38
**11**	0.00	10.55	68.74	166.01	274.85	393.68	472.93	520.95
**12**	0.00	451.00	752.26	975.82	1159.41	1308.57	1413.75	1473.01

TABLE S5: **Dy SMMs g tensors.** g tensors corresponding to each KD for the complexes 7–12

		**7**	**8**	**9**	**10**	**11**	**12**
KD1	g_x	0.000018	0.000228	0.001008	0.003047	0.030002	0.000002
	g_y	0.000113	0.000283	0.001364	0.004857	0.053263	0.000003
	g_z	19.861855	19.865906	19.860118	19.636431	19.527463	19.579332
KD2	g_x	0.054184	0.052223	0.107678	0.036654	0.020140	0.000011
	g_y	0.057415	0.059450	0.151784	0.041708	0.064976	0.000016
	g_z	17.054415	16.987918	16.919610	16.900190	18.408833	16.774470
KD3	g_x	0.661522	0.267119	2.086339	0.207555	0.044689	0.001914
	g_y	3.344376	0.569134	4.869881	0.255673	0.082302	0.002125
	g_z	16.019078	13.919003	11.726564	14.588700	14.549004	14.134042
KD4	g_x	3.414824	5.368526	1.830876	0.988891	0.253496	0.002284
	g_y	3.776073	6.427398	6.698137	1.534766	0.284410	0.005746
	g_z	7.933602	6.983514	8.539995	14.077576	11.751497	11.501928
KD5	g_x	0.280516	0.842745	0.730344	2.920243	4.015049	0.107421
	g_y	1.170205	1.670309	1.372234	5.431001	4.483045	0.112281
	g_z	12.557556	16.684468	12.244360	9.773901	8.364658	8.900419
KD6	g_x	1.240577	0.561377	0.272030	1.903553	3.256554	0.174136
	g_y	4.232480	4.835739	0.324692	3.874651	5.255842	0.384298
	g_z	8.486545	11.519193	18.375360	10.662750	5.679092	6.326653
KD7	g_x	1.484707	0.691294	1.295311	0.938560	2.734825	3.638125
	g_y	2.233398	3.276402	1.893175	2.582974	3.604480	3.658512
	g_z	4.397454	9.927467	12.870477	15.823137	4.377979	4.033277
KD8	g_x	0.520194	0.397944	0.034986	0.006481	1.131326	1.128157
	g_y	1.252197	1.185749	0.067722	0.008248	6.846465	6.405268
	g_z	16.474694	17.046018	18.600018	19.733254	13.982897	14.117889

TABLE S6: **Dilution data and value of magnetic field used in the Experiment**

System	Concentration (in %)	Magnetic Field (Oe)
**1**	100.0	0
**2**	100.0	0
**3**	100.0	0
**4**	100.0	0
**5**	100.0	1000
**6**	100.0	0
**7**	100.0	0
**8**	100.0	0
**9**	100.0	0
**10**	100.0	0
**11**	100.0	1500
**12**	100.0	0

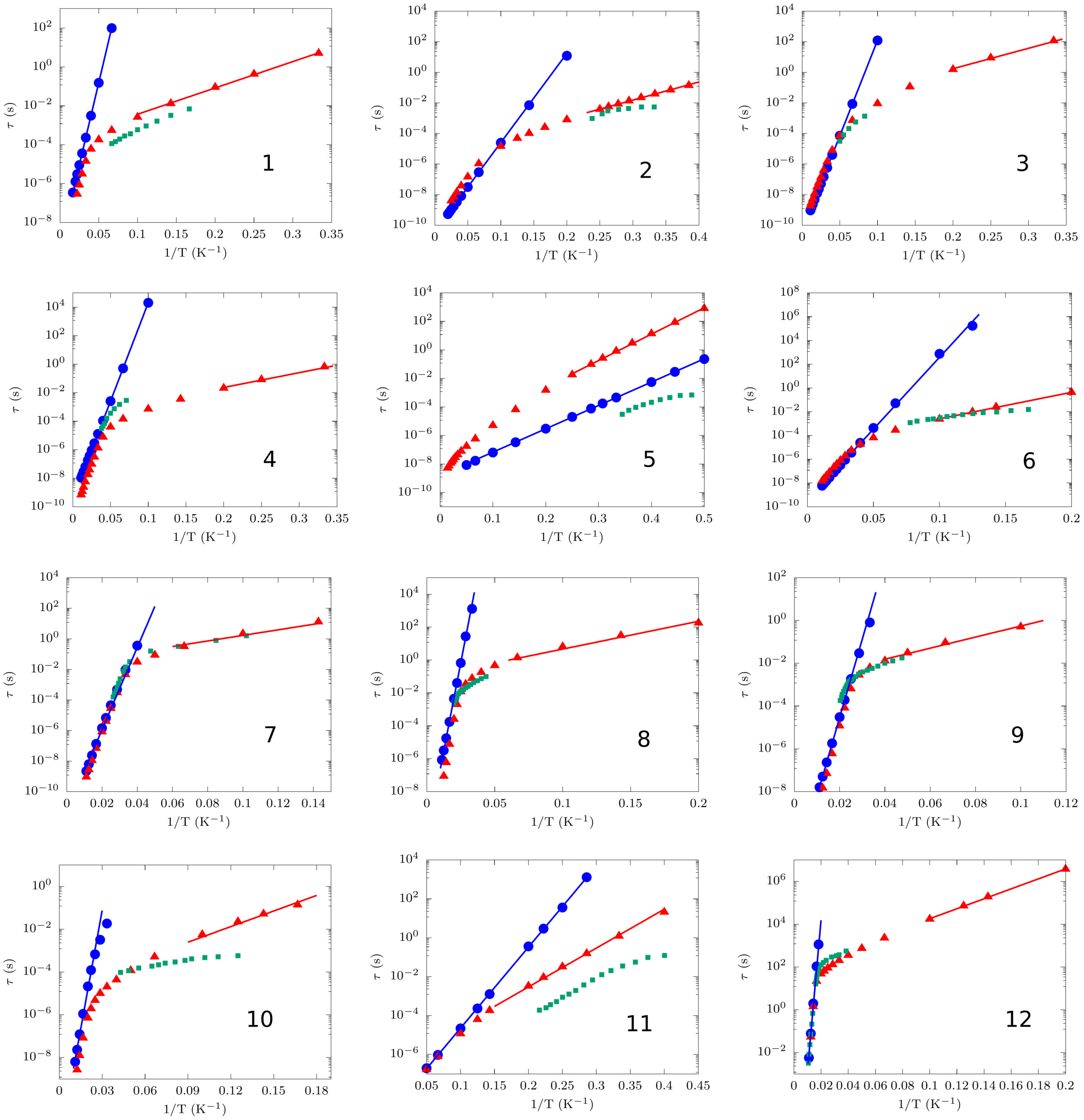

FIG. S1: **Orbach and Raman contributions to relaxation time** Comparison of experiment (green square) with the simulated Orbach (blue circle) and Raman (red triangle up) relaxation for **1-12**. Blue solid line represents the fitting of Orbach simulation data with the equation: $\tau_{Orbach} = \tau_0 \exp(U_{eff}/k_B T)$. Similarly, solid red line represents the fitting of Raman simulation data with the equation: $\tau_{Raman} = \tau_0^{'} \exp(W_{eff}/k_B T)$.

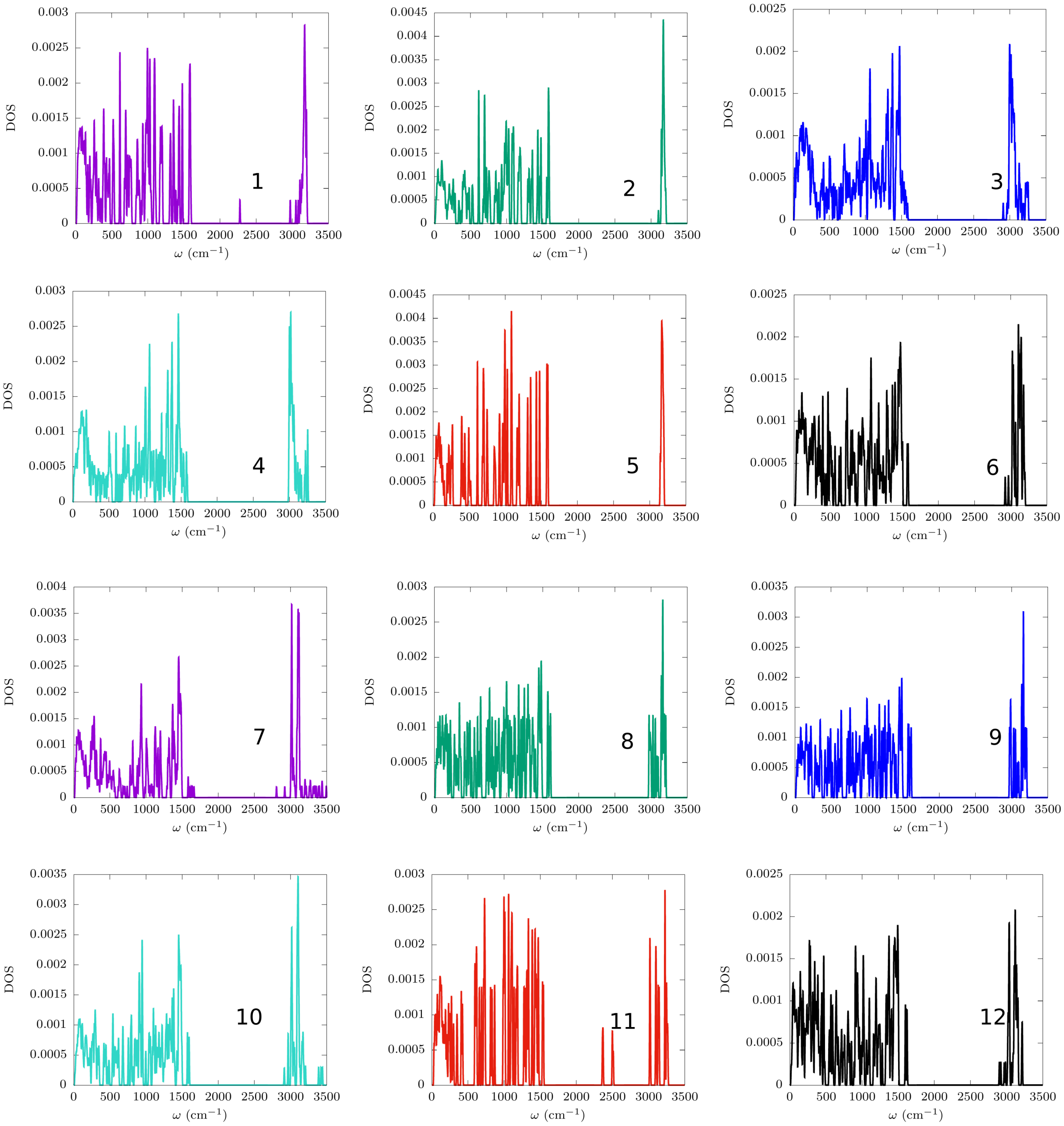

FIG. S2: **Phonon density of states for 1–12.** The total phonon density of states (DOS) as a function of the phonon frequency with Gaussian smearing of 5 cm^{-1}. Color code for top two panels: violet (**1**), green (**2**), blue (**3**), turquoise (**4**), red (**5**), and black (**6**). Color code for bottom two panels: violet (**7**), green (**8**), blue (**9**), turquoise (**10**), red (**11**), and black (**12**).

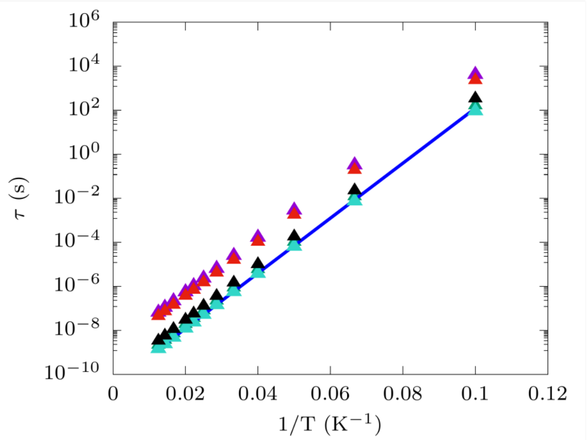

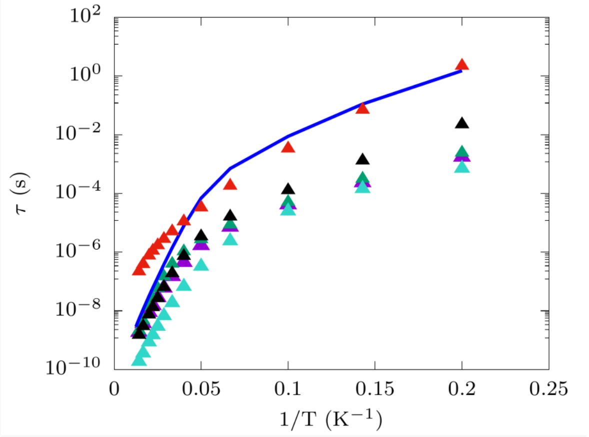

FIG. S3: **Relaxation of 3 with artificial phonons and spin-phonon coupling.** The blue continuous line represents the original τ vs $1/T$ for **3**. Orbach and Raman relaxation rates are reported in the left and right panel, respectively. Symbols corresponds to the simulated values of τ obtained using the static effective Hamiltonian of **3** with phonons and spin-phonon coupling of other molecules: **1** (violet), **2** (green) **4** (turquoise), **5** (red), and **6** (black).

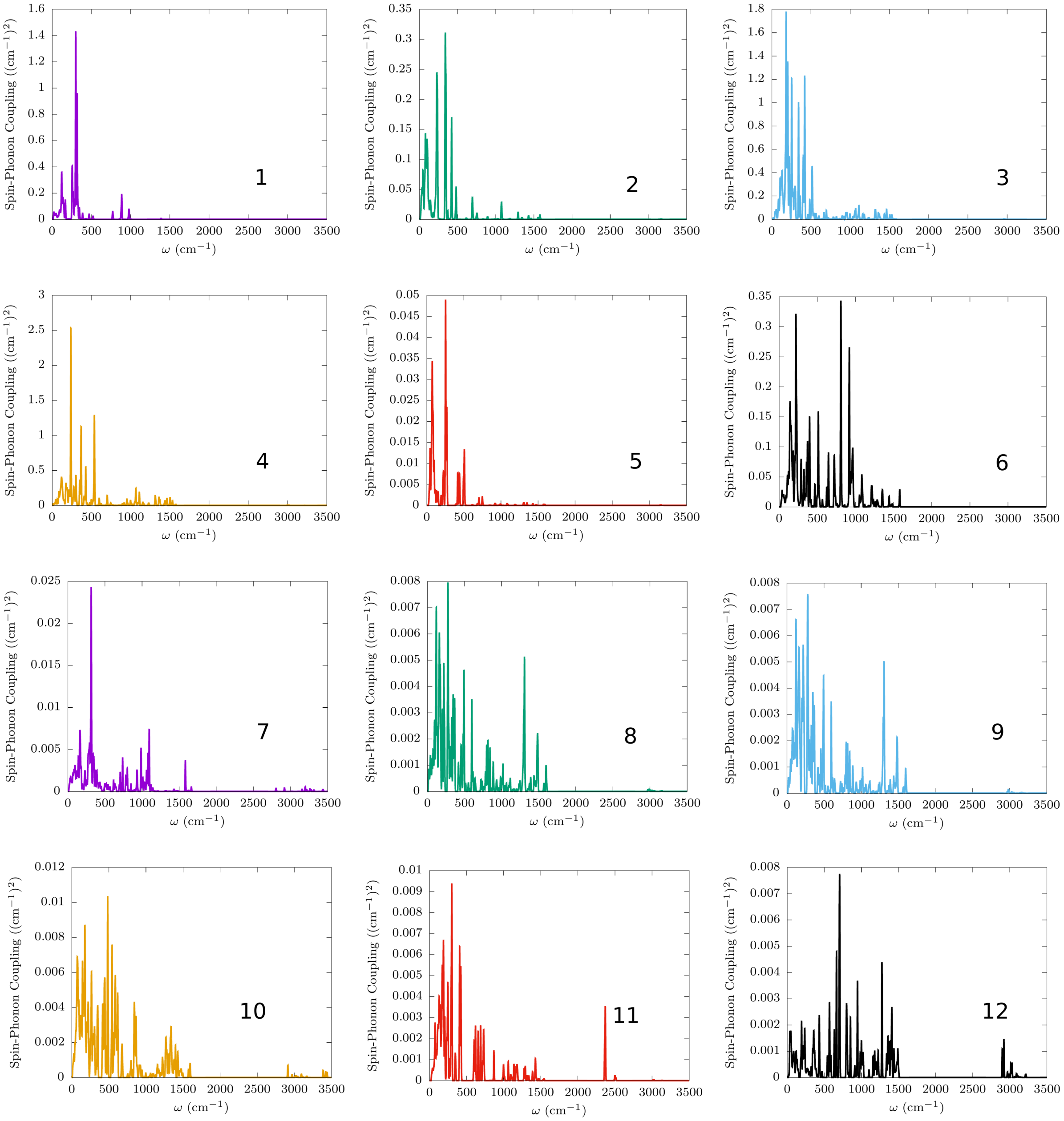

FIG. S4: **Spin-Phonon Coupling Density for 1–12.** The spin-phonon coupling density coupling density, $D(\omega)$, has been calculated as $D(\omega) = \sum_\alpha \sum_{ij} \left(\partial \hat{D}_{ij}/\partial Q_\alpha\right)^2 \delta(\omega - \omega_\alpha)$ and $D(\omega) = \sum_\alpha \sum_{lm} \left(\partial \hat{B}^l_m/\partial Q_\alpha\right)^2 \delta(\omega - \omega_\alpha)$ for Co^{2+} and Dy^{3+}, respectively. A Gaussian smearing of 5 cm^{-1} has been applied. Color code for top two panels: violet (**1**), green (**2**), blue (**3**), yellow (**4**), red (**5**), and black (**6**). Color code for bottom two panels: violet (**7**), green (**8**), blue (**9**), yellow (**10**), red (**11**), and black (**12**).

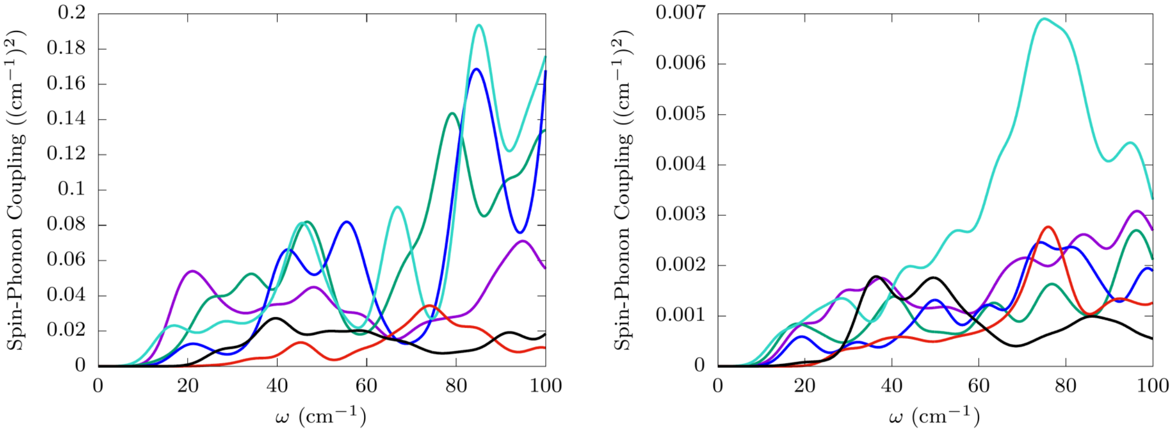

FIG. S5: **Comparison of Spin-Phonon Coupling Density for 1–6 (left) and 7-12 (right).** The spin-phonon coupling density coupling density, $D(\omega)$, has been calculated as $D(\omega) = \sum_\alpha \sum_{ij} \left(\partial \hat{D}_{ij}/\partial Q_\alpha\right)^2 \delta(\omega - \omega_\alpha)$ and $D(\omega) = \sum_\alpha \sum_{lm} \left(\partial \hat{B}^l_m/\partial Q_\alpha\right)^2 \delta(\omega - \omega_\alpha)$ for Co^{2+} and Dy^{3+}, respectively. A Gaussian smearing of 5 cm^{-1} has been applied. Color code for left panels: 1 (violet), 2 (green), 3 (blue), 4 (turquoise), 5 (red), and 6 (black). Color code for right panels: 7 (violet), 8 (green), 9 (blue), 10 (turquoise), 11 (red), and 12 (black).

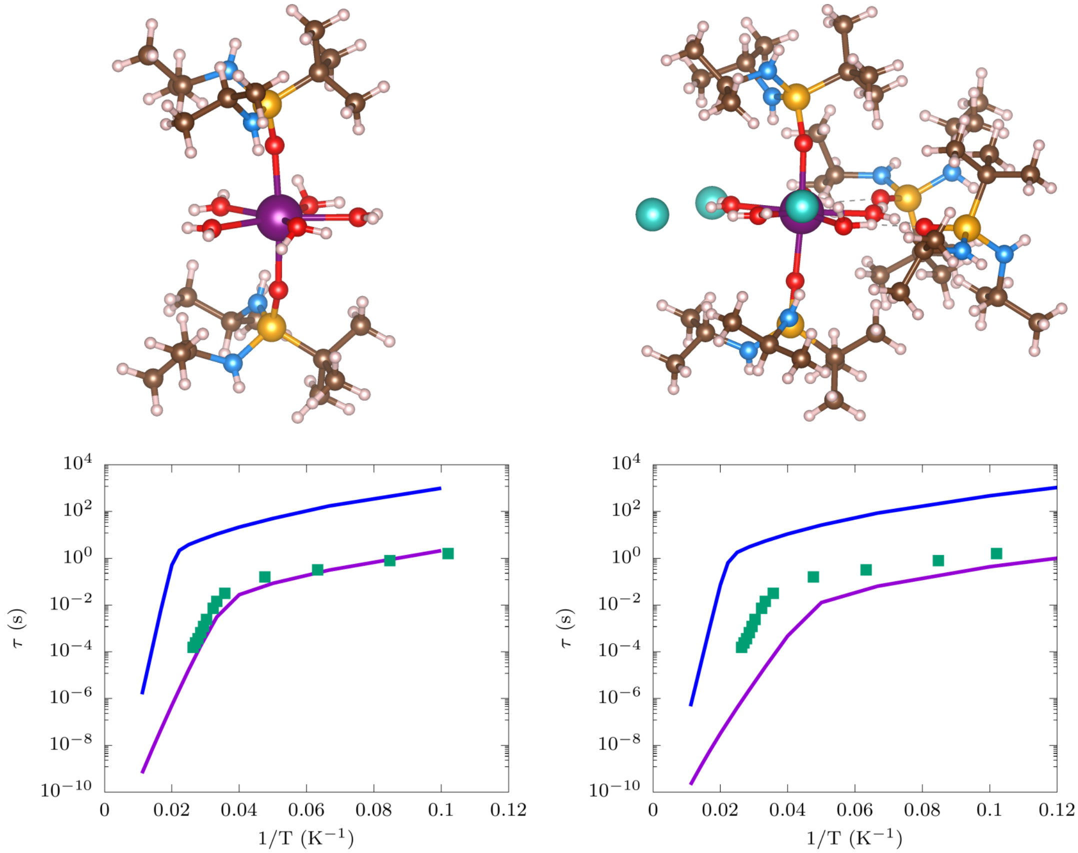

FIG. S6: **Simulated values of τ as a function of $1/T$ for the experimental X-ray coordinates (bottom left) and optimized coordinates (bottom right) of 7.** Blue line represents the simulation result for the model that includes the atoms inside the 1st coordination shell of the **7** (top left). Similarly, violet line represents simulation results of the model that includes atoms inside 2nd coordination sphere of the **7** (top right). Symbols corresponds to the experimental values. Color codes for the atoms: Dy in purple, N in light blue, O in red, I in turquoise, C in dark brown, P in gold, H in pale pink.

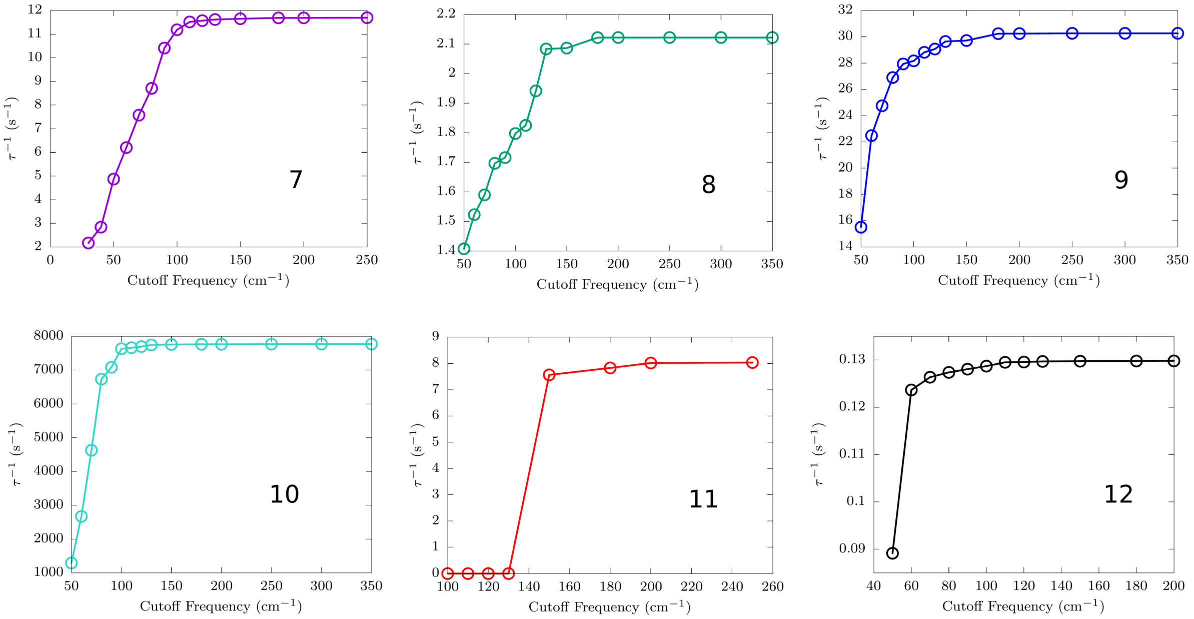

FIG. S7: **Relaxation time as function of phonon energy cutoff.** The Raman relaxation time τ for 1 (violet), 2 (green), 3 (blue), 4 (turquoise), 5 (red), and 6 (black) are computed at 20 K including phonons up to a cutoff value of energy

Molecule 1: Geometry from the X-ray measurement

Co	7.671805000	14.351643000	4.965991000
S	6.535126000	16.357733000	5.064850000
C	6.774911000	16.655566000	6.756802000
C	7.367962000	15.809731000	7.633639000
S	8.040423000	14.259183000	7.227371000
S	7.484788000	16.379885000	9.283468000
S	6.273205000	18.191909000	7.437468000
C	6.794458000	17.942169000	9.060648000
S	6.691364000	19.069802000	10.281686000
S	6.542223000	12.752702000	3.760599000
S	9.638461000	14.064731000	3.813896000
C	7.860517000	12.249014000	2.738826000
C	9.107544000	12.782995000	2.771492000
S	7.613105000	10.957387000	1.574352000
S	10.242711000	12.087199000	1.632120000
C	9.208538000	10.894819000	0.912942000
S	9.730210000	9.860547000	0.261676000

Molecule 1: Optimized Geometry

Co	6.7718512677	14.0741631181	4.9686234908
S	7.0716665439	13.9197226860	7.1873922879
S	5.6690434256	16.0409184201	5.1248128697
S	8.7191795419	13.8904667307	3.8412592129
S	5.7789959524	12.4520444174	3.7674072904
C	6.3604303268	15.4186186933	7.7175952248
C	5.7858511980	16.3199376661	6.8414446468
C	8.3218375222	12.5966952379	2.7435674849
C	7.0802102948	11.9900586435	2.7023125517
S	6.3843170621	15.9029295659	9.3939168041
S	5.2292332631	17.8099180273	7.5737986225
S	9.4947211556	11.9753947292	1.6087633859
S	6.8985596579	10.7323409813	1.4926429049
C	5.7040172890	17.4861746725	9.2187650552
C	8.5189620852	10.7613963224	0.8419083292
S	5.5658116468	18.5770595279	10.4795329204
S	9.0958576266	9.7733935081	-0.3760610616

Molecule 2: Geometry from the X-ray measurement

Co	1.506499	10.764206	0.414024
S	0.900702	12.969251	0.863740
S	-0.630822	9.850986	0.263627
S	2.753892	10.415544	-1.502746
S	3.027943	9.878196	1.923416
C	2.377310	13.975174	0.913216
C	-0.483109	8.032887	0.187074
C	2.008897	11.246042	-2.857063
C	2.286945	9.211500	3.375947
C	2.276518	15.365718	0.836418
C	3.649380	13.407744	1.065829
C	-1.603990	7.293178	0.071384
C	0.738565	7.384787	0.273226
C	0.792437	11.947467	-2.723896
C	2.615389	11.247410	-4.127935
C	3.044626	9.114422	4.556236
C	0.962741	8.709701	3.404254
H	1.444112	15.762235	0.713835
C	3.399137	16.154650	0.937831
C	4.758096	14.244532	1.186689
H	3.753648	12.483449	1.085522
H	-2.422493	7.725245	-0.019692
C	-1.574447	5.919042	0.086153
C	0.776797	5.988774	0.292919
H	1.527526	7.875648	0.319995
H	0.359725	11.969344	-1.900278
C	0.238079	12.605139	-3.809909
C	2.036702	11.896877	-5.208288
H	3.423466	10.801670	-4.246087
H	3.906574	9.464451	4.578390
C	2.530237	8.503239	5.693449
C	0.479633	8.125864	4.519314
H	0.424023	8.782168	2.648574
H	3.312247	17.078945	0.888601
C	4.645139	15.606363	1.112844
H	5.597454	13.867157	1.321826
H	-2.366884	5.440487	0.000000
C	-0.364938	5.235392	0.231381
H	1.598776	5.558075	0.347071
H	-0.559572	13.071388	-3.704557
C	0.851522	12.577793	-5.050259
H	2.450298	11.872266	-6.040521
H	3.049839	8.432139	6.461437
C	1.239051	8.001440	5.679173
H	-0.391005	7.797712	4.514391
H	5.397607	16.149181	1.179059
H	-0.335396	4.306995	0.285534
H	0.465730	13.018063	-5.772217
H	0.886278	7.585780	6.431900

Molecule 2: Optimized Geometry

Co	1.5021361688913868E+00	1.0633669132868731E+01	4.3168597038266676E-01
S	9.3732690311502898E-01	1.2815991991512165E+01	7.9971141658651235E-01
S	-5.6683133161710919E-01	9.7016344505230734E+00	3.3733605039838505E-01
S	2.6966966848893335E+00	1.0253109249590404E+01	-1.4708666601582538E+00
S	3.0118181492340179E+00	9.8539392457051491E+00	1.9509968065769403E+00
C	2.3905547370552038E+00	1.3819153413304084E+01	8.8198215133803182E-01
C	-4.4571035022474148E-01	7.9449498640214307E+00	2.2182564429459031E-01
C	1.9502732869153809E+00	1.1113430345278843E+01	-2.8173922584621867E+00
C	2.3113181504380975E+00	9.1477378476893563E+00	3.4057476511042708E+00
C	2.2743502102756175E+00	1.5229910971372744E+01	8.1715839775221932E-01
C	3.6874694500333418E+00	1.3282239861970044E+01	1.0507148688139432E+00
C	-1.6245434493887014E+00	7.1803495918324387E+00	4.3183435769965008E-02
C	7.7012004499713471E-01	7.2388957182539704E+00	3.7405738812769046E-01
C	7.3478802207453819E-01	1.1823439668605745E+01	-2.6884862539867260E+00
C	2.5705380912525762E+00	1.1113808732302731E+01	-4.0915909630242915E+00
C	3.0933017309353046E+00	9.0384754927164064E+00	4.5807314948517730E+00
C	9.9977727645773518E-01	8.6274846455134320E+00	3.4410390682814578E+00
H	1.2888916741295124E+00	1.5674634529535989E+01	6.9100085214705509E-01
C	3.3966849500551652E+00	1.6053630675588426E+01	9.3123658286867927E-01
C	4.7979114686504820E+00	1.4117192051379421E+01	1.1859614912237724E+00
H	3.8032297495180196E+00	1.2202298117580044E+01	1.1052399060618796E+00
H	-2.5709634030795132E+00	7.7052784478344281E+00	-8.1865094600488908E-02
C	-1.5955272768848825E+00	5.7838451294734332E+00	5.0509244805754097E-02
C	7.9120726550120546E-01	5.8409020272028691E+00	3.8172324484884579E-01
H	1.6915854246007156E+00	7.8033658477017491E+00	5.1574669224819980E-01
H	2.4752205351717077E-01	1.1856625657295327E+01	-1.7171885675804046E+00
C	1.7861369615019587E-01	1.2498734331876367E+01	-3.7740355903623155E+00
C	1.9900141642481950E+00	1.1766382308505843E+01	-5.1827104812867759E+00
H	3.5277140940198990E+00	1.0605820068176616E+01	-4.2108248407618367E+00
H	4.1040121681588859E+00	9.4441847966779591E+00	4.5817639154974179E+00
C	2.5935355603723504E+00	8.4080456027331341E+00	5.7221681207065753E+00
C	5.1147953923929490E-01	7.9993855647064063E+00	4.5851006535608327E+00
H	3.6856541420718114E-01	8.7434323608407833E+00	2.5629311079583470E+00
H	3.2763532147739705E+00	1.7134902992454602E+01	8.8128795870475019E-01
C	4.6706403146119850E+00	1.5508515600575114E+01	1.1274560680392762E+00
H	5.7862301135334553E+00	1.3677826057401806E+01	1.3233131488274672E+00
H	-2.5244686243020942E+00	5.2272437870012700E+00	-6.9330319402651108E-02
C	-3.8536555377533921E-01	5.0999567299075261E+00	2.2487916919573628E-01
H	1.7413268386246548E+00	5.3262808181181702E+00	5.2409491276848252E-01
H	-7.4109770754144977E-01	1.3061054095162682E+01	-3.6292718166800353E+00
C	7.8597907644037723E-01	1.2466012076162718E+01	-5.0343618278295335E+00
H	2.4821568136176060E+00	1.1738722126667746E+01	-6.1539037605573261E+00
H	3.2244103005314324E+00	8.3276124693871072E+00	6.6062871275251602E+00
C	1.2990956072513553E+00	7.8713029746497165E+00	5.7347385116731244E+00
H	-5.0427278144514853E-01	7.6090401280204496E+00	4.5750336200224728E+00
H	5.5455074125984867E+00	1.6152042724439880E+01	1.2029824554520070E+00
H	-3.6509128720666001E-01	4.0130242870747486E+00	2.4085658991052109E-01
H	3.3490395973452119E-01	1.2979488607267934E+01	-5.8794766206711389E+00
H	9.1379022979071378E-01	7.3641554271740075E+00	6.6171471869229741E+00

Molecule 3: Geometry from the X-ray measurement

Co	5.813640	7.195335	24.923509
N	7.102973	7.893493	23.377232
N	7.812178	6.934663	25.688300
N	4.738277	6.469649	23.230776
N	5.017674	5.285139	25.553561
N	4.704983	8.916964	24.358484
N	5.099891	8.083614	26.769142
O	6.673800	8.432215	22.205587
C	8.411594	7.771347	23.523687
N	8.415174	6.472120	26.786716
C	8.774778	7.248909	24.791992
O	4.597393	7.068988	22.047415
C	4.227298	5.244389	23.298146
N	4.902836	4.523556	26.654906
C	4.365063	4.621406	24.575238
O	4.549894	9.329798	23.078462
C	4.149390	9.694594	25.266508
N	5.116575	7.840208	28.063809
C	4.346212	9.209569	26.578750
B	5.163516	8.517389	21.977117
C	9.413896	8.222885	22.489711
H	7.973536	6.174342	27.577576
C	9.812378	6.493369	26.625615
C	10.030142	6.988734	25.345594
C	3.495845	4.587847	22.173367
H	5.328296	4.685075	27.466270
C	4.219897	3.402748	26.385428
C	3.857488	3.434803	25.093690
C	3.496265	10.961935	24.947235
H	5.596444	7.051988	28.306925
C	4.508294	8.761979	28.854668
C	3.935316	9.661297	27.847054
C	4.812625	9.116471	20.629726
H	10.321658	8.035821	22.808984
H	9.315422	9.187759	22.337397
H	9.258534	7.743879	21.649056
H	10.468036	6.225818	27.258303
H	10.871935	7.124007	24.926731
H	3.211047	3.691193	22.445774
H	4.089968	4.518463	21.394224
H	2.710543	5.122851	21.936109
H	4.025215	2.706248	27.000541
H	3.355273	2.778690	24.627962
H	3.154604	11.370720	25.767386
H	2.752283	10.799824	24.329193
H	4.142665	11.565732	24.525443
H	4.472148	8.815681	29.803701
H	3.381747	10.413588	28.019872
H	3.841359	8.966105	20.486199
H	4.943163	10.093905	20.700025
C	5.541938	8.639881	19.349705
H	4.984602	8.858812	18.558845
H	5.642286	7.655423	19.381925

C	6.921563	9.272667	19.171029
H	7.516976	8.964660	19.900377
H	6.839221	10.256733	19.241327
C	7.548899	8.920098	17.832425
H	7.598852	7.934887	17.753339
H	6.964801	9.254032	17.106006
C	8.963292	9.510745	17.650820
H	9.575426	9.095432	18.306941
H	8.934208	10.483037	17.832425
C	9.503203	9.288909	16.271209
H	9.523850	8.317081	16.083746
H	8.896681	9.712328	15.615089
C	10.930397	9.870113	16.092534
H	11.522923	9.480751	16.783804
H	10.898646	10.849013	16.241918
C	11.529607	9.600788	14.721710
H	11.569906	8.622625	14.572325
H	10.939688	9.989276	14.027511
C	12.921689	10.183489	14.575254
H	12.889909	11.165257	14.707064
H	13.519315	9.802370	15.269454
C	13.503715	9.876832	13.178069
H	13.542199	8.896074	13.052117
H	12.897700	10.247034	12.486798
C	14.933762	10.476105	12.975960
H	15.552235	10.059182	13.629152
H	14.906510	11.447332	13.166352
C	15.462911	10.262375	11.596349
H	15.428039	9.297892	11.376666
H	14.891945	10.745153	10.949015
C	16.949494	10.774415	11.467468
H	17.514637	10.294994	12.123589
H	16.978580	11.738298	11.690080
C	17.528531	10.570185	10.070282
H	17.575486	9.599559	9.876961
H	16.921192	10.980532	9.402445
C	18.933691	11.182690	9.915039
H	19.555251	10.729500	10.533082
H	18.898323	12.140758	10.155226
C	19.431470	11.036266	8.509066
H	20.326507	11.436948	8.441697
H	18.823100	11.499172	7.893953
H	19.479932	10.089388	8.271808

Molecule 3: Optimized Geometry

Co	9.1494674933	1.4059974732	4.4026895178
N	7.8662570734	0.6747332092	5.7797359658
N	7.1861993116	1.7153856126	3.5504066823
N	10.2185852399	2.2015037834	6.0469471291
N	9.9755063377	3.2833432163	3.6863460036
N	10.2464552516	-0.3452064460	5.0442416341
N	9.8047896320	0.4131373285	2.6402157599
O	8.1992954283	0.3383739335	7.0550922852
C	6.4472569301	0.7977403920	5.6186501430
N	6.6040127407	2.2045964366	2.4247329965
C	6.1319263172	1.3680929651	4.3830672168
O	10.3366877610	1.5561900567	7.2449427199
C	10.8021525609	3.3705741182	5.9508495187
N	10.0378074273	4.0559080021	2.5747895929
C	10.6503781814	3.9743856414	4.6403766904
O	10.2905077821	-0.7682461348	6.3464195049
C	10.7621058411	-1.1619213776	4.1650971959
N	9.8427142427	0.6885724853	1.3176766693
C	10.5819083474	-0.6903496821	2.8074915392
B	9.6893074720	0.1983015199	7.3324723993
C	5.4844098612	0.3106920619	6.6340378077
H	7.1836725818	2.5459703501	1.6242309942
C	5.2388911890	2.1980966857	2.5094550438
C	4.8949623021	1.6666274121	3.7335916385
C	11.6100315075	4.0281273145	7.0134933578
H	9.5790349018	3.7503547931	1.6842697177
C	10.7262138153	5.2001574734	2.7980380674
C	11.1367838428	5.1893148430	4.1250508258
C	11.4298676570	-2.4440002944	4.5122582398
H	9.3341856407	1.5090735149	0.9160079105
C	10.6055716154	-0.2106171836	0.6464342870
C	11.0958847492	-1.1253211605	1.5692326010
C	9.9382547890	-0.3686742103	8.8041575476
H	4.4537181855	0.5210438898	6.3324926210
H	5.5767638675	-0.7766063348	6.7912242926
H	5.6622728259	0.7827589091	7.6129839317
H	4.6369894566	2.6034023073	1.7008483081
H	3.8976680856	1.5091777894	4.1189309345
H	11.3208010706	5.0791236024	7.1452146288
H	11.5077924305	3.4959735874	7.9625233578
H	12.6726476114	4.0414838465	6.7211481406
H	10.9097600193	5.9130375722	2.0053590300
H	11.7132114836	5.9493437148	4.6358394399
H	11.9840187504	-2.8442688617	3.6605574584
H	12.1148810903	-2.3049700058	5.3577310595
H	10.6869674598	-3.1971939245	4.8176349797
H	10.7538070953	-0.1366684396	-0.4226615799
H	11.7191213033	-1.9861755581	1.3597974243
H	10.9836633913	-0.1225647254	9.0517962512
H	9.9011725128	-1.4686507932	8.7832859137
C	9.0144556345	0.1486033663	9.9229169693
H	9.5432072930	0.0894662513	10.8860571994
H	8.8034546006	1.2201122912	9.7736833412
C	7.6892542919	-0.6129872312	10.0698371894

H	7.0713294639	-0.4753894456	9.1707692205
H	7.8969795723	-1.6949250801	10.1251545027
C	6.9072549767	-0.1924517450	11.3197851878
H	6.7307435013	0.8946796295	11.3002310908
H	7.5338290833	-0.3637266107	12.2089348385
C	5.5716404231	-0.9212987833	11.4923886302
H	4.9182593964	-0.7007193753	10.6328977740
H	5.7382493058	-2.0101713063	11.4593428820
C	4.8488271330	-0.5601148520	12.7947712031
H	4.6849373262	0.5284498591	12.8372483825
H	5.5062236933	-0.7835735595	13.6495542473
C	3.5100318364	-1.2828388391	12.9804532562
H	2.8507722953	-1.0604355126	12.1267187831
H	3.6694325490	-2.3725588773	12.9458673922
C	2.8012274201	-0.9112987326	14.2861772763
H	2.6460438950	0.1787307252	14.3227056046
H	3.4617472102	-1.1337902985	15.1384870542
C	1.4575468844	-1.6198122724	14.4856122296
H	1.6086383939	-2.7114618942	14.4743756138
H	0.7996711586	-1.4108988593	13.6271958261
C	0.7547560422	-1.2139747963	15.7848021781
H	0.6078488717	-0.1225791679	15.7964762699
H	1.4149937803	-1.4236954623	16.6399106966
C	-0.5922506421	-1.9090100239	15.9931711118
H	-1.2523198438	-1.6880748581	15.1394059575
H	-0.4499425117	-3.0019559339	15.9685547124
C	-1.3023961227	-1.5283308063	17.2984274725
H	-1.4228906569	-0.4345732463	17.3589888783
H	-0.6669512134	-1.7994625935	18.1564064474
C	-2.6681541242	-2.2133585235	17.4188990553
H	-3.3087568801	-1.8921763854	16.5826106114
H	-2.5380125539	-3.2981008233	17.2748680650
C	-3.3982099302	-1.9852471343	18.7451396996
H	-3.5617885138	-0.9089666644	18.9192234544
H	-2.7579281832	-2.3260620987	19.5715071978
C	-4.7394934646	-2.7278566828	18.8065273712
H	-5.4551037206	-2.2431396320	18.1262229549
H	-4.6020029106	-3.7423169026	18.4081249708
C	-5.3255382723	-2.8111615320	20.2180081346
H	-6.3234260993	-3.2720935420	20.2128975966
H	-4.6824658480	-3.4176207102	20.8717025960
H	-5.4259637320	-1.8179107772	20.6709535180

Molecule 4: Geometry from the X-ray measurement

Co	11.239827	6.027416	1.416757
N	9.810385	7.571012	1.528654
N	12.353293	7.841664	1.305696
N	10.022796	5.176912	2.876154
N	12.555155	5.170487	2.894270
N	9.741640	5.213052	0.133775
N	12.280944	5.118284	-0.238286
O	8.428801	7.389507	1.677757
C	10.132746	8.812636	1.510538
N	13.654720	8.284183	1.209546
C	11.593072	8.982898	1.393486
O	8.626105	5.223492	2.837137
C	10.496061	4.533612	3.917088
N	13.950724	4.936779	3.046160
C	11.984170	4.532809	3.885038
O	8.380232	5.276498	0.353945
C	10.031490	4.598665	-0.916913
N	13.588341	4.851648	-0.597805
C	11.512423	4.489441	-1.123149
B	8.036438	5.928632	1.665215
C	9.177950	9.987601	1.542589
H	14.349507	7.745289	1.164954
C	13.753227	9.623787	1.192824
C	12.427152	10.120919	1.339140
C	9.722659	3.930469	4.983105
H	14.588484	5.225902	2.515242
C	14.106008	4.217986	4.117750
C	12.925685	3.891920	4.762934
C	9.051537	4.009978	-1.909075
H	14.301623	5.123906	-0.161644
C	13.593138	4.086275	-1.757185
C	12.269058	3.803576	-2.095802
C	6.391618	5.856351	1.927191
H	8.257872	9.659125	1.624804
H	9.268682	10.504007	0.714858
H	9.390994	10.560225	2.309006
H	14.548212	10.136178	1.100854
H	12.160177	11.030854	1.389305
H	8.768648	4.052544	4.801952
H	9.952455	4.358532	5.834524
H	9.927037	2.973150	5.033270
H	14.959723	3.939304	4.429891
H	12.781532	3.390773	5.557221
H	8.137054	4.183452	-1.605295
H	9.196103	3.043022	-1.975963
H	9.187029	4.421979	-2.786971
H	14.363890	3.804379	-2.236544
H	11.951292	3.272714	-2.816234
H	5.933768	6.384804	1.227661
H	6.094654	4.915898	1.836614
C	5.962676	6.378379	3.301168
H	6.175634	7.342926	3.365268
H	6.472376	5.904538	4.004878

C	4.443360	6.172780	3.540847
H	3.923005	6.783152	2.959764
H	4.190877	5.241964	3.320676
C	4.137360	6.466722	5.016548
H	4.354732	7.411191	5.217210
H	4.703544	5.892491	5.590664
C	2.630957	6.200086	5.337050
H	2.060020	6.808851	4.804739
H	2.396838	5.267664	5.101551
C	2.402182	6.434597	6.811358
H	2.654179	7.363004	7.044070
H	2.971569	5.819408	7.338095
C	0.928530	6.197677	7.158336
H	0.680047	5.267664	6.929804
H	0.359557	6.808048	6.624631
C	0.673413	6.447447	8.688383
H	1.263939	5.850729	9.210940
H	0.924002	7.381476	8.904373
C	-0.809021	6.207314	9.138479
H	-1.052021	5.269270	8.937817
H	-1.401332	6.791183	8.603380
C	-1.079458	6.476360	10.649017
H	-0.532795	5.863579	11.202231
H	-0.823804	7.406373	10.874762
C	-2.568111	6.260320	10.951404
H	-2.826561	5.334323	10.715905
H	-3.114593	6.882738	10.409338
C	-2.837227	6.518925	12.510714
H	-2.295216	5.886870	13.047206
H	-2.554124	7.438497	12.746213
C	-4.329318	6.337420	12.853512
H	-4.631278	5.437926	12.570634
H	-4.868781	7.012041	12.371366
C	-4.524599	6.503666	14.397494
H	-4.005554	5.812179	14.878246
H	-4.197695	7.393523	14.683158
C	-6.028367	6.363120	14.740291
H	-6.547608	7.066653	14.274867
H	-6.361545	5.481294	14.437905
C	-6.216839	6.496438	16.259190
H	-7.166259	6.404882	16.479361
H	-5.895539	7.375051	16.551822
H	-5.706232	5.794511	16.714860

Molecule 4: Optimized Geometry

Co	11.0279949431	5.9259884586	1.2717426697
N	9.6086589923	7.4879629924	1.4255215889
N	12.1584289469	7.7097261706	1.2580823498
N	9.7639529916	5.1291064778	2.7588622666
N	12.3398621970	5.1358111792	2.8123877161
N	9.5471477031	5.1001136811	-0.0131225006
N	12.1375159459	5.0355237579	-0.3561912034
O	8.2573153755	7.3025002122	1.4601122390
C	9.9971472026	8.7363105234	1.3821596716
N	13.4549660952	8.0911096543	1.2086449101
C	11.4397828686	8.8622959365	1.3056411363
O	8.4015925809	5.1420229889	2.6744026405
C	10.2513117999	4.4810839677	3.7856371942
N	13.6586265234	4.9227779440	3.0335333399
C	11.6984492677	4.4639132815	3.8033659183
O	8.1966582929	5.1833472506	0.1911163472
C	9.9153009990	4.4476293332	-1.0841282112
N	13.4117580271	4.7268691824	-0.6937024720
C	11.3580143955	4.3568328170	-1.2385766060
B	7.8073777232	5.8609037745	1.4825373449
C	9.0561072540	9.8894408628	1.3718037710
H	14.2172312117	7.3868587935	1.1034411990
C	13.5833633193	9.4426522487	1.2101010894
C	12.3030524187	9.9777741538	1.2798314157
C	9.4091011126	3.7954378856	4.8035247960
H	14.3751059952	5.2990300242	2.3743361202
C	13.8693699406	4.1379334226	4.1206507312
C	12.6255030074	3.8209434961	4.6488585208
C	8.9578490749	3.8393891022	-2.0419388866
H	14.2141392541	5.1351423571	-0.1635725163
C	13.4654973000	3.8822057252	-1.7482062213
C	12.1545032663	3.6187126873	-2.1332629805
C	6.2204638459	5.8154071461	1.6478626162
H	8.3125645361	9.7991045826	2.1761591744
H	8.4744248053	9.9044471754	0.4377559728
H	9.5840823656	10.8393842404	1.4693288174
H	14.5576314509	9.9076774334	1.1142190115
H	12.0382244225	11.0249349100	1.3065047311
H	8.6068042671	3.2357387546	4.3073385521
H	8.9120266918	4.5238864013	5.4602501373
H	10.0094906070	3.1224314531	5.4229612426
H	14.8631233286	3.8477247404	4.4431183301
H	12.4253310249	3.2008747684	5.5109788600
H	8.1227374286	3.3749385631	-1.5028366106
H	9.4529734012	3.1069259563	-2.6844266479
H	8.5198674149	4.6158333609	-2.6869715765
H	14.4144458545	3.5083518697	-2.1143232076
H	11.8378828653	2.9785155133	-2.9442878167
H	5.7435423927	6.3785300475	0.8302079151
H	5.8861554649	4.7718673512	1.5282017034
C	5.7539262501	6.3508528376	3.0113976406
H	5.9862721015	7.4247817737	3.0837975653
H	6.3436624416	5.8685996692	3.8057609482

C	4.2708699411	6.1311916907	3.3242596235
H	3.6460596919	6.7186826423	2.6335525025
H	4.0087450432	5.0777392157	3.1332697110
C	3.9270635148	6.4848724135	4.7754890900
H	4.1522713861	7.5469205061	4.9623692797
H	4.5979994456	5.9261173391	5.4478009631
C	2.4724526615	6.1939913457	5.1550335469
H	1.7997981646	6.7883410282	4.5170380901
H	2.2376166970	5.1415884467	4.9277481929
C	2.1636740516	6.4787135390	6.6283762175
H	2.3862846853	7.5335707236	6.8548618607
H	2.8481651955	5.8955161077	7.2635737923
C	0.7133399678	6.1676230959	7.0128402270
H	0.4878311811	5.1148750673	6.7779013417
H	0.0342806517	6.7604549305	6.3796652757
C	0.3998284962	6.4425730744	8.4870195453
H	1.0724367783	5.8472433532	9.1225016489
H	0.6328674470	7.4933332971	8.7217204542
C	-1.0566389397	6.1478007368	8.8621140655
H	-1.2926567591	5.0965013182	8.6313191129
H	-1.7237044406	6.7453007758	8.2206279750
C	-1.3769776237	6.4363985983	10.3326352107
H	-0.7255043469	5.8272361594	10.9768422495
H	-1.1187609991	7.4818613919	10.5645284947
C	-2.8439913721	6.1822475148	10.6975121757
H	-3.1059595617	5.1362489474	10.4711991760
H	-3.4875989775	6.7947319170	10.0469449949
C	-3.1656504501	6.4882799506	12.1641597003
H	-2.5242170446	5.8729784870	12.8131706740
H	-2.8891141352	7.5306295712	12.3885232437
C	-4.6356052070	6.2594563182	12.5402305729
H	-4.9195079174	5.2216188337	12.3019696111
H	-5.2824183527	6.8954147893	11.9150489702
C	-4.9168231894	6.5400423015	14.0207139782
H	-4.2616303459	5.9083986893	14.6390754177
H	-4.6317406088	7.5762206257	14.2604720907
C	-6.3766647129	6.3057976809	14.4343330766
H	-7.0308303055	6.9838038570	13.8641332720
H	-6.6695373665	5.2834262968	14.1453937462
C	-6.5874877602	6.5055314113	15.9399894858
H	-7.6206690781	6.3005489946	16.2512928908
H	-6.3560143952	7.5342382503	16.2463761714
H	-5.9407689343	5.8376613982	16.5227295574

Molecule 5: Geometry from the X-ray measurement

Br	-0.634685	2.868628	2.270771
Co	-1.238792	1.610055	4.160751
P	-3.163408	0.372127	3.490038
Br	-1.842899	2.868628	6.050730
P	0.685823	0.372127	4.831464
C	-2.873031	-0.820012	2.145283
C	-4.023111	-0.581085	4.776542
C	-4.431106	1.510155	2.837632
C	0.395446	-0.820012	6.176218
C	1.545526	-0.581085	3.544959
C	1.953521	1.510155	5.483869
C	-2.142958	-0.405427	1.051838
C	-3.312138	-2.138692	2.201869
C	-3.280530	-1.418580	5.583727
C	-5.385779	-0.484515	5.011208
C	-4.706451	2.665665	3.539967
C	-5.100621	1.239593	1.645993
C	-0.334626	-0.405427	7.269663
C	0.834553	-2.138692	6.119632
C	0.802945	-1.418580	2.737774
C	2.908194	-0.484515	3.310293
C	2.228866	2.665665	4.781535
C	2.623036	1.239593	6.675508
C	-1.879469	-1.262902	0.000000
H	-1.906716	0.333000	0.882079
C	-3.003431	-2.993670	1.165010
H	-3.570014	-2.580750	3.012383
C	-3.895438	-2.175322	6.592293
H	-2.386123	-1.581750	5.392333
C	-5.990505	-1.240425	6.001467
H	-5.958011	0.249750	4.676684
C	-5.714479	3.516480	3.103920
H	-4.161428	2.913750	4.243966
C	-6.115091	2.086245	1.231582
H	-5.203859	0.249750	1.547799
C	-0.598115	-1.262902	8.321501
H	-0.570869	0.333000	7.439422
C	0.525846	-2.993670	7.156491
H	1.092429	-2.580750	5.309118
C	1.417853	-2.175322	1.729208
H	-0.091462	-1.581750	2.929168
C	3.512921	-1.240425	2.320035
H	3.480426	0.249750	3.644817
C	3.236894	3.516480	5.217581
H	1.683844	2.913750	4.077536
C	3.637506	2.086245	7.089919
H	2.726274	0.249750	6.773702
C	-2.294345	-2.562435	0.066572
H	-1.259674	-0.999000	-0.615791
H	-3.495028	-3.912750	1.131724
C	-5.224540	-2.072925	6.793673
H	-3.450504	-2.830500	7.139848
H	-6.856799	-0.999000	6.424199

C	-6.355910	3.230933	1.917274
H	-5.985487	4.329000	3.378529
H	-6.032602	2.247750	0.199716
C	-0.183240	-2.562435	8.254929
H	-1.217910	-0.999000	8.937292
H	1.017443	-3.912750	7.189777
C	2.746955	-2.072925	1.527828
H	0.972919	-2.830500	1.181653
H	4.379215	-0.999000	1.897302
C	3.878326	3.230933	6.404227
H	3.507903	4.329000	4.942972
H	3.555017	2.247750	8.121785
H	-2.224615	-3.163500	-0.632434
H	-5.634373	-2.580750	7.522637
H	-7.148606	3.496500	1.564442
H	-0.252969	-3.163500	8.953935
H	3.156788	-2.580750	0.798864
H	4.671021	3.496500	6.757059

Molecule 5: Optimized Geometry

Br	-0.4832042485	2.9589736745	2.3726412975
Co	-1.0314086262	1.5948741783	4.2150195383
P	-2.8968601705	0.4154598331	3.5814510876
Br	-1.5796127505	2.9589750764	6.0573972721
P	0.8340453530	0.4154624676	4.8485897399
C	-2.6272465909	-0.7817418021	2.2340011708
C	-3.7745577129	-0.5264962269	4.8805388090
C	-4.1724920502	1.5517346655	2.9219950396
C	0.5644321275	-0.7817374069	6.1960414481
C	1.7117442078	-0.5264952881	3.5495038173
C	2.1096766030	1.5517387486	5.5080443034
C	-1.8878233237	-0.3605653529	1.1126559851
C	-3.1304406492	-2.0920729880	2.2795174823
C	-3.0335925452	-1.4191289302	5.6754840687
C	-5.1514457200	-0.3718560057	5.1180510365
C	-4.4716982531	2.7217740232	3.6431105694
C	-4.8714706754	1.2724460748	1.7354603112
C	-0.1749898663	-0.3605593472	7.3173868806
C	1.0676247548	-2.0920691525	6.1505258601
C	0.9707805721	-1.4191297528	2.7545590537
C	3.0886324142	-0.3718546320	3.3119928029
C	2.4088794495	2.7217791206	4.7869290653
C	2.8086585839	1.2724499569	6.6945770259
C	-1.6753421845	-1.2344223329	0.0475180873
H	-1.4772678135	0.6474883548	1.0795168596
C	-2.9002616936	-2.9664671494	1.2142373920
H	-3.6868464462	-2.4337576308	3.1470047832
C	-3.6565198048	-2.1238961952	6.7072876054
H	-1.9682985071	-1.5621347156	5.5022665206
C	-5.7742607263	-1.0936248373	6.1403207048
H	-5.7300778052	0.3259778814	4.5177870632
C	-5.4742971375	3.5825577267	3.1913839703
H	-3.9138451820	2.9592111339	4.5486553475
C	-5.8666841063	2.1438210734	1.2841362101
H	-4.6313954478	0.3775224386	1.1647121577
C	-0.3874713279	-1.2344153794	8.3825255102
H	-0.5855442665	0.6474948099	7.3505254644
C	0.8374456893	-2.9664622875	7.2158067945
H	1.6240295608	-2.4337551058	5.2830384640
C	1.5937094975	-2.1238983176	1.7227573469
H	-0.0945136246	-1.5621358171	2.9277753372
C	3.7114490767	-1.0936246874	2.2897249703
H	3.6672634292	0.3259806351	3.9122562185
C	3.4114780413	3.5825639810	5.2386541156
H	1.8510241270	2.9592159125	3.8813855999
C	3.8038717321	2.1438260729	7.1458996143
H	2.5685862499	0.3775252560	7.2653247556
C	-2.1777525830	-2.5390057905	0.0988955116

H	-1.1054350167	-0.9074845308	-0.8188722110
H	-3.2807766303	-3.9839238679	1.2769480733
C	-5.0241986319	-1.9568323649	6.9442950781
H	-3.0638397247	-2.7835880712	7.3378285175
H	-6.8401629364	-0.9604426765	6.3188603842
C	-6.1728137469	3.2955416488	2.0147279995
H	-5.7031267401	4.4845356043	3.7571411833
H	-6.4146168307	1.9241420706	0.3695773801
C	0.1149377084	-2.5389994117	8.3311488143
H	-0.9573777058	-0.9074764340	9.2489158918
H	1.2179596103	-3.9839194245	7.1530964888
C	2.9613884888	-1.9568340127	1.4857511100
H	1.0010305517	-2.7835916903	1.0922169103
H	4.7773514196	-0.9604420750	2.1111863177
C	4.1099977395	3.2955479316	6.4153082675
H	3.6403051510	4.4845426084	4.6728970130
H	4.3518071409	1.9241469504	8.0604568115
H	-1.9846082439	-3.2153829359	-0.7307956055
H	-5.4981202740	-2.4732992272	7.7777573069
H	-6.9539383831	3.9678227814	1.6673182253
H	-0.0782069165	-3.2153757166	9.1608405383
H	3.4353113487	-2.4733017530	0.6522900939
H	4.8911222026	3.9678299940	6.7627167132

Molecule 6: Geometry from the X-ray measurement

Co	11.044075	1.049033	3.343833
N	10.129890	-0.709778	2.995719
N	12.763688	1.147475	4.380638
N	9.203588	1.512595	3.968930
N	12.132218	2.149158	2.069879
S	10.911085	-2.028783	2.572360
C	8.768876	-0.757896	3.381089
S	13.086350	0.195189	5.607623
C	13.810062	1.853097	3.735184
S	8.778233	3.009365	4.338260
C	8.260273	0.468710	3.888088
S	11.436216	3.021514	0.927285
C	13.461878	2.406053	2.476832
O	10.274268	-2.765098	1.496671
O	12.279513	-1.599559	2.301248
C	11.003812	-3.088815	3.983562
C	7.934815	-1.870202	3.306649
O	11.831675	-0.457679	5.965832
O	13.745286	0.856690	6.719380
C	14.178662	-1.054596	5.000852
C	15.091474	2.062514	4.237610
O	7.932628	3.087376	5.514618
O	10.002041	3.777012	4.382101
C	7.812799	3.620669	2.987123
C	6.915665	0.527698	4.273824
O	12.190188	3.072829	-0.307089
O	10.078604	2.538577	0.821770
C	11.350659	4.677504	1.548066
C	14.427920	3.121746	1.755657
H	11.425035	-2.620105	4.707846
H	11.518613	-3.868612	3.764082
H	10.118588	-3.352264	4.243280
H	8.267686	-2.672859	2.973954
C	6.604548	-1.797786	3.725490
H	13.766918	-1.507480	4.261570
H	14.363631	-1.684924	5.700993
H	14.999233	-0.649032	4.709675
H	15.323718	1.705706	5.064501
C	16.033210	2.800587	3.517167
H	6.989190	3.130059	2.933716
H	8.302930	3.513723	2.169194
H	7.622362	4.551214	3.125761
C	6.102750	-0.600274	4.185849
H	6.561405	1.326838	4.590790
H	10.855060	4.685497	2.370384
H	10.910963	5.238612	0.903526
H	12.238071	5.006815	1.706457
H	14.215364	3.465765	0.918158
C	15.701433	3.322690	2.280214
H	6.061916	-2.552964	3.694580
H	16.882948	2.941424	3.868335
H	5.209991	-0.543524	4.440812
H	16.329986	3.809464	1.797907

Molecule 6: Optimized Geometry

Co	10.9501	1.0203	3.3002
S	8.7166	3.0222	4.3474
S	11.3265	3.0638	0.9002
S	10.8323	-2.0890	2.5588
S	12.9626	0.1574	5.6065
O	11.6883	-0.5245	5.9414
O	12.0945	3.1528	-0.3528
O	7.8497	3.0917	5.5377
O	9.9388	2.5933	0.7909
N	10.0375	-0.7327	2.9487
O	13.6068	0.8270	6.7515
O	10.2027	-2.8516	1.4654
O	9.9752	3.7768	4.4102
O	12.2335	-1.6577	2.3170
N	12.6478	1.1293	4.3630
N	9.1246	1.5011	3.9405
N	12.0340	2.1303	2.0306
C	6.0024	-0.6008	4.1845
H	4.9648	-0.5162	4.4965
C	13.3603	2.3837	2.4504
C	8.6850	-0.7737	3.3494
C	7.7581	3.6633	2.9819
H	6.8740	3.0300	2.8458
H	8.3785	3.6406	2.0790
H	7.4420	4.6809	3.2396
C	14.3412	3.1078	1.7442
H	14.1064	3.5066	0.7606
C	13.6977	1.8295	3.7238
C	10.8964	-3.1119	4.0103
H	11.3274	-2.5040	4.8124
H	11.5330	-3.9755	3.7836
H	9.8868	-3.4383	4.2804
C	11.2730	4.6970	1.6038
H	10.7958	4.6034	2.5848
H	10.6907	5.3245	0.9202
H	12.2909	5.0861	1.7083
C	14.9855	2.0466	4.2446
H	15.2256	1.6806	5.2380
C	15.6128	3.3222	2.2885
H	16.3450	3.9065	1.7353
C	6.8268	0.5263	4.2512
H	6.4305	1.4546	4.6482
C	14.0839	-1.0722	4.9725
H	13.5406	-1.7003	4.2605
H	14.4771	-1.6627	5.8025
H	14.8960	-0.5445	4.4598
C	8.1766	0.4633	3.8639
C	7.8374	-1.8930	3.2985
H	8.2188	-2.8326	2.9099
C	6.5086	-1.8181	3.7294
H	5.8793	-2.7066	3.6944
C	15.9324	2.7950	3.5395
H	16.9053	2.9840	3.9877

Molecule 7: Geometry from the X-ray measurement

C	2.109529	15.345519	10.019756
C	2.376109	16.336937	8.896010
C	1.542646	14.034660	9.452130
C	1.080249	15.949781	11.005432
P	3.625710	15.009409	10.966120
H	2.972965	15.930126	8.234418
H	1.528564	16.579197	8.464536
H	2.796034	17.143232	9.262281
H	0.674995	14.209373	9.032163
H	2.161412	13.673327	8.782868
H	1.429474	13.386649	10.177004
H	1.428007	16.792629	11.362116
H	0.237425	16.116411	10.533688
H	0.927712	15.321108	11.739894
N	4.698834	14.420140	9.904889
N	4.269702	16.367449	11.607192
O	3.279558	14.111540	12.131671
C	5.997231	13.840071	10.309322
H	4.468228	14.172881	9.204752
C	5.221322	17.302151	11.003514
H	4.084593	16.537218	12.426415
Dy	3.203958	13.758814	14.310513
H	6.189490	14.136456	11.245139
C	5.937864	12.317056	10.299734
C	7.104224	14.357065	9.417612
H	5.370798	17.023138	10.054274
C	4.663212	18.720346	10.992008
C	6.560479	17.265158	11.720718
O	3.308033	13.455605	16.489929
O	2.672348	16.049658	14.566903
O	0.850737	13.977880	14.342346
O	2.305139	11.587802	14.153073
O	5.000804	12.229517	14.034562
O	5.187356	15.006660	14.557124
H	5.223462	12.013254	10.896125
H	5.758572	12.002285	9.388847
H	6.796373	11.955145	10.606559
H	6.938508	14.075635	8.493301
H	7.129395	15.335841	9.459801
H	7.963298	13.996326	9.718684
H	3.791283	18.725267	10.543276
H	4.555989	19.036764	11.914401
H	5.279592	19.311308	10.514511
H	6.438717	17.545025	12.652699
H	6.918289	16.353001	11.697706
H	7.186285	17.873430	11.275821
P	3.406815	12.640182	17.756350
H	3.079710	16.397619	15.293312
H	1.782777	16.167412	14.666238
H	0.301200	13.325492	14.344072
H	0.403646	14.697687	14.401602
H	2.703741	11.134793	13.509891
H	1.443155	11.633737	13.973964

H	4.965851	11.421804	13.864657
H	5.769809	12.483131	14.037247
H	5.933216	14.739194	14.459131
H	5.216944	15.748318	14.861839
C	1.887567	12.926379	18.719973
N	3.588372	11.085978	17.289783
N	4.647084	12.994524	18.738190
C	0.715848	12.492991	17.836124
C	1.782052	14.418319	19.051920
C	1.888666	12.102653	20.013432
C	4.170782	9.987000	18.066242
H	3.387213	10.907953	16.478424
C	6.044611	13.118889	18.271433
H	4.481968	13.283180	19.515991
H	0.824326	11.550694	17.588747
H	-0.124749	12.608551	18.327045
H	0.697710	13.042813	17.024956
H	1.868251	14.942932	18.227327
H	0.912617	14.600463	19.464214
H	2.497529	14.666997	19.673241
H	2.007697	11.154262	19.795971
H	1.036953	12.225718	20.480574
H	2.624585	12.399061	20.589880
H	4.454553	10.340275	18.957954
C	5.398404	9.439822	17.349039
C	3.151688	8.876583	18.284857
H	6.106105	12.719735	17.356710
C	6.426829	14.583158	18.177467
C	6.966601	12.346805	19.193825
H	5.138447	9.103223	16.466919
H	6.060668	10.154206	17.245485
H	5.786260	8.708686	17.874477
H	2.371917	9.238235	18.756599
H	2.870390	8.515567	17.418076
H	3.555831	8.161732	18.821800
H	5.765877	15.059107	17.630936
H	6.446317	14.973460	19.076847
H	7.311734	14.664490	17.765171
H	7.884918	12.400534	18.858236
H	6.684282	11.408291	19.228342
H	6.926004	12.731394	20.095123
C	-1.392119	17.473125	16.767990
C	-2.416922	18.552286	17.120838
C	-1.968549	16.066599	17.009615
C	-0.135176	17.657707	17.638605
P	-0.882141	17.615459	15.026182
H	-2.597079	18.529160	18.083504
H	-3.247549	18.386551	16.628000
H	-2.062819	19.432640	16.875380
H	-2.196306	15.965426	17.956938
H	-1.300818	15.392161	16.760319
H	-2.773910	15.944679	16.465000
H	0.223502	18.560648	17.504370

H	0.541162	16.995478	17.381639
H	-0.370172	17.537355	18.582092
N	-0.206231	19.066221	14.728754
N	-2.272918	17.598228	14.174743
O	0.120437	16.534903	14.712837
C	1.230395	19.386015	14.831157
H	-0.685573	19.635344	14.462966
C	-2.288699	17.472874	12.706393
H	-2.989428	17.413143	14.593367
H	1.671634	18.653782	15.348924
C	1.440747	20.686144	15.588631
C	1.883750	19.449150	13.469621
H	-1.415471	17.803692	12.349709
C	-3.398166	18.352411	12.157943
C	-2.461162	16.023367	12.290262
H	1.042287	21.426289	15.084287
H	2.402282	20.847509	15.697937
H	1.017337	20.623926	16.468836
H	1.738676	18.603127	12.997877
H	2.844946	19.605974	13.575091
H	1.488681	20.182714	12.951853
H	-4.254536	18.078827	12.547227
H	-3.437362	18.258121	11.183773
H	-3.217822	19.287580	12.388063
H	-1.722814	15.489503	12.650781
H	-2.461740	15.961793	11.312257
H	-3.311673	15.683466	12.639275
C	-2.164009	11.089213	12.179038
C	-2.308547	12.590451	11.929742
C	-3.495853	10.359827	11.931660
C	-1.079541	10.526690	11.243221
P	-1.572263	10.813633	13.887095
H	-1.441190	13.027745	12.056308
H	-2.958889	12.965310	12.558734
H	-2.618417	12.740322	11.011184
H	-4.194087	10.756735	12.491616
H	-3.396266	9.411913	12.156025
H	-3.744849	10.446912	10.988173
H	-1.333317	10.692603	10.311240
H	-0.988648	9.562467	11.390880
H	-0.224481	10.968784	11.431151
N	-1.242425	9.237701	14.141184
N	-2.838138	11.127503	14.858579
O	-0.319199	11.605924	14.117021
C	0.071166	8.600075	13.952869
H	-1.873488	8.722899	14.401601

C	-2.757750	11.073922	16.336517
H	-3.409429	11.678246	14.526249
H	0.629295	9.201256	13.379491
C	0.774311	8.427886	15.268382
C	-0.108303	7.283284	13.218407
H	-1.874617	10.676217	16.583895
C	-2.826465	12.461657	16.925238
C	-3.847961	10.173484	16.883049
H	0.925117	9.305430	15.676843
H	1.635259	7.982520	15.122641
H	0.220878	7.880567	15.864774
H	-0.664702	6.682091	13.759186
H	0.767763	6.869697	13.066912
H	-0.545151	7.445387	12.357380
H	-2.113938	13.016177	16.541706
H	-2.712487	12.409962	17.897490
H	-3.696521	12.861803	16.718130
H	-4.722468	10.499626	16.581978
H	-3.816298	10.180499	17.861053
H	-3.710315	9.259181	16.557047
I	8.183882	13.541865	14.099763
I	5.063372	18.196983	15.869376
I	4.293308	8.964184	13.090500

Molecule 7: Optimized Geometry

C	1.561796	14.172413	9.520993
C	1.172912	16.105663	11.086142
P	3.723267	15.253415	10.893113
H	2.906048	16.084452	8.050001
H	1.325207	16.773994	8.460219
H	2.783580	17.436020	9.208571
H	0.551342	14.337206	9.117392
H	2.170252	13.740875	8.714690
H	1.485598	13.440447	10.335789
H	1.538860	17.057718	11.490953
H	0.195630	16.292924	10.620419
H	1.020390	15.415959	11.924718
N	4.787946	14.634680	9.809087
N	4.359909	16.684422	11.427644
O	3.441681	14.388041	12.128564
C	6.091847	14.071283	10.245193
H	4.441242	14.286595	8.907067
C	5.382108	17.519315	10.773667
H	4.140936	16.914959	12.399806
Dy	3.650507	14.288321	14.336456
H	6.256235	14.433341	11.270556
C	6.050849	12.541710	10.276678
C	7.216680	14.586473	9.344138
H	5.537366	17.067825	9.786649
C	4.890336	18.959959	10.583308
C	6.704442	17.488291	11.550283
O	4.024065	14.302721	16.524197
O	3.115665	16.621417	14.316952
O	1.305628	14.460665	14.599038
O	2.827404	12.012409	14.506393
O	5.460725	12.815783	13.957528
O	5.629756	15.642755	14.251188
H	5.256314	12.178961	10.940366
H	5.874657	12.136366	9.270353
H	7.006461	12.141663	10.643816
H	7.061893	14.245280	8.311669
H	7.262006	15.681913	9.334699
H	8.186440	14.205474	9.691881
H	3.956810	18.991378	10.007635
H	4.705638	19.440619	11.552155
H	5.644368	19.556917	10.050751
H	6.572834	17.899058	12.560319
H	7.089310	16.467436	11.658319
H	7.464792	18.093842	11.037876
P	4.209076	13.437713	17.778213
H	3.658247	17.235618	14.876030
H	2.143762	16.911774	14.422541
H	0.708485	13.702407	14.334378
H	0.816122	15.330771	14.610195
H	3.287992	11.272012	14.028968
H	1.836610	11.894971	14.327023
H	5.417410	11.873845	13.635369
H	6.415681	13.012315	14.152072

H	6.538410	15.241215	14.306791
H	5.672678	16.457460	14.817975
C	2.738496	13.703181	18.839765
N	4.392338	11.894883	17.226882
N	5.500710	13.721354	18.746285
C	1.518939	13.295310	17.992307
C	2.647835	15.200407	19.190950
C	2.814562	12.860677	20.121209
C	4.941276	10.740038	17.950938
H	3.929903	11.689475	16.337675
C	6.900070	13.813111	18.263939
H	5.328396	14.239078	19.614657
H	1.544801	12.234570	17.708822
H	0.610671	13.463453	18.583466
H	1.440209	13.892663	17.075945
H	2.664358	15.820971	18.286936
H	1.711321	15.398521	19.731737
H	3.467401	15.519684	19.847193
H	2.887724	11.786669	19.910866
H	1.901561	13.021769	20.709912
H	3.665470	13.144672	20.751965
H	5.266744	11.119932	18.928006
C	3.871645	9.668306	18.153679
C	6.147812	10.171342	17.199285
H	6.922173	13.380221	17.253978
C	7.814605	13.001409	19.187202
C	7.327195	15.278032	18.183900
H	3.002579	10.061004	18.694688
H	3.522494	9.284634	17.184877
H	4.271306	8.819786	18.722722
H	6.919326	10.936233	17.045570
H	5.845267	9.797514	16.210212
H	6.593987	9.334769	17.755469
H	7.549471	11.937289	19.186818
H	8.861082	13.095617	18.867575
H	7.732667	13.371217	20.218785
H	6.658389	15.847672	17.524849
H	7.293421	15.732987	19.183032
H	8.350327	15.368984	17.795210
C	-0.660016	18.009191	16.831940
C	-1.642888	19.099317	17.292521
C	-1.217049	16.600674	17.115391
C	0.688286	18.192303	17.557084
P	-0.318872	18.160041	15.026396
H	-1.731925	19.062040	18.388925
H	-2.647272	18.957515	16.873681
H	-1.286275	20.099880	17.015890
H	-1.398354	16.487076	18.194202
H	-0.499300	15.827230	16.816145
H	-2.168820	16.415337	16.601313
H	1.085955	19.204921	17.418409
H	1.442919	17.473156	17.212379
H	0.550359	18.036719	18.637076
N	0.394018	19.608749	14.660123
N	-1.786880	18.211140	14.294357

O	0.612184	17.019450	14.600941
C	1.842217	19.895893	14.680603
H	-0.215711	20.357532	14.317148
C	-1.938807	17.955293	12.843912
H	-2.630224	17.995058	14.843079
H	2.314737	19.131357	15.310561
C	2.114888	21.259235	15.316113
C	2.471035	19.826427	13.286859
H	-1.010565	18.297151	12.365137
C	-3.112648	18.783765	12.326589
C	-2.130522	16.462801	12.562412
H	1.616372	22.061589	14.753965
H	3.194419	21.459224	15.312180
H	1.765161	21.298512	16.353751
H	2.260804	18.864417	12.804354
H	3.561571	19.936330	13.370966
H	2.084370	20.632053	12.648626
H	-4.017134	18.527588	12.894160
H	-3.306900	18.562068	11.269708
H	-2.917930	19.859008	12.433340
H	-1.291070	15.883735	12.963753
H	-2.196922	16.271410	11.482359
H	-3.054635	16.093356	13.027620
C	-1.769733	11.563310	12.258017
C	-1.929730	13.073471	11.998505
P	-0.986279	11.303300	13.916060
C	-3.123994	10.833643	12.162700
C	-0.789811	10.991056	11.212176
H	-0.957904	13.581956	12.027735
H	-2.604085	13.557773	12.715399
H	-2.357892	13.226212	10.995954
N	-2.156939	11.617162	15.030322
O	0.304646	12.120686	13.955893
N	-0.616660	9.721309	14.230902
H	-3.881417	11.271166	12.826466
H	-3.028931	9.765960	12.405234
H	-3.506857	10.907107	11.133268
H	0.208664	11.440019	11.296896
H	-1.172082	11.215782	10.205526
H	-0.689312	9.903210	11.291403
C	-1.912526	11.631410	16.493973
H	-2.920331	12.240686	14.739082
C	0.569877	8.984599	13.744808
H	-1.369775	9.139037	14.593809
H	-0.945477	11.133805	16.655773
C	-1.831490	13.069263	17.015812
C	-3.009803	10.830171	17.201302
C	0.120765	7.781694	12.915304

C	1.451819	8.547574	14.917125
H	1.139520	9.679725	13.112628
H	-1.021349	13.616596	16.520172
H	-1.647926	13.081268	18.099021
H	-2.771789	13.603778	16.825912
H	-4.000939	11.218456	16.929966
H	-2.907753	10.908243	18.291765
H	-2.971021	9.769118	16.927446
H	0.983488	7.251955	12.498704
H	-0.434574	7.076574	13.550574
H	-0.530754	8.078464	12.085172
H	0.911775	7.821342	15.537032
H	2.378828	8.080299	14.557314
H	1.720745	9.409446	15.541104
I	8.540229	14.051617	14.593511
I	5.389293	18.602639	16.017325
I	4.833728	9.601471	13.030545

Molecule 8: Geometry from the X-ray measurement

Dy	4.35680	0.16979	4.27075
Br	4.35680	-2.68165	4.27075
O	5.18808	0.62347	2.32585
N	5.75969	2.28628	4.71662
N	6.60229	-0.26949	5.49389
O	3.52552	0.62347	6.21565
N	2.95391	2.28628	3.82488
N	2.11131	-0.26949	3.04761
C	5.64467	1.66908	1.63826
C	6.39317	2.09330	6.05080
C	4.92318	3.52244	4.77470
C	6.87242	2.44102	3.71897
C	7.25233	-1.45001	5.58272
C	7.14515	0.78586	6.12596
C	3.06893	1.66908	6.90324
C	2.32043	2.09330	2.49070
C	3.79042	3.52244	3.76680
C	1.84118	2.44102	4.82253
C	1.46127	-1.45001	2.95878
C	1.56845	0.78586	2.41554
C	6.45591	2.63923	2.30108
C	5.35364	1.84468	0.27675
H	5.69347	2.10721	6.75120
H	7.01793	2.84090	6.22846
H	5.50002	4.31004	4.61070
H	4.54327	3.61111	5.68522
H	7.42747	3.21471	3.99059
H	7.44577	1.63430	3.76509
H	6.86632	-2.21152	5.16590
C	8.45742	-1.60301	6.25409
C	8.33979	0.69197	6.84516
C	2.25769	2.63923	6.24042
C	3.35996	1.84468	8.26475
H	3.02013	2.10721	1.79030
H	1.69567	2.84090	2.31304
H	3.21358	4.31004	3.93080
H	4.17033	3.61111	2.85628
H	1.28613	3.21471	4.55091
H	1.26783	1.63430	4.77641
H	1.84728	-2.21152	3.37560
C	0.25618	-1.60301	2.28741
C	0.37381	0.69197	1.69634
C	6.91686	3.73803	1.57164
C	5.84595	2.95565	-0.42195
H	4.81862	1.20486	-0.17596
H	8.89833	-2.44450	6.26604
C	9.00463	-0.51637	6.89982
H	8.69094	1.45175	7.29444
C	1.79674	3.73803	6.96986
C	2.86765	2.95565	8.96345
H	3.89498	1.20486	8.71745

H	-0.18473	-2.44450	2.27546
C	-0.29103	-0.51637	1.64168
H	0.02266	1.45175	1.24706
H	7.43444	4.40219	2.01238
C	6.63715	3.88755	0.21525
H	5.63247	3.06866	-1.34102
H	9.82458	-0.59808	7.37302
H	1.27916	4.40219	6.52912
C	2.07645	3.88755	8.32625
H	3.08113	3.06866	9.88251
H	-1.11098	-0.59808	1.16848
H	6.99005	4.62473	-0.26649
H	1.72355	4.62473	8.80800

Molecule 8: Optimized Geometry

Dy	4.2708216366	0.1691818223	4.2237701665
Br	4.2765740166	-2.7147727353	4.2213326333
O	5.0628076701	0.6031000247	2.2478155228
N	5.6753393428	2.2817090148	4.6638550272
N	6.5193855208	-0.2789307928	5.4329150208
O	3.4717729346	0.6122685389	6.1927610510
N	2.8658424825	2.2858193569	3.7713577386
N	2.0306285309	-0.2975782941	3.0259023863
C	5.5140054690	1.6608227419	1.5687360139
C	6.3256650660	2.0907636366	5.9856909214
C	4.8421049124	3.5169157175	4.7198880423
C	6.7657839757	2.4127792250	3.6374237228
C	7.1691200166	-1.4615250900	5.5111793158
C	7.0634897898	0.7828325567	6.0781629878
C	3.0274261292	1.6754746772	6.8676850090
C	2.2179173127	2.0741653314	2.4533739289
C	3.7020953232	3.5163690924	3.7031433249
C	1.7774729808	2.4255489234	4.7966472793
C	1.3787815624	-1.4798332893	2.9533038220
C	1.4859534944	0.7614596140	2.3744767061
C	6.3271877179	2.6315440533	2.2179576205
C	5.1876087742	1.8583892588	0.2076260420
H	5.5334515605	2.0984013970	6.7507929831
H	7.0188786360	2.9194331859	6.2112188867
H	5.4751107769	4.4060469724	4.5562583900
H	4.4274109784	3.5915131337	5.7302360597
H	7.4270091971	3.2406168515	3.9373851702
H	7.3453740045	1.4808966465	3.6899708184
H	6.6869467729	-2.2892876287	4.9966390562
C	8.3654797308	-1.6315678411	6.2033519342
C	8.2446571862	0.6828013241	6.8123813682
C	2.2183236194	2.6475600815	6.2152525101
C	3.3559537701	1.8758945765	8.2278046269
H	3.0097988809	2.0765917823	1.6879771425
H	1.5189864767	2.8947422557	2.2151741143
H	3.0751363506	4.4109193563	3.8616181477
H	4.1159894316	3.5826786686	2.6918679434
H	1.1177121337	3.2541020779	4.4949244366
H	1.1949013948	1.4951122560	4.7491671168
H	1.8598222723	-2.3052568438	3.4722674291
C	0.1835348195	-1.6538453548	2.2603213523
C	0.3050275135	0.6562182110	1.6400854458
C	6.7892414005	3.7420920248	1.5038610306
C	5.6675378522	2.9729277173	-0.4892659590
H	4.5797544976	1.1158625612	-0.3071669979
H	8.8472289615	-2.6058754299	6.2335698455
C	8.9211070505	-0.5364877438	6.8603208272
H	8.6412373550	1.5614802669	7.3155040561
C	1.7617635608	3.7621376151	6.9263910040
C	2.8820819447	2.9952488141	8.9211827395

H	3.9600198268	1.1312543921	8.7442163578
H	-0.2974125467	-2.6285201167	2.2348200448
C	-0.3713675161	-0.5629257064	1.5960759092
H	-0.0910659917	1.5326577433	1.1326371986
H	7.4138832798	4.4744344556	2.0156246642
C	6.4734066987	3.9171572250	0.1531958690
H	5.4073448792	3.0841234463	-1.5428763771
H	9.8676042258	-0.6305199516	7.3899765156
H	1.1386929671	4.4946513280	6.4130994841
C	2.0800231562	3.9407909349	8.2760305838
H	3.1434521041	3.1089136397	9.9742075223
H	-1.3165316639	-0.6596645146	1.0643360824
H	6.8673120239	4.7787132723	-0.3772596257
H	1.6906102116	4.8060747026	8.8036098300

Molecule 9: Geometry from the X-ray measurement

Dy	4.351550	0.126947	4.248150
Cl	4.351550	-2.554927	4.248150
O	5.196621	0.609911	2.312693
N	5.763193	2.248941	4.695055
N	6.569970	-0.319559	5.486911
O	3.506479	0.609911	6.183607
N	2.939907	2.248941	3.801245
N	2.133130	-0.319559	3.009389
C	5.662237	1.651056	1.627891
C	6.391557	2.058236	6.052764
C	4.923344	3.480790	4.768124
C	6.871968	2.401848	3.719680
C	7.206167	-1.492994	5.580370
C	7.115655	0.742202	6.113937
C	3.040863	1.651056	6.868409
C	2.311543	2.058236	2.443536
C	3.779756	3.480790	3.728176
C	1.831132	2.401848	4.776620
C	1.496933	-1.492994	2.915930
C	1.587445	0.742202	2.382363
C	6.471625	2.628632	2.295700
C	5.375905	1.843478	0.282077
H	7.027753	2.795284	6.229487
H	5.689216	2.082289	6.749461
H	5.497748	4.274533	4.618589
H	4.535185	3.554666	5.677228
H	7.432447	1.585769	3.758763
H	7.436799	3.161230	4.008554
H	6.805824	-2.252377	5.174247
C	8.409806	-1.666518	6.234585
C	8.323645	0.625374	6.793641
C	2.231475	2.628632	6.200600
C	3.327195	1.843478	8.214223
H	1.675347	2.795284	2.266813
H	3.013884	2.082289	1.746839

H	3.205352	4.274533	3.877711
H	4.167915	3.554666	2.819072
H	1.270653	1.585769	4.737537
H	1.266301	3.161230	4.487746
H	1.897276	-2.252377	3.322053
C	0.293294	-1.666518	2.261715
C	0.379455	0.625374	1.702659
C	6.951166	3.716164	1.559921
C	5.865889	2.941319	-0.407822
H	4.835442	1.209514	-0.175024
H	8.841479	-2.513522	6.246480
C	8.970285	-0.570396	6.870108
H	8.702230	1.391629	7.211659
C	1.751934	3.716164	6.936379
C	2.837211	2.941319	8.904122
H	3.867658	1.209514	8.671324
H	-0.138379	-2.513522	2.249820
C	-0.267185	-0.570396	1.626192
H	0.000870	1.391629	1.284641
H	7.490758	4.369027	1.993232
C	6.659612	3.862199	0.224302
H	5.649182	3.056429	-1.325423
H	9.786636	-0.652863	7.349300
H	1.212342	4.369027	6.503068
C	2.043488	3.862199	8.271998
H	3.053918	3.056429	9.821723
H	-1.083536	-0.652863	1.147001
H	7.007736	4.600965	-0.259987
H	1.695364	4.600965	8.756287

Molecule 9: Optimized Geometry

Dy	4.2859000849	0.0878420797	4.2122146463
Cl	4.2861766970	-2.5927222108	4.2018717484
O	5.1039070450	0.5497848380	2.2441064834
N	5.6923389726	2.2248851750	4.6599283927
N	6.5056797441	-0.3534655819	5.4392910813
O	3.4661720610	0.5578254013	6.1767403745
N	2.8852765827	2.2255459291	3.7532230605
N	2.0604037558	-0.3517907319	2.9917485491
C	5.5496982950	1.6155365101	1.5761286385
C	6.3287034289	2.0188553790	5.9841273568
C	4.8554013431	3.4539286821	4.7171188456
C	6.7941293047	2.3652993307	3.6503908677
C	7.1426575370	-1.5413887667	5.5250348201
C	7.0655744942	0.7105771365	6.0667117250
C	3.0231187286	1.6275228791	6.8405328964
C	2.2477853553	2.0157654015	2.4300425412
C	3.7251930587	3.4524062837	3.6900152307
C	1.7839491435	2.3748017207	4.7620310264
C	1.4195762034	-1.5380434809	2.9118228164
C	1.5084865322	0.7085195835	2.3512921747
C	6.3665223619	2.5821791188	2.2286077313
C	5.2143870219	1.8304696416	0.2188889281
H	7.0202691637	2.8438507913	6.2276810686
H	5.5297348428	2.0152992570	6.7418263404
H	5.4825092462	4.3493943766	4.5664568838
H	4.4300312441	3.5181099349	5.7235937868
H	7.3805163287	1.4376305011	3.7087042002
H	7.4465328121	3.1986555791	3.9581951551
H	6.6423680901	-2.3692878420	5.0280411776
C	8.3466606839	-1.7127315417	6.2043356613
C	8.2595551197	0.6116416747	6.7780924622
C	2.2114777839	2.5952283964	6.1833715718
C	3.3560956968	1.8441643068	8.1977970084
H	1.5572894202	2.8410730953	2.1844377994
H	3.0462019531	2.0090961891	1.6717865434
H	3.1000456022	4.3499771888	3.8361085765
H	4.1505981090	3.5108423108	2.6832634800
H	1.1935068742	1.4495647467	4.7069159490
H	1.1351756877	3.2098039794	4.4508599336
H	1.9134256623	-2.3632568611	3.4195166491
C	0.2204377999	-1.7115030786	2.2245340992

C	0.3204172969	0.6070292774	1.6298131312
C	6.8353327668	3.6925816663	1.5197183686
C	5.6968871299	2.9476621165	-0.4721566318
H	4.6007263943	1.0963473911	-0.3005317188
H	8.8161003853	-2.6916684707	6.2501624203
C	8.9256544426	-0.6126407079	6.8319585049
H	8.6707560723	1.4935786567	7.2628851187
C	1.7453733119	3.7093875454	6.8882067113
C	2.8758779908	2.9647604050	8.8849079484
H	3.9645721335	1.1076980340	8.7200596890
H	-0.2526474054	-2.6888919700	2.1849971310
C	-0.3490768518	-0.6156559700	1.5808756770
H	-0.0838354763	1.4860516447	1.1339907581
H	7.4683646791	4.4158379117	2.0348594709
C	6.5158277830	3.8797936313	0.1715366944
H	5.4316432022	3.0675227824	-1.5238102752
H	9.8793072302	-0.7064898102	7.3485093582
H	1.1159885740	4.4336647698	6.3700715155
C	2.0624897043	3.8984774946	8.2366229823
H	3.1382061439	3.0859083771	9.9371060994
H	-1.2987194145	-0.7116751394	1.0571877857
H	6.9155005768	4.7414443706	-0.3535565955
H	1.6647056860	4.7628382973	8.7585845976

Molecule 10: Geometry from the X-ray measurement

C	8.815600	6.273560	-9.972870
C	9.349960	6.106250	-8.690580
C	9.146810	7.446690	-10.700710
N	7.996840	5.323330	-10.573310
C	10.215930	7.075520	-8.173890
C	9.062470	4.913890	-7.890780
C	10.001850	8.375580	-10.097910
C	8.599800	7.675550	-12.105680
Dy	6.946400	4.623820	-12.444690
H	7.878720	4.596570	-10.130940
H	10.583950	6.954360	-7.328080
C	10.531720	8.200570	-8.885230
C	7.768290	4.856190	-7.138160
C	9.869610	3.677300	-8.113730
H	9.635880	5.149650	-7.018700
H	10.210700	9.152530	-10.563870
C	9.532270	7.125520	-13.100130
H	7.762170	7.171680	-12.178820
C	8.287970	9.123690	-12.447780
N	6.549940	5.592760	-14.360000
H	5.776170	5.290830	-14.580600
N	5.720210	2.836690	-12.892510
O	8.894390	3.268430	-12.793420
O	5.000560	5.623730	-11.465130
H	11.108730	8.835210	-8.527790
H	7.313790	3.904510	-6.957510
H	6.811860	5.305660	-7.305860
H	8.380030	5.435720	-6.478750
H	10.560860	3.661830	-8.930330
H	9.566420	2.668060	-8.299250
H	10.102340	3.847000	-7.083230
H	9.190630	7.285150	-13.982510
H	9.628430	6.181250	-12.960930
H	10.387850	7.552460	-13.005760
H	9.106970	9.621780	-12.504410
H	7.730790	9.502550	-11.764760
H	7.830850	9.162150	-13.291240
C	6.884830	6.442800	-15.422870
C	4.933630	1.727210	-12.574010
H	5.738420	2.809950	-13.751300
C	8.882440	1.990690	-13.478800
C	10.237890	3.477290	-12.275550
C	3.621810	5.219670	-11.630280
C	5.019600	6.646660	-10.476580
C	6.181190	7.644780	-15.605720
C	7.906940	6.127400	-16.320590
C	4.287700	0.944490	-13.562560
C	4.776520	1.371430	-11.223300
H	8.749840	2.119540	-14.430780
H	8.165220	1.432970	-13.140240
C	10.207270	1.348350	-13.220460
C	10.785540	2.115690	-12.045520
H	10.212370	3.979230	-11.447440

H	10.781980	3.960000	-12.918460
H	3.242390	5.637000	-12.420650
H	3.562970	4.258090	-11.730550
C	2.910590	5.644690	-10.451810
C	3.876520	6.510110	-9.707450
H	5.809780	6.560110	-9.919780
H	5.036650	7.519770	-10.898900
C	6.532080	8.502510	-16.615500
C	5.016910	8.044790	-14.685590
C	8.236970	7.046670	-17.317400
C	8.612140	4.817730	-16.131840
C	3.535940	-0.149790	-13.176810
C	4.412820	1.357970	-15.019430
C	3.996510	0.267530	-10.891820
C	5.480790	2.177230	-10.135660
H	10.783580	1.421430	-13.997850
H	10.097040	0.409850	-12.998680
H	10.482590	1.750290	-11.199710
H	11.755450	2.115690	-12.065570
H	2.648470	4.881200	-9.913890
H	2.114980	6.146630	-10.691280
H	3.481900	7.379380	-9.538760
H	4.096740	6.104330	-8.854550
H	6.066270	9.300620	-16.714590
C	7.555600	8.214030	-17.484910
C	4.764260	9.529470	-14.585320
H	5.242750	7.729400	-13.785510
C	3.754120	7.244760	-15.145650
H	8.946730	6.856280	-17.887170
C	10.057140	4.923510	-16.470400
H	8.537960	4.567720	-15.186940
C	7.998630	3.717680	-16.954060
H	3.117050	-0.667120	-13.826800
C	3.393740	-0.486340	-11.875650
C	3.584390	2.577250	-15.323780
H	5.349800	1.596440	-15.175150
C	4.049280	0.232920	-16.020960
H	3.881370	0.034830	-9.998820
C	4.731460	2.225310	-8.790850

H	5.558120	3.098430	-10.458890
C	6.889860	1.659900	-9.909170
H	7.777740	8.800600	-18.170290
H	5.566420	9.973720	-14.301020
H	4.065340	9.692940	-13.947120
H	4.498400	9.867950	-15.444110
H	3.499190	7.529390	-16.026860
H	3.031770	7.410150	-14.534590
H	3.954740	6.306260	-15.157450
H	10.464110	4.056160	-16.407880
H	10.487720	5.523530	-15.856980
H	10.153160	5.256210	-17.364580
H	8.000620	3.971540	-17.880090
H	7.096000	3.565750	-16.666230
H	8.509200	2.911880	-16.842000
H	2.886480	-1.230610	-11.643260
H	2.653190	2.346470	-15.302550
H	3.811990	2.909960	-16.196730
H	3.760440	3.256130	-14.669070
H	3.111750	0.275230	-16.221500
H	4.256510	-0.619040	-15.632850
H	4.555570	0.348310	-16.829020
H	3.828030	2.509940	-8.939490
H	5.170970	2.842650	-8.202210
H	4.730670	1.350270	-8.394490
H	6.851060	0.763710	-9.564710
H	7.340800	2.225310	-9.278050
H	7.368370	1.661830	-10.740820

Molecule 10: Optimized Geometry

C	9.0491252940	6.1667318195	-9.3439185540
C	9.6227091031	5.9879566015	-8.0412210327
C	9.4014233074	7.3405315989	-10.0826943040
N	8.1811095636	5.2404477771	-9.9104495531
C	10.5427624642	6.9240961376	-7.5583810963
C	9.2952253729	4.8074400654	-7.1316696911
C	10.3342074475	8.2401295631	-9.5561281420
C	8.8283330858	7.5720242640	-11.4703847063
Dy	7.1320841563	4.5028762332	-11.7754970107
H	8.0215476373	4.4947584460	-9.2284682182
H	10.9851092993	6.7675885342	-6.5746463381
C	10.9235791898	8.0385533312	-8.3073893545
C	7.8094476002	4.7416913835	-6.7249601039
C	9.8301299788	3.4548630134	-7.6510800775
H	9.8475879134	5.0046248886	-6.1990666095
H	10.6125189639	9.1157510349	-10.1399447602
C	9.7283769524	6.9224895619	-12.5413601926
H	7.8345859344	7.0837584160	-11.5042347955
C	8.5828546297	9.0503432662	-11.8156647605
N	6.5274709215	5.3993514872	-13.7415396169
H	5.5868351410	5.6924900666	-13.4593869750
N	5.8946660490	2.7158351831	-12.2092470546
O	9.0896912407	3.2073419652	-12.1355266865
O	5.1392040864	5.4859830270	-10.8657945861
H	11.6711448177	8.7284194902	-7.9221251715
H	7.6399260050	3.9241415678	-6.0126171809
H	7.1259734097	4.5809121080	-7.5696184988
H	7.5015016052	5.6792256490	-6.2418840142
H	10.9185874959	3.4995848466	-7.7897472561
H	9.3923548115	3.1534706295	-8.6110408175
H	9.6219822161	2.6526189675	-6.9279005643
H	9.3089946775	7.0344771052	-13.5493256014
H	9.8846967942	5.8529613370	-12.3484961137
H	10.7209604013	7.3949240574	-12.5249438897
H	9.5258725957	9.6061962961	-11.8995633063
H	7.9753699933	9.5498372487	-11.0506752538
H	8.0731472189	9.1322165882	-12.7858238298
C	6.9942838531	6.2820326395	-14.7068861040
C	5.0595029553	1.6463658435	-11.9437825976
H	5.8903998017	2.8866435213	-13.2220339225
C	9.0633101227	1.8913336917	-12.8119628372
C	10.4376072271	3.4161885163	-11.5812652092
C	3.7533050430	5.0577027724	-11.1043299314
C	5.0749111081	6.4655805452	-9.7749414213
C	6.4191321362	7.5770788665	-14.8777523415
C	8.0970249063	5.8974058925	-15.5264347259
C	4.4011972683	0.9426214140	-13.0064145692
C	4.8269449763	1.2373018744	-10.5968540730
H	8.8623749572	2.0732890362	-13.8750923649
H	8.2216926772	1.3375520567	-12.3817957586
C	10.4247121064	1.2525067435	-12.5452798234
C	10.9559256794	2.0168364744	-11.3231450463

H	10.3109011737	4.0559062587	-10.7022055264
H	11.0412484978	3.9369267144	-12.3399676445
H	3.3288229825	5.7054089301	-11.8859823875
H	3.8120430101	4.0282834095	-11.4729804901
C	3.0215985831	5.2203881942	-9.7575840800
C	4.0670844648	5.8591894311	-8.8132517901
H	6.0934212180	6.5694124221	-9.3909314379
H	4.7270668730	7.4240694704	-10.1867513980
C	7.0109898067	8.4670762785	-15.7768435412
C	5.1696305458	7.9428835529	-14.0923403172
C	8.6488818071	6.8238095634	-16.4155717364
C	8.5997255352	4.4669219406	-15.4340237471
C	3.6244797791	-0.1782875330	-12.7046514900
C	4.5721192041	1.4406539569	-14.4348343293
C	4.0159338234	0.1234723534	-10.3524324494
C	5.4823805861	1.9972240946	-9.4550346918
H	11.1023991607	1.4019478671	-13.3933759216
H	10.3404256111	0.1755602646	-12.3598820719
H	10.5373821327	1.6076922722	-10.3937367588
H	12.0504420500	1.9745635595	-11.2553182735
H	2.6762020204	4.2541063065	-9.3738275551
H	2.1452031333	5.8717234031	-9.8603776006
H	3.6202874165	6.6065403093	-8.1472460785
H	4.5555428821	5.0950977661	-8.1949337920
H	6.5967069970	9.4644973327	-15.8750095621
C	8.1242103752	8.1130826403	-16.5396816671
C	4.9036561952	9.4507952306	-13.9893618682
H	5.3119569536	7.5756633651	-13.0602022011
C	3.9364132313	7.2222760543	-14.6932688226
H	9.5080372492	6.5460079980	-17.0229839206
C	10.0418958610	4.2676252509	-15.9034975580
H	8.5644687654	4.1837565266	-14.3726991505
C	7.6401699323	3.5275372012	-16.1952549945
H	3.1542628996	-0.7311672916	-13.5146206027
C	3.4229512664	-0.6010148283	-11.3883743878
C	3.7935049889	2.7573828363	-14.6686358095
H	5.6463124375	1.6535057302	-14.5823900488
C	4.1905843174	0.4203973024	-15.5120826139
H	3.8274438747	-0.1789208786	-9.3251401610
C	4.6545132763	2.0086428230	-8.1620213301

H	5.5752393329	3.0471833717	-9.7825132412
C	6.9002821699	1.4583032425	-9.1767647218
H	8.5827752604	8.8249355886	-17.2207042204
H	5.7832827275	9.9925545767	-13.6177150980
H	4.0705097887	9.6517800390	-13.3023434978
H	4.6297954571	9.8806348600	-14.9623860104
H	3.7489443515	7.5989214234	-15.7090152644
H	3.0302111319	7.3922679170	-14.0942056524
H	4.0889023497	6.1374091830	-14.7823656445
H	10.3598351429	3.2274008399	-15.7436148040
H	10.7429605455	4.9252513829	-15.3710977530
H	10.1475432500	4.4705562428	-16.9772446841
H	7.6594604119	3.7568845700	-17.2696839492
H	6.6105944185	3.6539343645	-15.8359881941
H	7.9199778063	2.4705690752	-16.0747264812
H	2.7753108515	-1.4489649413	-11.1721874925
H	2.7130998561	2.5607398839	-14.6978670563
H	4.0775188084	3.2156648661	-15.6260011315
H	3.9752145913	3.5003346111	-13.8817139076
H	3.1191190414	0.1880099673	-15.4862642952
H	4.7526118807	-0.5153228871	-15.3976958028
H	4.4095991000	0.8281382603	-16.5077997571
H	3.6302409077	2.3652347057	-8.3372635808
H	5.1197491606	2.6588357427	-7.4084558571
H	4.5826859545	1.0090330235	-7.7150640295
H	6.8556122343	0.3966870391	-8.8947276408
H	7.3830239960	2.0038756874	-8.3543361893
H	7.5433991310	1.5334720474	-10.0650637148

Molecule 11: Geometry from the X-ray measurement

C	7.643280	2.756954	2.847639
N	8.055764	2.199217	4.024490
N	6.468737	2.130412	2.574669
Dy	8.107811	4.680111	1.188959
C	7.158481	1.249714	4.469170
C	9.271976	2.545767	4.748812
C	6.168616	1.214610	3.559990
B	5.712192	2.443262	1.235920
C	7.697759	2.624681	-0.300640
C	10.004768	5.240797	2.847639
C	10.092080	5.354114	-0.300640
C	6.672953	6.043984	2.847639
C	6.531161	6.062941	-0.300640
H	7.226580	0.727362	5.259232
H	9.502215	1.821213	5.367164
H	9.125239	3.372825	5.255307
H	10.004849	2.677759	4.113190
H	5.398451	0.658557	3.591192
N	6.594639	1.929475	0.064367
H	5.616530	3.510434	1.148004
H	4.777455	1.975672	1.226500
N	8.288678	1.859266	-1.261431
N	10.281541	5.876888	4.024490
N	11.134640	4.536885	2.574669
N	11.245706	4.746388	0.064367
N	10.459489	6.248572	-1.261431
N	5.983696	5.965631	4.024490
N	6.717622	7.374439	2.574669
N	6.480655	7.365873	0.064367
N	5.572833	5.933897	-1.261431
C	6.519244	0.739297	-0.636014
C	9.528157	2.186298	-1.940225
C	7.571938	0.696470	-1.474744
C	11.552475	5.574569	4.469170
C	9.373313	6.756883	4.748812
C	12.077809	4.734873	3.559990
B	11.241977	3.725273	1.235920
C	12.314128	5.276182	-0.636014
C	9.556532	7.158477	-1.940225
C	11.824870	6.209256	-1.474744
C	5.610044	7.217452	4.469170
C	5.675711	4.739086	4.748812
C	6.074575	8.092252	3.559990
B	7.366831	7.873201	1.235920
C	5.487628	8.026256	-0.636014
C	5.236311	4.696961	-1.940225
C	4.924192	7.136010	-1.474744
H	5.847579	0.074421	-0.541622
H	9.433305	2.007968	-2.898465
H	10.255355	1.637266	-1.575807

H	9.736507	3.134115	-1.803446
H	7.781909	0.004213	-2.089956
H	11.970796	5.894721	5.259232
H	9.885676	7.318553	5.367164
H	8.730428	6.216276	5.255307
H	8.892568	7.325574	4.113190
H	12.944447	4.345917	3.591192
H	10.365610	3.108840	1.148004
H	12.114290	3.149561	1.226500
H	13.225760	5.026941	-0.541622
H	9.758396	7.165498	-2.898465
H	9.668408	8.062765	-1.575807
H	8.631523	6.865005	-1.803446
H	12.319397	6.737225	-2.089956
H	5.123624	7.419653	5.259232
H	4.933110	4.901970	5.367164
H	6.465333	4.452634	5.255307
H	5.423583	4.038403	4.113190
H	5.978102	9.037261	3.591192
H	8.338860	7.422462	1.148004
H	7.429255	8.916502	1.226500
H	5.247661	8.940373	-0.541622
H	5.129299	4.868270	-2.898465
H	4.397237	4.341705	-1.575807
H	5.952970	4.042616	-1.803446
H	4.219694	7.300298	-2.089956

Molecule 11: Optimized Geometry

C	7.5010735485	2.7592763870	2.7870762621
N	7.9037534050	2.1911756337	3.9687379395
N	6.3128683750	2.1393412888	2.5093151727
Dy	8.0205392116	4.6326547598	1.1394675109
C	6.9849889355	1.2509672131	4.4229814732
C	9.0980337734	2.5581488211	4.7194430151
C	5.9824297595	1.2229153007	3.4913322716
B	5.5729188440	2.4370043689	1.1640637505
C	7.6004507851	2.5957362083	-0.3385884437
C	9.8871206635	5.1231442541	2.8017462943
C	9.9814962785	5.2764596813	-0.3405483100
C	6.6546181753	5.9960931012	2.7854094474
C	6.4909619436	6.0103124097	-0.3252230010
H	7.1479051726	0.6817642964	5.3284940635
H	9.3385771964	1.7584925425	5.4271038134
H	8.9390322429	3.4890439932	5.2798770720
H	9.9403969024	2.7038402722	4.0393970764
H	5.0796668850	0.6290802131	3.4507221903
N	6.4735946132	1.9055929636	0.0127178521
H	5.4884488622	3.6533338433	1.0485988520
H	4.4811806810	1.9152073450	1.1503721161
N	8.2047145779	1.8014365753	-1.2772922635
N	10.1715694763	5.7760050687	3.9732915509
N	11.0293304160	4.4235162251	2.5251268634
N	11.1519253767	4.6741448551	0.0273327832
N	10.3552782188	6.1994633777	-1.2816059302
N	5.9622362795	5.9204016878	3.9675328462
N	6.7206243260	7.3377589455	2.5251173233
N	6.4418119954	7.3294067247	0.0285116490
N	5.5103335411	5.8736439760	-1.2720847645
C	6.3998437971	0.7017726985	-0.6694411403
C	9.4725559797	2.1062337462	-1.9226138755
C	7.4841932260	0.6335245132	-1.4951717715
C	11.4548600841	5.4826688072	4.4212212958
C	9.2483381811	6.6236421698	4.7185380129
C	11.9922236571	4.6274434567	3.4970528804
B	11.1389591148	3.6315406866	1.1826551754
C	12.2268205033	5.2327012903	-0.6453877368
C	9.4427333884	7.1186403520	-1.9459710596
C	11.7301394980	6.1891761283	-1.4833264502
C	5.6175580771	7.1824252187	4.4380443412
C	5.6588807358	4.6963977259	4.6997831969
C	6.0994796862	8.0733684620	3.5172168331
B	7.3458099189	7.8459627041	1.1848839722
C	5.4328023226	7.9854676116	-0.6575993711
C	5.1651674710	4.6215217295	-1.9305698261
C	4.8464349493	7.0746655421	-1.4881710172
H	5.6139305516	-0.0228188982	-0.5045871019
H	9.4271114403	1.8510582951	-2.9860226060
H	10.2891165992	1.5361178531	-1.4596586065
H	9.6737418548	3.1743340167	-1.7968236518
H	7.8134459849	-0.1333871910	-2.1816653789
H	11.8611550372	5.9284737603	5.3190628437

H	9.8079402660	7.1838024478	5.4743375896
H	8.4818392781	6.0194806706	5.2203997609
H	8.7483738975	7.3253028728	4.0464836065
H	12.9669520533	4.1609111157	3.4562369096
H	10.1213322963	2.9586842477	1.0670015318
H	12.1275000298	2.9367342831	1.1686930153
H	13.2520353560	4.9329445705	-0.4731366880
H	9.7307137710	7.2442268746	-2.9944352810
H	9.4577192990	8.1010921475	-1.4555838067
H	8.4302269474	6.7098743405	-1.8774633992
H	12.2220284807	6.8651875232	-2.1686304229
H	5.0448964129	7.3163594003	5.3458150383
H	4.8541820162	4.8962679063	5.4146755139
H	6.5389384325	4.3390857064	5.2503122311
H	5.3424181919	3.9116390902	4.0089570700
H	6.0429874821	9.1527165358	3.4887934106
H	8.4468605981	7.3239162078	1.0659457915
H	7.4243108997	9.0529868639	1.1811986651
H	5.1894824863	9.0274110136	-0.4979446922
H	4.9403868445	4.8004312483	-2.9868176550
H	4.2867258475	4.1644910371	-1.4560382177
H	6.0132180185	3.9371088767	-1.8338009628
H	4.0210081403	7.1677995155	-2.1785355477

Molecule 12: Geometry from the X-ray measurement

Dy	9.556044	2.129450	14.226799
C	7.814594	3.997071	14.041436
C	7.200727	2.965486	14.838974
C	6.938404	1.876663	13.965859
C	7.361681	2.171775	12.655192
C	7.921230	3.469282	12.716847
C	9.878547	4.970581	15.031894
H	10.418108	4.481664	14.375368
C	11.309863	0.269903	14.059336
C	11.901111	1.272952	14.910573
C	12.181399	2.391312	14.077236
C	11.789214	2.143232	12.756625
C	11.242658	0.830959	12.754636
C	9.222604	-0.715259	14.958306
H	8.705729	-0.207899	14.298180
C	8.503646	5.313898	14.413355
C	6.723208	2.906035	16.310740
H	6.531568	1.059770	14.229882
C	6.950427	1.380720	11.412147
H	8.312761	3.926099	11.980706
H	9.749413	4.415173	15.827781
H	10.339512	5.798664	15.281140
C	10.609173	-1.066309	14.369600
C	12.344825	1.283796	16.390293
H	12.582383	3.198662	14.375984
C	12.213807	2.961852	11.525513
H	10.883314	0.393475	11.991466
H	9.338013	-0.174634	15.767936
H	8.744044	-1.540740	15.183128
C	7.687535	6.218609	15.334203
C	8.787666	6.155878	13.152410
C	6.296225	1.497290	16.700558
C	5.484590	3.792792	16.483770
C	7.815513	3.303655	17.317108
C	5.445037	1.282940	11.268949
C	7.388342	2.232269	10.202913
C	6.745838	-0.112075	11.766167
C	10.356125	-1.873578	13.098711
C	11.388837	-1.989051	15.300392
C	13.586277	0.384403	16.537472
C	12.791193	2.697941	16.809946
C	11.241718	0.894893	17.376774
C	13.524994	2.371619	11.032273
C	12.475588	4.435982	11.907377
C	11.159630	2.944490	10.455500

H	7.510011	5.752329	16.177664
H	6.837282	6.443967	14.900887
H	8.189837	7.040229	15.514196
H	9.391097	5.661020	12.558016
H	9.207886	7.002180	13.411720
H	7.946630	6.338322	12.683971
H	5.572673	1.199676	16.108768
H	5.980750	1.495641	17.627630
H	7.059213	0.888399	16.613127
H	5.710639	4.717318	16.251671
H	5.183826	3.753919	17.415895
H	4.769248	3.473009	15.895940
H	8.569193	2.680835	17.244694
H	7.450916	3.270548	18.225903
H	8.124358	4.213174	17.121820
H	7.649798	-0.525334	11.765371
H	5.103709	0.584390	11.863621
H	5.216944	1.065438	10.341518
H	5.038040	2.143853	11.510318
H	6.872254	3.067183	10.191398
H	7.216110	1.733820	9.375940
H	8.340281	2.435312	10.273280
H	6.499475	-0.608765	10.958526
H	6.029816	-0.193875	12.431066
H	9.946109	-2.732191	13.332960
H	11.207101	-2.032895	12.638725
H	9.754502	-1.374061	12.508434
H	11.514218	-1.548549	16.167759
H	12.263963	-2.187113	14.906337
H	10.890915	-2.823034	15.427302
H	14.269858	0.662100	15.891604
H	13.943607	0.465758	17.445988
H	13.336492	-0.547943	16.366686
H	12.036365	3.316899	16.724663
H	13.524064	2.993955	16.231184
H	13.096076	2.680385	17.741016
H	10.517681	1.554931	17.333876
H	11.608333	0.871062	18.284595
H	10.890656	0.010539	17.141989
H	13.378725	1.447438	10.740426
H	14.183485	2.384972	11.757515
H	13.859284	2.902245	10.278192
H	11.655478	4.828820	12.273767
H	12.750122	4.936535	11.111350
H	13.186251	4.477696	12.581167
H	11.017158	2.024059	10.151839
H	11.450238	3.497238	9.701021
H	10.321317	3.301810	10.818330

Molecule 12: Optimized Geometry

Dy	9.126600	2.513500	14.046800
C	7.417700	4.383400	13.736400
C	6.778100	3.385500	14.577600
C	6.522500	2.256800	13.736400
C	6.978800	2.492200	12.416000
C	7.545300	3.800700	12.433500
C	9.474600	5.385600	14.732300
C	10.868200	0.663100	13.825300
C	11.460100	1.653800	14.712400
C	11.749500	2.792900	13.893100
C	11.355600	2.571400	12.552400
C	10.794200	1.260600	12.525800
C	8.778000	-0.319900	14.733600
H	8.133700	0.225400	14.010900
C	8.110700	5.714400	14.079400
C	6.326700	3.390000	16.049600
H	5.996300	1.364100	14.053400
C	6.585500	1.668600	11.193400
H	7.931300	4.317500	11.563000
H	10.139900	4.845000	14.028400
H	9.386100	4.822800	15.675200
H	10.042000	6.293700	14.976000
C	10.154700	-0.666700	14.120700
C	11.887700	1.634200	16.197400
H	12.252800	3.687300	14.240200
C	11.755700	3.417700	11.350000
H	10.440300	0.756900	11.633600
H	8.855200	0.243000	15.678900
H	8.187400	-1.215900	14.962800
C	7.284500	6.652900	14.972700
C	8.415900	6.511700	12.795100
C	5.921900	1.968100	16.500800
C	5.067900	4.271200	16.202200
C	7.430800	3.839100	17.027200
C	6.365500	0.187700	11.553000
C	5.248900	2.259600	10.684700
C	7.618600	1.746000	10.061100
C	9.888300	-1.452500	12.818000
C	10.941200	-1.614600	15.040100
C	13.140600	0.742400	16.352900
C	12.298400	3.054200	16.651600
C	10.778000	1.191200	17.179800
C	13.064200	2.810700	10.788800
C	12.014800	4.883100	11.737700
C	10.687600	3.377300	10.254100
H	7.048500	6.248900	15.958600

H	6.337300	6.908400	14.484400
H	7.843500	7.583200	15.137100
H	9.120200	5.988700	12.138700
H	8.879600	7.469700	13.056600
H	7.505800	6.720200	12.220700
H	5.051200	1.590600	15.953100
H	5.652400	1.992800	17.564500
H	6.734600	1.237000	16.384000
H	5.235900	5.309900	15.907500
H	4.722800	4.269900	17.245100
H	4.257000	3.876500	15.576600
H	8.307900	3.168300	16.989500
H	7.059900	3.779000	18.055700
H	7.783500	4.861000	16.873600
H	7.288800	-0.295400	11.896100
H	6.020900	-0.364000	10.667100
H	5.608100	0.056500	12.333200
H	4.487100	2.228600	11.471100
H	4.878000	1.680800	9.829600
H	5.366200	3.305500	10.373400
H	7.247400	1.199700	9.182800
H	8.575300	1.298000	10.355600
H	7.812500	2.777800	9.741900
H	9.396400	-2.402000	13.059600
H	10.820500	-1.679200	12.288500
H	9.227900	-0.919100	12.123500
H	11.132300	-1.213000	16.036800
H	11.908700	-1.866300	14.590700
H	10.375400	-2.545200	15.177000
H	13.954000	1.125700	15.722900
H	13.490400	0.747900	17.394200
H	12.963700	-0.297800	16.066700
H	11.488000	3.786200	16.527600
H	13.177000	3.429600	16.112900
H	12.556900	3.028900	17.717800
H	9.901600	1.860300	17.132400
H	11.146600	1.261400	18.213500
H	10.428700	0.165600	17.033300
H	12.918700	1.769300	10.475800
H	13.854300	2.827500	11.546900
H	13.412600	3.384400	9.919900
H	11.100900	5.396100	12.060700
H	12.405400	5.433700	10.870700
H	12.752500	4.975900	12.540300
H	10.502300	2.357300	9.904800
H	11.013500	3.965000	9.388000
H	9.737400	3.795500	10.604400

References

[1] X.-C. Huang, C. Zhou, D. Shao, and X.-Y. Wang, “Field-induced slow magnetic relaxation in cobalt (ii) compounds with pentagonal bipyramid geometry,” *Inorganic Chemistry*, vol. 53, no. 24, pp. 12671–12673, 2014.

[2] R. Boca, J. Miklovic, and J. Titis, “Simple mononuclear cobalt (ii) complex: A single-molecule magnet showing two slow relaxation processes,” *Inorganic Chemistry*, vol. 53, no. 5, pp. 2367–2369, 2014.

[3] D.-K. Cao, J.-Q. Feng, M. Ren, Y.-W. Gu, Y. Song, and M. D. Ward, “A mononuclear cobalt (ii)–dithienylethene complex showing slow magnetic relaxation and photochromic behavior,” *Chemical Communications*, vol. 49, no. 78, pp. 8863–8865, 2013.

[4] M. R. Saber and K. R. Dunbar, “Ligands effects on the magnetic anisotropy of tetrahedral cobalt complexes,” *Chemical Communications*, vol. 50, no. 82, pp. 12266–12269, 2014.

[5] T. Jurca, A. Farghal, P.-H. Lin, I. Korobkov, M. Murugesu, and D. S. Richeson, “Single-molecule magnet behavior with a single metal center enhanced through peripheral ligand modifications,” *Journal of the American Chemical Society*, vol. 133, no. 40, pp. 15814–15817, 2011.

[6] M. A. Palacios, J. Nehrkorn, E. A. Suturina, E. Ruiz, S. Gómez-Coca, K. Holldack, A. Schnegg, J. Krzystek, J. M. Moreno, and E. Colacio, “Analysis of magnetic anisotropy and the role of magnetic dilution in triggering single-molecule magnet (smm) behavior in a family of coiiyiii dinuclear complexes with easy-plane anisotropy,” *Chemistry–A European Journal*, vol. 23, no. 48, pp. 11649–11661, 2017.

[7] F. Yang, Q. Zhou, Y. Zhang, G. Zeng, G. Li, Z. Shi, B. Wang, and S. Feng, “Inspiration from old molecules: field-induced slow magnetic relaxation in three air-stable tetrahedral cobalt (ii) compounds,” *Chemical Communications*, vol. 49, no. 46, pp. 5289–5291, 2013.

[8] F. Habib, O. R. Luca, V. Vieru, M. Shiddiq, I. Korobkov, S. I. Gorelsky, M. K. Takase, L. F. Chibotaru, S. Hill, R. H. Crabtree, *et al.*, “Influence of the ligand field on slow magnetization relaxation versus spin crossover in mononuclear cobalt complexes,” *Angewandte Chemie International Edition*, vol. 52, no. 43, pp. 11290–11293, 2013.

[9] J. M. Zadrozny, J. Telser, and J. R. Long, “Slow magnetic relaxation in the tetrahedral cobalt (ii) complexes [co (eph) 4] 2-(eo, s, se),” *Polyhedron*, vol. 64, pp. 209–217, 2013.

[10] X.-N. Yao, J.-Z. Du, Y.-Q. Zhang, X.-B. Leng, M.-W. Yang, S.-D. Jiang, Z.-X. Wang, Z.-W. Ouyang, L. Deng, B.-W. Wang, *et al.*, “Two-coordinate co (ii) imido complexes as outstanding single-molecule magnets,” *Journal of the American Chemical Society*, vol. 139, no. 1, pp. 373–380, 2017.

[11] S. Gomez-Coca, E. Cremades, N. Aliaga-Alcalde, and E. Ruiz, “Mononuclear single-molecule magnets: tailoring the magnetic anisotropy of first-row transition-metal complexes,” *Journal of the American Chemical Society*, vol. 135, no. 18, pp. 7010–7018, 2013.

[12] J. Vallejo, I. Castro, R. Ruiz-García, J. Cano, M. Julve, F. Lloret, G. De Munno, W. Wernsdorfer, and E. Pardo, “Field-induced slow magnetic relaxation in a six-coordinate mononuclear cobalt (ii) complex with a positive anisotropy,” *Journal of the American Chemical Society*, vol. 134, no. 38, pp. 15704–15707, 2012.

[13] M. S. Fataftah, J. M. Zadrozny, D. M. Rogers, and D. E. Freedman, “A mononuclear transition metal single-molecule magnet in a nuclear spin-free ligand environment,” *Inorganic chemistry*, vol. 53, no. 19, pp. 10716–10721, 2014.

[14] J. M. Zadrozny and J. R. Long, “Slow magnetic relaxation at zero field in the tetrahedral complex [co (sph) 4] 2–,” *Journal of the American Chemical Society*, vol. 133, no. 51, pp. 20732–20734, 2011.

[15] Y.-Y. Zhu, C. Cui, Y.-Q. Zhang, J.-H. Jia, X. Guo, C. Gao, K. Qian, S.-D. Jiang, B.-W. Wang, Z.-M. Wang, *et al.*, "Zero-field slow magnetic relaxation from single co (ii) ion: a transition metal single-molecule magnet with high anisotropy barrier," *Chemical Science*, vol. 4, no. 4, pp. 1802–1806, 2013.

[16] J. M. Zadrozny, J. Liu, N. A. Piro, C. J. Chang, S. Hill, and J. R. Long, "Slow magnetic relaxation in a pseudotetrahedral cobalt (ii) complex with easy-plane anisotropy," *Chemical Communications*, vol. 48, no. 33, pp. 3927–3929, 2012.

[17] Y. Peng, V. Mereacre, C. E. Anson, Y. Zhang, T. Bodenstein, K. Fink, and A. K. Powell, "Field-induced co (ii) single-ion magnets with mer-directing ligands but ambiguous coordination geometry," *Inorganic Chemistry*, vol. 56, no. 11, pp. 6056–6066, 2017.

[18] W. Huang, T. Liu, D. Wu, J. Cheng, Z. Ouyang, and C. Duan, "Field-induced slow relaxation of magnetization in a tetrahedral co (ii) complex with easy plane anisotropy," *Dalton Transactions*, vol. 42, no. 43, pp. 15326–15331, 2013.

[19] A. Eichhofer, Y. Lan, V. Mereacre, T. Bodenstein, and F. Weigend, "Slow magnetic relaxation in trigonal-planar mononuclear fe (ii) and co (ii) bis (trimethylsilyl) amido complexes a comparative study," *Inorganic Chemistry*, vol. 53, no. 4, pp. 1962–1974, 2014.

[20] M. A. Palacios, I. F. Díaz-Ortega, H. Nojiri, E. A. Suturina, M. Ozerov, J. Krzystek, and E. Colacio, "Tuning magnetic anisotropy by the π-bonding features of the axial ligands and the electronic effects of gold (i) atoms in 2d {Co (L) 2 [Au (CN) 2] 2} n metal–organic frameworks with field-induced single-ion magnet behaviour," *Inorganic Chemistry Frontiers*, vol. 7, no. 23, pp. 4611–4630, 2020.

[21] Y. Rechkemmer, F. D. Breitgoff, M. Van Der Meer, M. Atanasov, M. Hakl, M. Orlita, P. Neugebauer, F. Neese, B. Sarkar, and J. Van Slageren, "A four-coordinate cobalt (ii) single-ion magnet with coercivity and a very high energy barrier," *Nature communications*, vol. 7, no. 1, pp. 1–8, 2016.

[22] R. Herchel, L. Vahovska, I. Potocnak, and Z. Travnicek, "Slow magnetic relaxation in octahedral cobalt (ii) field-induced single-ion magnet with positive axial and large rhombic anisotropy," *Inorganic Chemistry*, vol. 53, no. 12, pp. 5896–5898, 2014.

[23] A. A. Pavlov, Y. V. Nelyubina, S. V. Kats, L. V. Penkova, N. N. Efimov, A. O. Dmitrienko, A. V. Vologzhanina, A. S. Belov, Y. Z. Voloshin, and V. V. Novikov, "Polymorphism in a cobalt-based single-ion magnet tuning its barrier to magnetization relaxation," *The journal of physical chemistry letters*, vol. 7, no. 20, pp. 4111–4116, 2016.

[24] L. Chen, J. Wang, J.-M. Wei, W. Wernsdorfer, X.-T. Chen, Y.-Q. Zhang, Y. Song, and Z.-L. Xue, "Slow magnetic relaxation in a mononuclear eight-coordinate cobalt (ii) complex," *Journal of the American Chemical Society*, vol. 136, no. 35, pp. 12213–12216, 2014.

[25] L. Rigamonti, N. Bridonneau, G. Poneti, L. Tesi, L. Sorace, D. Pinkowicz, J. Jover, E. Ruiz, R. Sessoli, and A. Cornia, "A pseudo-octahedral cobalt (ii) complex with bispyrazolylpyridine ligands acting as a zero-field single-molecule magnet with easy axis anisotropy," *Chemistry–A European Journal*, vol. 24, no. 35, pp. 8857–8868, 2018.

[26] A. K. Mondal, A. Mondal, B. Dey, and S. Konar, "Influence of the coordination environment on easy-plane magnetic anisotropy of pentagonal bipyramidal cobalt (ii) complexes," *Inorganic Chemistry*, vol. 57, no. 16, pp. 9999–10008, 2018.

[27] S. Ziegenbalg, D. Hornig, H. Gorls, and W. Plass, "Cobalt (ii)-based single-ion magnets with distorted pseudotetrahedral [n2o2] coordination: Experimental and theoretical investigations," *Inorganic Chemistry*, vol. 55, no. 8, pp. 4047–4058, 2016.

[28] F. Shao, B. Cahier, E. Riviere, R. Guillot, N. Guihery, V. E. Campbell, and T. Mallah, "Structural dependence of the ising-type magnetic anisotropy and of the relaxation time in mononuclear trigonal bipyramidal co (ii) single molecule magnets," *Inorganic chemistry*, vol. 56, no. 3, pp. 1104–1111, 2017.

[29] I. Nemec, R. Herchel, M. Kern, P. Neugebauer, J. Van Slageren, and Z. Trávníček, "Magnetic anisotropy and field-induced slow relaxation of magnetization in tetracoordinate coii compound [co (ch3-im) 2cl2]," *Materials*, vol. 10, no. 3, p. 249, 2017.

[30] A. K. Mondal, T. Goswami, A. Misra, and S. Konar, "Probing the effects of ligand field and coordination geometry on magnetic anisotropy of pentacoordinate cobalt (ii) single-ion magnets," *Inorganic Chemistry*, vol. 56, no. 12, pp. 6870–6878, 2017.

[31] C. Rajnák, F. Varga, J. Titis, J. Moncol, and R. Boca, "Octahedral–tetrahedral systems [co (dppm o, o) 3] 2+[cox4] 2–showing slow magnetic relaxation with two relaxation modes," *Inorganic Chemistry*, vol. 57, no. 8, pp. 4352–4358, 2018.

[32] P. C. Bunting, M. Atanasov, E. Damgaard-Møller, M. Perfetti, I. Crassee, M. Orlita, J. Overgaard, J. van Slageren, F. Neese, and J. R. Long, "A linear cobalt (ii) complex with maximal orbital angular momentum from a non-aufbau ground state," *Science*, vol. 362, no. 6421, p. eaat7319, 2018.

[33] K. Chakarawet, P. C. Bunting, and J. R. Long, "Large anisotropy barrier in a tetranuclear single-molecule magnet featuring low-coordinate cobalt centers," *Journal of the American Chemical Society*, vol. 140, no. 6, pp. 2058–2061, 2018.

[34] J. Zhou, J. Song, A. Yuan, Z. Wang, L. Chen, and Z.-W. Ouyang, "Slow magnetic relaxation in two octahedral cobalt (ii) complexes with positive axial anisotropy," *Inorganica Chimica Acta*, vol. 479, pp. 113–119, 2018.

[35] B. Na, X.-J. Zhang, W. Shi, Y.-Q. Zhang, B.-W. Wang, C. Gao, S. Gao, and P. Cheng, "Six-coordinate lanthanide complexes: Slow relaxation of magnetization in the dysprosium (iii) complex," *Chemistry–A European Journal*, vol. 20, no. 48, pp. 15975–15980, 2014.

[36] J. Liu, Y.-C. Chen, J.-L. Liu, V. Vieru, L. Ungur, J.-H. Jia, L. F. Chibotaru, Y. Lan, W. Wernsdorfer, S. Gao, *et al.*, "A stable pentagonal bipyramidal dy (iii) single-ion magnet with a record magnetization reversal barrier over 1000 k," *Journal of the American Chemical Society*, vol. 138, no. 16, pp. 5441–5450, 2016.

[37] Q.-Y. Liu, Y.-L. Li, W.-L. Xiong, Y.-L. Wang, F. Luo, C.-M. Liu, and L.-L. Chen, "Urothermal synthesis of mononuclear lanthanide compounds: slow magnetization relaxation observed in dy analogue," *CrystEngComm*, vol. 16, no. 4, pp. 585–590, 2014.

[38] S. N. König, N. F. Chilton, C. Maichle-Mössmer, E. M. Pineda, T. Pugh, R. Anwander, and R. A. Layfield, "Fast magnetic relaxation in an octahedral dysprosium tetramethyl-aluminate complex," *Dalton Transactions*, vol. 43, no. 8, pp. 3035–3038, 2014.

[39] Y.-C. Chen, J.-L. Liu, L. Ungur, J. Liu, Q.-W. Li, L.-F. Wang, Z.-P. Ni, L. F. Chibotaru, X.-M. Chen, and M.-L. Tong, "Symmetry-supported magnetic blocking at 20 k in pentagonal bipyramidal dy (iii) single-ion magnets," *Journal of the American Chemical Society*, vol. 138, no. 8, pp. 2829–2837, 2016.

[40] V. E. Campbell, H. Bolvin, E. Rivière, R. Guillot, W. Wernsdorfer, and T. Mallah, "Structural and electronic dependence of the single-molecule-magnet behavior of dysprosium (iii) complexes," *Inorganic Chemistry*, vol. 53, no. 5, pp. 2598–2605, 2014.

[41] T. J. Boyle, L. A. M. Ottley, and M. A. Rodriguez, "Structurally characterized group 4 metal alkoxides modified with the pyridyl-based ligands 2-mercapto pyridine n-oxide and 3, 3-dihydroxy-2, 2-bipyridine," *Polyhedron*, vol. 27, no. 14, pp. 3079–3084, 2008.

[42] J. Zhu, C. Wang, F. Luan, T. Liu, P. Yan, and G. Li, "Local coordination geometry perturbed β-diketone dysprosium single-ion magnets," *Inorganic Chemistry*, vol. 53, no. 17, pp. 8895–8901, 2014.

[43] H. Sun, M.-q. Liu, and B.-j. Zhang, "Two dysprosium complexes based on 8-hydroxyquinoline schiff base: Structures, luminescence properties and single-molecule magnets behaviors," *Inorganica Chimica Acta*, vol. 453, pp. 681–686, 2016.

[44] D. Hamada, T. Fujinami, S. Yamauchi, N. Matsumoto, N. Mochida, T. Ishida, Y. Sunatsuki, M. Tsuchimoto, C. Coletti, and N. Re, "Luminescent dyiii single ion magnets with same n6o3 donor atoms but different donor atom arrangements,'fac'-[dyiii (hldl-ala) 3]· 8h2o and 'mer'-[dyiii (hldl-phe) 3]· 7h2o," *Polyhedron*, vol. 109, pp. 120–128, 2016.

[45] P. Zhang, L. Zhang, C. Wang, S. Xue, S.-Y. Lin, and J. Tang, "Equatorially coordinated lanthanide single ion magnets," *Journal of the American Chemical Society*, vol. 136, no. 12, pp. 4484–4487, 2014.

[46] S. Wang, R. Ma, Z. Chen, Y. Li, T. Cao, C. Zhou, and J. Bai, "Solvent-and metal-directed lanthanide-organic frameworks based on pamoic acid: observation of slow magnetization relaxation, magnetocaloric effect and luminescent sensing," *Science China Chemistry*, vol. 59, no. 8, pp. 948–958, 2016.

[47] K. R. Meihaus, S. G. Minasian, W. W. Lukens Jr, S. A. Kozimor, D. K. Shuh, T. Tyliszczak, and J. R. Long, "Influence of pyrazolate vs n-heterocyclic carbene ligands on the slow magnetic relaxation of homoleptic trischelate lanthanide (iii) and uranium (iii) complexes," *Journal of the American Chemical Society*, vol. 136, no. 16, pp. 6056–6068, 2014.

[48] L. Sun, S. Zhang, C. Qiao, S. Chen, B. Yin, W. Wang, Q. Wei, G. Xie, and S. Gao, "Fine-tuning of the coordination environment to regulate the magnetic behavior in solvent/anion-dependent dyiii compounds: Synthesis, structure, magnetism, and ab initio calculations," *Inorganic Chemistry*, vol. 55, no. 20, pp. 10587–10596, 2016.

[49] S. Shintoyo, K. Murakami, T. Fujinami, N. Matsumoto, N. Mochida, T. Ishida, Y. Sunatsuki, M. Watanabe, M. Tsuchimoto, J. Mrozinski, *et al.*, "Crystal field splitting of the ground state of terbium (iii) and dysprosium (iii) complexes with a triimidazolyl tripod ligand and an acetate determined by magnetic analysis and luminescence," *Inorganic Chemistry*, vol. 53, no. 19, pp. 10359–10369, 2014.

[50] D.-P. Li, T.-W. Wang, C.-H. Li, D.-S. Liu, Y.-Z. Li, and X.-Z. You, "Single-ion magnets based on mononuclear lanthanide complexes with chiral schiff base ligands [ln (fta) 3l](ln= sm, eu, gd, tb and dy)," *Chemical communications*, vol. 46, no. 17, pp. 2929–2931, 2010.

[51] Y.-L. Chien, M.-W. Chang, Y.-C. Tsai, G.-H. Lee, W.-S. Sheu, and E.-C. Yang, "New salen-type dysprosium (iii) double-decker and triple-decker complexes," *Polyhedron*, vol. 102, pp. 8–15, 2015.

[52] Y.-S. Meng, C.-H. Wang, Y.-Q. Zhang, X.-B. Leng, B.-W. Wang, Y.-F. Chen, and S. Gao, "(boratabenzene)(cyclooctatetraenyl) lanthanide complexes: a new type of organometallic single-ion magnet," *Inorganic Chemistry Frontiers*, vol. 3, no. 6, pp. 828–835, 2016.

[53] W. Cao, C. Gao, Y.-Q. Zhang, D. Qi, T. Liu, K. Wang, C. Duan, S. Gao, and J. Jiang, "Rational enhancement of the energy barrier of bis (tetrapyrrole) dysprosium smms via replacing atom of porphyrin core," *Chemical science*, vol. 6, no. 10, pp. 5947–5954, 2015.

[54] P. Chen, M. Zhang, W. Sun, H. Li, L. Zhao, and P. Yan, "Anion-dependent assembly of dy complexes: structures and magnetic behaviors," *CrystEngComm*, vol. 17, no. 27, pp. 5066–5073, 2015.

[55] J.-K. Ou-Yang, N. Saleh, G. F. Garcia, L. Norel, F. Pointillart, T. Guizouarn, O. Cador, F. Totti, L. Ouahab, J. Crassous, *et al.*, "Improved slow magnetic relaxation in optically pure helicene-based dy iii single molecule magnets," *Chemical Communications*, vol. 52, no. 100, pp. 14474–14477, 2016.

[56] S.-Y. Lin, C. Wang, L. Zhao, J. Wu, and J. Tang, "Chiral mononuclear lanthanide complexes and the field-induced single-ion magnet behaviour of a dy analogue," *Dalton Transactions*, vol. 44, no. 1, pp. 223–229, 2015.

[57] A. K. Mondal, S. Goswami, and S. Konar, "Influence of the coordination environment on slow magnetic relaxation and photoluminescence behavior in two mononuclear dysprosium (iii) based single molecule magnets," *Dalton Transactions*, vol. 44, no. 11, pp. 5086–5094, 2015.

[58] Y.-S. Ding, T. Han, Y.-Q. Hu, M. Xu, S. Yang, and Y.-Z. Zheng, "Syntheses, structures and magnetic properties of a series of mono-and di-nuclear dysprosium (iii)-crown-ether complexes: effects of a weak ligand-field and flexible cyclic coordination modes," *Inorganic Chemistry Frontiers*, vol. 3, no. 6, pp. 798–807, 2016.

[59] Y. Wang, X.-L. Li, T.-W. Wang, Y. Song, and X.-Z. You, "Slow relaxation processes and single-ion magnetic behaviors in dysprosium-containing complexes," *Inorganic chemistry*, vol. 49, no. 3, pp. 969–976, 2010.

[60] F. Pointillart, J. Jung, R. Berraud-Pache, B. Le Guennic, V. Dorcet, S. Golhen, O. Cador, O. Maury, Y. Guyot, S. Decurtins, *et al.*, "Luminescence and single-molecule magnet behavior in lanthanide complexes involving a tetrathiafulvalene-fused dipyridophenazine ligand," *Inorganic chemistry*, vol. 54, no. 11, pp. 5384–5397, 2015.

[61] J.-R. Jiménez, I. F. Díaz-Ortega, E. Ruiz, D. Aravena, S. J. Pope, E. Colacio, and J. M. Herrera, "Lanthanide tetrazolate complexes combining single-molecule magnet and luminescence properties: The effect of the replacement of tetrazolate n3 by β-diketonate ligands on the anisotropy energy barrier," *Chemistry–A European Journal*, vol. 22, no. 41, pp. 14548–14559, 2016.

[62] K. Qian, J. J. Baldoví, S.-D. Jiang, A. Gaita-Arino, Y.-Q. Zhang, J. Overgaard, B.-W. Wang, E. Coronado, and S. Gao, "Does the thermal evolution of molecular structures critically affect the magnetic anisotropy?," *Chemical science*, vol. 6, no. 8, pp. 4587–4593, 2015.

[63] A. Lannes and D. Luneau, "New family of lanthanide-based complexes with different scorpionate-type ligands: a rare case where dysprosium and ytterbium analogues display single-ion-magnet behavior," *Inorganic Chemistry*, vol. 54, no. 14, pp. 6736–6743, 2015.

[64] S. Das, K. Bejoymohandas, A. Dey, S. Biswas, M. Reddy, R. Morales, E. Ruiz, S. Titos-Padilla, E. Colacio, and V. Chandrasekhar, "Amending the anisotropy barrier and luminescence behavior of heterometallic trinuclear linear [mii lniii mii](lniii= gd, tb, dy; mii= mg/zn) complexes by change from divalent paramagnetic to diamagnetic metal ions," *Chemistry–A European Journal*, vol. 21, no. 17, pp. 6449–6464, 2015.

[65] P. Selvanathan, G. Huang, T. Guizouarn, T. Roisnel, G. Fernandez-Garcia, F. Totti, B. Le Guennic, G. Calvez, K. Bernot, L. Norel, *et al.*, "Highly axial magnetic anisotropy in a n3o5 dysprosium (iii) coordination environment generated by a merocyanine ligand," *Chemistry–A European Journal*, vol. 22, no. 43, pp. 15222–15226, 2016.

[66] Y. Dong, P. Yan, X. Zou, and G. Li, "Azacyclo-auxiliary ligand-tuned smms of dibenzoylmethane dy (iii) complexes," *Inorganic Chemistry Frontiers*, vol. 2, no. 9, pp. 827–836, 2015.

[67] P. Zhang, J. Jung, L. Zhang, J. Tang, and B. Le Guennic, "Elucidating the magnetic anisotropy and relaxation dynamics of low-coordinate lanthanide compounds," *Inorganic Chemistry*, vol. 55, no. 4, pp. 1905–1911, 2016.

[68] Y. Bi, C. Chen, Y.-F. Zhao, Y.-Q. Zhang, S.-D. Jiang, B.-W. Wang, J.-B. Han, J.-L. Sun, Z.-Q. Bian, Z.-M. Wang, *et al.*, "Thermostability and photoluminescence of dy (iii) single-molecule magnets under a magnetic field," *Chemical science*, vol. 7, no. 8, pp. 5020–5031, 2016.

[69] F. Duan, L. Liu, C. Qiao, and H. Yang, "Self-assembly and magnetic behavior of 2-aldehyde-8-hydroxyquinolinate-based lanthanide complex," *Inorganic Chemistry Communications*, vol. 55, pp. 120–122, 2015.

[70] S. K. Gupta, T. Rajeshkumar, G. Rajaraman, and R. Murugavel, "An air-stable dy (iii) single-ion magnet with high anisotropy barrier and blocking temperature," *Chemical science*, vol. 7, no. 8, pp. 5181–5191, 2016.

[71] Y. Dong, P. Yan, X. Zou, T. Liu, and G. Li, "Exploiting single-molecule magnets of β-diketone dysprosium complexes with c 3v symmetry: suppression of quantum tunneling of magnetization," *Journal of Materials Chemistry C*, vol. 3, no. 17, pp. 4407–4415, 2015.

[72] C. Gao, Q. Yang, B.-W. Wang, Z.-M. Wang, and S. Gao, "Evaporable lanthanide single-ion magnet," *CrystEngComm*, vol. 18, no. 22, pp. 4165–4171, 2016.

[73] M. Ren, Z.-L. Xu, S.-S. Bao, T.-T. Wang, Z.-H. Zheng, R. A. Ferreira, L.-M. Zheng, and L. D. Carlos, "Lanthanide salen-type complexes exhibiting single ion magnet and photoluminescent properties," *Dalton Transactions*, vol. 45, no. 7, pp. 2974–2982, 2016.

[74] E. L. Gavey, M. Al Hareri, J. Regier, L. D. Carlos, R. A. Ferreira, F. S. Razavi, J. M. Rawson, and M. Pilkington, "Placing a crown on dy iii–a dual property ln iii crown ether complex displaying optical properties and smm behaviour," *Journal of Materials Chemistry C*, vol. 3, no. 29, pp. 7738–7747, 2015.

[75] S. Zhang, H. Ke, L. Sun, X. Li, Q. Shi, G. Xie, Q. Wei, D. Yang, W. Wang, and S. Chen, "Magnetization dynamics changes of dysprosium (iii) single-ion magnets associated with guest molecules," *Inorganic Chemistry*, vol. 55, no. 8, pp. 3865–3871, 2016.

[76] J. Jung, X. Yi, G. Huang, G. Calvez, C. Daiguebonne, O. Guillou, O. Cador, A. Caneschi, T. Roisnel, B. Le Guennic, *et al.*, "Analysis of the electrostatics in dy iii single-molecule magnets: the case study of dy (murex) 3," *Dalton Transactions*, vol. 44, no. 41, pp. 18270–18275, 2015.

[77] Y. Lan, A. Magri, O. Fuhr, and M. Ruben, "Phenalenyl-based mononuclear dysprosium complexes," *Beilstein journal of nanotechnology*, vol. 7, no. 1, pp. 995–1009, 2016.

[78] S. Zhang, H. Ke, Q. Shi, J. Zhang, Q. Yang, Q. Wei, G. Xie, W. Wang, D. Yang, and S. Chen, "Dysprosium (iii) complexes with a square-antiprism configuration featuring mononuclear single-molecule magnetic behaviours based on different β-diketonate ligands and auxiliary ligands," *Dalton Transactions*, vol. 45, no. 12, pp. 5310–5320, 2016.

[79] K. R. Meihaus, J. D. Rinehart, and J. R. Long, "Dilution-induced slow magnetic relaxation and anomalous hysteresis in trigonal prismatic dysprosium (iii) and uranium (iii) complexes," *Inorganic Chemistry*, vol. 50, no. 17, pp. 8484–8489, 2011.

[80] S.-S. Liu, K. Lang, Y.-Q. Zhang, Q. Yang, B.-W. Wang, and S. Gao, "A distinct magnetic anisotropy enhancement in mononuclear dysprosium–sulfur complexes by controlling the dy-ligand bond length," *Dalton Transactions*, vol. 45, no. 19, pp. 8149–8153, 2016.

[81] Z.-X. Jiang, J.-L. Liu, Y.-C. Chen, J. Liu, J.-H. Jia, and M.-L. Tong, "Lanthanoid single-ion magnets with the lnn 10 coordination geometry," *Chemical Communications*, vol. 52, no. 37, pp. 6261–6264, 2016.

[82] D.-P. Li, X.-P. Zhang, T.-W. Wang, B.-B. Ma, C.-H. Li, Y.-Z. Li, and X.-Z. You, "Distinct magnetic dynamic behavior for two polymorphs of the same dy (iii) complex," *Chemical Communications*, vol. 47, no. 24, pp. 6867–6869, 2011.

[83] Y. Dong, P. Yan, X. Zou, X. Yao, G. Hou, and G. Li, "Auxiliary ligand field dominated single-molecule magnets of a series of indole-derivative β-diketone mononuclear dy (iii) complexes," *Dalton Transactions*, vol. 45, no. 22, pp. 9148–9157, 2016.

[84] S. Goswami, S. Biswas, K. Tomar, and S. Konar, "Tuning the magnetoluminescence behavior of lanthanide complexes having sphenocorona and cubic coordination geometries," *European Journal of Inorganic Chemistry*, vol. 2016, no. 17, pp. 2774–2782, 2016.

[85] G.-J. Chen, C.-Y. Gao, J.-L. Tian, J. Tang, W. Gu, X. Liu, S.-P. Yan, D.-Z. Liao, and P. Cheng, "Coordination-perturbed single-molecule magnet behaviour of mononuclear dysprosium complexes," *Dalton Transactions*, vol. 40, no. 20, pp. 5579–5583, 2011.

[86] P. Antal, B. Drahoš, R. Herchel, and Z. Trávníček, "Muffin-like lanthanide complexes with an n 5 o 2-donor macrocyclic ligand showing field-induced single-molecule magnet behaviour," *Dalton Transactions*, vol. 45, no. 38, pp. 15114–15121, 2016.

[87] B. Yao, B. Gu, M. Su, G. Li, Y. Ma, L. Li, Q. Wang, P. Cheng, and X. Zhang, "Single-molecule magnet behavior in a mononuclear dysprosium (iii) complex with 1-methylimidazole," *RSC advances*, vol. 7, no. 5, pp. 2766–2772, 2017.

[88] F. Gao, F.-L. Yang, X. Feng, H. Xu, W. Sun, H. Liu, and X.-L. Li, "Half-sandwich lanthanide crown ether complexes with the slow relaxation of magnetization and photoluminescence behaviors," *Dalton Transactions*, vol. 46, no. 4, pp. 1317–1323, 2017.

[89] Y. Bi, Y.-N. Guo, L. Zhao, Y. Guo, S.-Y. Lin, S.-D. Jiang, J. Tang, B.-W. Wang, and S. Gao, "Capping ligand perturbed slow magnetic relaxation in dysprosium single-ion magnets," *Chemistry–A European Journal*, vol. 17, no. 44, pp. 12476–12481, 2011.

[90] X.-L. Li, J. Li, C. Zhu, B. Han, Y. Liu, Z. Yin, F. Li, and C.-M. Liu, "An intense luminescent dy (iii) single-ion magnet with the acylpyrazolonate ligand showing two slow magnetic relaxation processes," *New Journal of Chemistry*, vol. 42, no. 20, pp. 16992–16998, 2018.

[91] H.-R. Wen, K. Yang, S.-J. Liu, F.-Y. Liang, X.-R. Xie, C.-M. Liu, and Y.-W. Li, "Chiral mononuclear dy (iii) complex based on pyrrolidine-dithiocarboxylate s-donors with field-induced single-ion magnet behavior," *Inorganica Chimica Acta*, vol. 473, pp. 145–151, 2018.

[92] P.-P. Cen, S. Zhang, X.-Y. Liu, W.-M. Song, Y.-Q. Zhang, G. Xie, and S.-P. Chen, "Electrostatic potential determined magnetic dynamics observed in two mononuclear β-diketone dysprosium (iii) single-molecule magnets," *Inorganic Chemistry*, vol. 56, no. 6, pp. 3644–3656, 2017.

[93] P. Hu, M. Zhu, X. Mei, H. Tian, Y. Ma, L. Li, and D. Liao, "Single-molecule magnets based on rare earth complexes with chelating benzimidazole-substituted nitronyl nitroxide radicals," *Dalton Transactions*, vol. 41, no. 48, pp. 14651–14656, 2012.

[94] A. Upadhyay, S. K. Singh, C. Das, R. Mondol, S. K. Langley, K. S. Murray, G. Rajaraman, and M. Shanmugam, "Enhancing the effective energy barrier of a dy (iii) smm using a bridged diamagnetic zn (ii) ion," *Chemical Communications*, vol. 50, no. 64, pp. 8838–8841, 2014.

[95] J. Long, I. V. Basalov, N. V. Forosenko, K. A. Lyssenko, E. Mamontova, A. V. Cherkasov, M. Damjanović, L. F. Chibotaru, Y. Guari, J. Larionova, *et al.*, "Dysprosium single-molecule magnets with bulky schiff base ligands: Modification of the slow relaxation of the magnetization by substituent change," *Chemistry–A European Journal*, vol. 25, no. 2, pp. 474–478, 2019.

[96] Y.-C. Chen, J.-L. Liu, Y. Lan, Z.-Q. Zhong, A. Mansikkamäki, L. Ungur, Q.-W. Li, J.-H. Jia, L. F. Chibotaru, J.-B. Han, *et al.*, "Dynamic magnetic and optical insight into a high performance pentagonal bipyramidal dyiii single-ion magnet," *Chemistry–A European Journal*, vol. 23, no. 24, pp. 5708–5715, 2017.

[97] H.-R. Wen, F.-Y. Liang, Z.-G. Zou, S.-J. Liu, J.-S. Liao, and J.-L. Chen, "Mononuclear dy (iii) complex based on bipyridyl-tetrazolate ligand with field-induced single-ion magnet behavior and luminescent properties," *Inorganic Chemistry Communications*, vol. 79, pp. 41–45, 2017.

[98] P. Cen, X. Liu, J. Ferrando-Soria, Y.-Q. Zhang, G. Xie, S. Chen, and E. Pardo, "Capping n-donor ligands modulate the magnetic dynamics of dyiii β-diketonate single-ion magnets with d4d symmetry," *Chemistry–A European Journal*, vol. 25, no. 15, pp. 3884–3892, 2019.

[99] M. Jeletic, P.-H. Lin, J. J. Le Roy, I. Korobkov, S. I. Gorelsky, and M. Murugesu, "An organometallic sandwich lanthanide single-ion magnet with an unusual multiple relaxation mechanism," *Journal of the American Chemical Society*, vol. 133, no. 48, pp. 19286–19289, 2011.

[100] V. D. Sasnovskaya, V. A. Kopotkov, A. V. Kazakova, A. D. Talantsev, R. B. Morgunov, S. V. Simonov, L. V. Zorina, V. S. Mironov, and E. B. Yagubskii, "Slow magnetic relaxation in mononuclear complexes of tb, dy, ho and er with the pentadentate (n 3 o 2) schiff-base dapsc ligand," *New Journal of Chemistry*, vol. 42, no. 18, pp. 14883–14893, 2018.

[101] S. Biswas, K. S. Bejoymohandas, S. Das, P. Kalita, M. L. Reddy, I. Oyarzabal, E. Colacio, and V. Chandrasekhar, "Mononuclear lanthanide complexes: energy-barrier enhancement by ligand substitution in field-induced dyiii sims," *Inorganic Chemistry*, vol. 56, no. 14, pp. 7985–7997, 2017.

[102] Z.-H. Li, Y.-Q. Zhai, W.-P. Chen, Y.-S. Ding, and Y.-Z. Zheng, "Air-stable hexagonal bipyramidal dysprosium (iii) single-ion magnets with nearly perfect d6h local symmetry," *Chemistry–A European Journal*, vol. 25, no. 71, pp. 16219–16224, 2019.

[103] S. P. Petrosyants, Z. V. Dobrokhotova, A. B. Ilyukhin, N. N. Efimov, A. V. Gavrikov, P. N. Vasilyev, and V. M. Novotortsev, "Mononuclear dysprosium thiocyanate complexes with 2, 2-bipyridine and 1, 10-phenanthroline: Synthesis, crystal structures, sim behavior, and solid-phase transformations," *European Journal of Inorganic Chemistry*, vol. 2017, no. 29, pp. 3561–3569, 2017.

[104] Z. Liang, M. Damjanovic, M. Kamila, G. Cosquer, B. K. Breedlove, M. Enders, and M. Yamashita, "Proton control of the lanthanoid single-ion magnet behavior of a double-decker complex with an indolenine-substituted annulene ligand," *Inorganic chemistry*, vol. 56, no. 11, pp. 6512–6521, 2017.

[105] C. A. Goodwin, F. Ortu, D. Reta, N. F. Chilton, and D. P. Mills, "Molecular magnetic hysteresis at 60 kelvin in dysprosocenium," *Nature*, vol. 548, no. 7668, pp. 439–442, 2017.

[106] L.-L. Li, H.-D. Su, S. Liu, Y.-C. Xu, and W.-Z. Wang, "A new air-and moisture-stable pentagonal-bipyramidal dy iii single-ion magnet based on the hmpa ligand," *Dalton Transactions*, vol. 48, no. 6, pp. 2213–2219, 2019.

[107] P. Hu, F.-P. Xiao, Y. Li, J.-F. Cao, Z.-S. Chen, L.-L. Zhu, and W.-P. Huang, "One mononuclear single-molecule magnet derived from dy (iii) and dmbpy (dmbpy= 4, 4-dimethyl-2, 2-dipyridyl)," *Inorganic Chemistry Communications*, vol. 84, pp. 207–211, 2017.

[108] A. Bhunia, M. T. Gamer, L. Ungur, L. F. Chibotaru, A. K. Powell, Y. Lan, P. W. Roesky, F. Menges, C. Riehn, and G. Niedner-Schatteburg, "From a dy (iii) single molecule magnet (smm) to a ferromagnetic [mn (ii) dy (iii) mn (ii)] trinuclear complex," *Inorganic chemistry*, vol. 51, no. 18, pp. 9589–9597, 2012.

[109] Y. Dong, W. Li, X. Zou, G. Hou, and G. Li, "Electron-donating effect dominated 5, 6-dimethoxy-2-(2, 2, 2-trifluoroethyl)-1-indone dysprosium smm," *Inorganica Chimica Acta*, vol. 466, pp. 599–603, 2017.

[110] Q. Zou, X.-D. Huang, J.-C. Liu, S.-S. Bao, and L.-M. Zheng, "Lanthanide anthracene complexes: slow magnetic relaxation and luminescence in dy iii, er iii and yb iii based materials," *Dalton Transactions*, vol. 48, no. 8, pp. 2735–2740, 2019.

[111] M. Menelaou, F. Ouharrou, L. Rodríguez, O. Roubeau, S. J. Teat, and N. Aliaga-Alcalde, "Dyiii- and ybiii-curcuminoid compounds: Original fluorescent single-ion magnet and magnetic near-ir luminescent species," *Chemistry–A European Journal*, vol. 18, no. 37, pp. 11545–11549, 2012.

[112] P. Kalita, A. Malakar, J. Goura, S. Nayak, J. M. Herrera, E. Colacio, and V. Chandrasekhar, "Mononuclear lanthanide complexes assembled from a tridentate nno donor ligand: design of a dy iii single-ion magnet," *Dalton Transactions*, vol. 48, no. 15, pp. 4857–4866, 2019.

[113] J. Wu, O. Cador, X.-L. Li, L. Zhao, B. Le Guennic, and J. Tang, "Axial ligand field in d 4 d coordination symmetry: Magnetic relaxation of dy smms perturbed by counteranions," *Inorganic Chemistry*, vol. 56, no. 18, pp. 11211–11219, 2017.

[114] S. Zhang, W. Mo, Z. Zhang, F. Gao, L. Wang, D. Hu, and S. Chen, "Ligand ratio/solvent-influenced syntheses, crystal structures, and magnetic properties of polydentate schiff base ligand-dy (iii) compounds with β-diketonate ligands as co-ligands," *Dalton Transactions*, vol. 48, no. 33, pp. 12466–12481, 2019.

[115] S. Yu, Z. Chen, H. Hu, B. Li, Y. Liang, D. Liu, H. Zou, D. Yao, and F. Liang, "Two mononuclear dysprosium (iii) complexes with their slow magnetic relaxation behaviors tuned by coordination geometry," *Dalton Transactions*, vol. 48, no. 44, pp. 16679–16686, 2019.

[116] L. Chen, J. Zhou, A. Yuan, and Y. Song, "Slow magnetic relaxation in luminescent mononuclear dysprosium (iii) and erbium (iii) pentanitrate complexes with the same lno 10 coordination geometry," *Dalton Transactions*, vol. 46, no. 45, pp. 15812–15818, 2017.

[117] S.-D. Jiang, S.-S. Liu, L.-N. Zhou, B.-W. Wang, Z.-M. Wang, and S. Gao, "Series of lanthanide organometallic single-ion magnets," *Inorganic chemistry*, vol. 51, no. 5, pp. 3079–3087, 2012.

[118] L.-W. Cheng, C.-L. Zhang, J.-Y. Wei, and P.-H. Lin, "Mononuclear and trinuclear dy iii smms with schiff-base ligands modified by nitro-groups: first triangular complex with a n–n pathway," *Dalton Transactions*, vol. 48, no. 46, pp. 17331–17339, 2019.

[119] A. B. Canaj, M. K. Singh, C. Wilson, G. Rajaraman, and M. Murrie, "Chemical and in silico tuning of the magnetisation reversal barrier in pentagonal bipyramidal dy (iii) single-ion magnets," *Chemical Communications*, vol. 54, no. 59, pp. 8273–8276, 2018.

[120] H.-R. Wen, J.-L. Zhang, F.-Y. Liang, K. Yang, S.-J. Liu, and C.-M. Liu, "Multifunctional lanthanide complexes based on tetraazacyclolamidophenol ligand with field-induced slow magnetic relaxation, luminescent and shg properties," *European Journal of Inorganic Chemistry*, vol. 2019, no. 10, pp. 1406–1412, 2019.

[121] B. M. Day, F.-S. Guo, S. R. Giblin, A. Sekiguchi, A. Mansikkamäki, and R. A. Layfield, "Rare-earth cyclobutadienyl sandwich complexes: synthesis, structure and dynamic magnetic properties," *Chemistry–A European Journal*, vol. 24, no. 63, pp. 16779–16782, 2018.

[122] H.-H. Zou, T. Meng, Q. Chen, Y.-Q. Zhang, H.-L. Wang, B. Li, K. Wang, Z.-L. Chen, and F. Liang, "Bifunctional mononuclear dysprosium complexes: single-ion magnet behaviors and antitumor activities," *Inorganic Chemistry*, vol. 58, no. 4, pp. 2286–2298, 2019.

[123] P. Selvanathan, V. Dorcet, T. Roisnel, K. Bernot, G. Huang, B. Le Guennic, L. Norel, and S. Rigaut, "trans to cis photo-isomerization in merocyanine dysprosium and yttrium complexes," *Dalton Transactions*, vol. 47, no. 12, pp. 4139–4148, 2018.

[124] G. Lu, J. Wang, C.-J. Zhang, S. Bala, Y.-C. Chen, G.-Z. Huang, Z.-P. Ni, and M.-L. Tong, "Single-ion magnet and luminescent properties in a dy (iii) triangular dodecahedral complex," *Inorganic Chemistry Communications*, vol. 102, pp. 16–19, 2019.

[125] X.-X. Chen, F. Ma, M.-X. Xu, J.-C. Bi, H.-L. Sun, B.-W. Wang, and S. Gao, "A neutral auxiliary ligand enhanced dysprosium (iii) single molecule magnet," *Dalton Transactions*, vol. 47, no. 22, pp. 7395–7398, 2018.

[126] H. Yang, S.-S. Liu, Y.-S. Meng, Y.-Q. Zhang, L. Pu, and X.-Q. Yu, "Magnetic properties and theoretical calculations of mononuclear lanthanide complexes with a schiff base coordinated to ln (iii) ion in a monodentate coordination mode," *Inorganica Chimica Acta*, vol. 494, pp. 8–12, 2019.

[127] D. Maniaki, I. Mylonas-Margaritis, J. Mayans, A. Savvidou, C. P. Raptopoulou, V. Bekiari, V. Psycharis, A. Escuer, and S. P. Perlepes, "Slow magnetic relaxation and luminescence properties in lanthanide (iii)/anil complexes," *Dalton Transactions*, vol. 47, no. 34, pp. 11859–11872, 2018.

[128] P. Evans, D. Reta, G. F. Whitehead, N. F. Chilton, and D. P. Mills, "Bis-monophospholyl dysprosium cation showing magnetic hysteresis at 48 k," *Journal of the American Chemical Society*, vol. 141, no. 50, pp. 19935–19940, 2019.

[129] H.-L. Wang, X.-F. Ma, H.-H. Zou, K. Wang, B. Li, Z.-L. Chen, and F.-P. Liang, "Mixed chelating ligands used to regulate the luminescence of ln (iii) complexes and single-ion magnet behavior in dy-based analogues," *Dalton Transactions*, vol. 47, no. 44, pp. 15929–15940, 2018.

[130] J. Ruiz, A. J. Mota, A. Rodríguez-Diéguez, S. Titos, J. M. Herrera, E. Ruiz, E. Cremades, J. P. Costes, and E. Colacio, "Field and dilution effects on the slow relaxation of a luminescent dyo 9 low-symmetry single-ion magnet," *Chemical Communications*, vol. 48, no. 64, pp. 7916–7918, 2012.

[131] D. Guettas, V. Montigaud, G. F. Garcia, P. Larini, O. Cador, B. Le Guennic, and G. Pilet, "Fine control of the metal environment within dysprosium-based mononuclear single-molecule magnets," *European Journal of Inorganic Chemistry*, vol. 2018, no. 3-4, pp. 333–339, 2018.

[132] X.-L. Li, C.-L. Chen, Y.-L. Gao, C.-M. Liu, X.-L. Feng, Y.-H. Gui, and S.-M. Fang, "Modulation of homochiral dyiii complexes: Single-molecule magnets with ferroelectric properties," *Chemistry–A European Journal*, vol. 18, no. 46, pp. 14632–14637, 2012.

[133] N. F. Chilton, S. K. Langley, B. Moubaraki, A. Soncini, S. R. Batten, and K. S. Murray, "Single molecule magnetism in a family of mononuclear β-diketonate lanthanide (iii) complexes: rationalization of magnetic anisotropy in complexes of low symmetry," *Chemical Science*, vol. 4, no. 4, pp. 1719–1730, 2013.

[134] Y. Kishi, L. Cornet, F. Pointillart, F. Riobé, B. Lefeuvre, O. Cador, B. Le Guennic, O. Maury, H. Fujiwara, and L. Ouahab, "Luminescence and single-molecule-magnet behaviour in lanthanide coordination complexes involving benzothiazole-based tetrathiafulvalene ligands," *European Journal of Inorganic Chemistry*, vol. 2018, no. 3-4, pp. 458–468, 2018.

[135] M. U. Anwar, L. N. Dawe, S. S. Tandon, S. D. Bunge, and L. K. Thompson, "Polynuclear lanthanide (ln) complexes of a tri-functional hydrazone ligand–mononuclear (dy), dinuclear (yb, tm), tetranuclear (gd), and hexanuclear (gd, dy, tb) examples," *Dalton Transactions*, vol. 42, no. 21, pp. 7781–7794, 2013.

[136] X.-L. Mei, Y. Ma, L.-C. Li, and D.-Z. Liao, "Ligand field-tuned single-molecule magnet behaviour of 2p–4f complexes," *Dalton Transactions*, vol. 41, no. 2, pp. 505–511, 2012.

[137] F. Völcker, Y. Lan, A. K. Powell, and P. W. Roesky, "Slow magnetic relaxation in tris (diphosphanylamido) and tetra (phosphanoamido) dysprosium complexes," *Dalton Transactions*, vol. 42, no. 32, pp. 11471–11475, 2013.

[138] F. R. Fortea-Perez, J. Vallejo, M. Julve, F. Lloret, G. De Munno, D. Armentano, and E. Pardo, "Slow magnetic relaxation in a hydrogen-bonded 2d array of mononuclear dysprosium (iii) oxamates," *Inorganic Chemistry*, vol. 52, no. 9, pp. 4777–4779, 2013.

[139] W. Liu, S. Zeng, X. Chen, H. Pan, D. Qi, K. Wang, J. Dou, and J. Jiang, "Hemiporphyrazine-involved sandwich dysprosium double-decker single-ion magnets," *Inorganic Chemistry*, vol. 57, no. 19, pp. 12347–12353, 2018.

[140] V. E. Campbell, R. Guillot, E. Riviere, P.-T. Brun, W. Wernsdorfer, and T. Mallah, "Subcomponent self-assembly of rare-earth single-molecule magnets," *Inorganic Chemistry*, vol. 52, no. 9, pp. 5194–5200, 2013.

[141] J. J. Le Roy, M. Jeletic, S. I. Gorelsky, I. Korobkov, L. Ungur, L. F. Chibotaru, and M. Murugesu, "An organometallic building block approach to produce a multidecker 4 f single-molecule magnet," *Journal of the American Chemical Society*, vol. 135, no. 9, pp. 3502–3510, 2013.

[142] W. Cañon-Mancisidor, S. G. Miralles, J. J. Baldoví, G. M. Espallargas, A. Gaita-Arino, and E. Coronado, "Sublimable single ion magnets based on lanthanoid quinolinate complexes: The role of intermolecular interactions on their thermal stability," *Inorganic Chemistry*, vol. 57, no. 22, pp. 14170–14177, 2018.

[143] G. Cosquer, F. Pointillart, S. Golhen, O. Cador, and L. Ouahab, "Slow magnetic relaxation in condensed versus dispersed dysprosium (iii) mononuclear complexes," *Chemistry–A European Journal*, vol. 19, no. 24, pp. 7895–7903, 2013.

[144] S. Goswami, A. Adhikary, H. S. Jena, and S. Konar, "Lanthanide based coordination polymers chill, relax under magnetic field and also fluoresce," *Dalton Transactions*, vol. 42, no. 27, pp. 9813–9817, 2013.

[145] C.-L. Ji, Y.-X. Jiang, J.-C. Zhang, Z.-Y. Qi, J.-J. Kong, and X.-C. Huang, "Field-induced slow magnetic relaxation behavior in a mononuclear dy (iii) complex based on 8-hydroxyquinoline derivate ligand," *Zeitschrift für anorganische und allgemeine Chemie*, vol. 644, no. 23, pp. 1635–1640, 2018.

[146] Y.-L. Wang, Y. Ma, X. Yang, J. Tang, P. Cheng, Q.-L. Wang, L.-C. Li, and D.-Z. Liao, "Syntheses, structures, and magnetic and luminescence properties of a new dyiii-based single-ion magnet," *Inorganic Chemistry*, vol. 52, no. 13, pp. 7380–7386, 2013.

[147] Y.-X. Wang, Y. Ma, Y. Chai, W. Shi, Y. Sun, and P. Cheng, "Observation of magnetodielectric effect in a dysprosium-based single-molecule magnet," *Journal of the American Chemical Society*, vol. 140, no. 25, pp. 7795–7798, 2018.

[148] K. R. Meihaus and J. R. Long, "Magnetic blocking at 10 k and a dipolar-mediated avalanche in salts of the bis (η8-cyclooctatetraenide) complex [er (cot) 2]-," *Journal of the American Chemical Society*, vol. 135, no. 47, pp. 17952–17957, 2013.

[149] P.-H. Lin, I. Korobkov, T. J. Burchell, and M. Murugesu, "Connecting single-ion magnets through ligand dimerisation," *Dalton Transactions*, vol. 41, no. 44, pp. 13649–13655, 2012.

[150] B. Na, Y.-X. Wang, T. Han, W. Shi, and P. Cheng, "A rare one-dimensional dy (iii) complex exhibiting slow magnetic relaxation," *Inorganic Chemistry Communications*, vol. 35, pp. 19–21, 2013.

[151] Z. Jiang, L. Sun, Q. Yang, B. Yin, H. Ke, J. Han, Q. Wei, G. Xie, and S. Chen, "Excess axial electrostatic repulsion as a criterion for pentagonal bipyramidal dy iii single-ion magnets with high u eff and tb," *Journal of Materials Chemistry C*, vol. 6, no. 15, pp. 4273–4280, 2018.

[152] L. Ungur, J. J. Le Roy, I. Korobkov, M. Murugesu, and L. F. Chibotaru, "Fine-tuning the local symmetry to attain record blocking temperature and magnetic remanence in a single-ion magnet," *Angewandte Chemie*, vol. 126, no. 17, pp. 4502–4506, 2014.

[153] X. Li, T. Li, X. J. Shi, and L. Tian, "A family of 2p-4f complexes based on indazole radical: Syntheses, structures and magnetic properties," *Inorganica Chimica Acta*, vol. 456, pp. 216–223, 2017.

[154] S.-S. Liu, J. W. Ziller, Y.-Q. Zhang, B.-W. Wang, W. J. Evans, and S. Gao, "A half-sandwich organometallic single-ion magnet with hexamethylbenzene coordinated to the dy (iii) ion," *Chemical Communications*, vol. 50, no. 77, pp. 11418–11420, 2014.
